# Molecular Epidemiology of Citrus Leprosis Virus C: A New Viral Lineage and Phylodynamic of the Main Viral Subpopulations in the Americas

**DOI:** 10.3389/fmicb.2021.641252

**Published:** 2021-04-29

**Authors:** Camila Chabi-Jesus, Pedro L. Ramos-González, Matheus Postclam-Barro, Rafaela Salgado Fontenele, Ricardo Harakava, Renato B. Bassanezi, Alecio S. Moreira, Elliot W. Kitajima, Arvind Varsani, Juliana Freitas-Astúa

**Affiliations:** ^1^Escola Superior de Agricultura “Luiz de Queiroz”, University of São Paulo, São Paulo, Brazil; ^2^Instituto Biológico/IB, São Paulo, Brazil; ^3^The Biodesign Center for Fundamental and Applied Microbiomics, Center for Evolution and Medicine, School of Life Sciences, Arizona State University, Tempe, AZ, United States; ^4^Fundo de Defesa da Citricultura, Araraquara, Brazil; ^5^Embrapa Mandioca e Fruticultura, Cruz das Almas, Brazil; ^6^Structural Biology Research Unit, Department of Integrative Biomedical Sciences, University of Cape Town, Observatory, Cape Town, South Africa

**Keywords:** citrus leprosis disease, *Cilevirus*, *Kitaviridae*, *Brevipalpus* mites, virus evolution

## Abstract

Despite the importance of viral strains/variants as agents of emerging diseases, genetic and evolutionary processes affecting their ecology are not fully understood. To get insight into this topic, we assessed the population and spatial dynamic parameters of citrus leprosis virus C (CiLV-C, genus *Cilevirus*, family *Kitaviridae*). CiLV-C is the etiological agent of citrus leprosis disease, a non-systemic infection considered the main viral disorder affecting citrus orchards in Brazil. Overall, we obtained 18 complete or near-complete viral genomes, 123 complete nucleotide sequences of the open reading frame (ORF) encoding the putative coat protein, and 204 partial nucleotide sequences of the ORF encoding the movement protein, from 430 infected *Citrus* spp. samples collected between 1932 and 2020. A thorough examination of the collected dataset suggested that the CiLV-C population consists of the major lineages CRD and SJP, unevenly distributed, plus a third one called ASU identified in this work, which is represented by a single isolate found in an herbarium sample collected in Asuncion, Paraguay, in 1937. Viruses from the three lineages share about 85% nucleotide sequence identity and show signs of inter-clade recombination events. Members of the lineage CRD were identified both in commercial and non-commercial citrus orchards. However, those of the lineages SJP were exclusively detected in samples collected in the citrus belt of São Paulo and Minas Gerais, the leading Brazilian citrus production region, after 2015. The most recent common ancestor of viruses of the three lineages dates back to, at least, ∼1500 years ago. Since citrus plants were introduced in the Americas by the Portuguese around the 1520s, the Bayesian phylodynamic analysis suggested that the ancestors of the main CiLV-C lineages likely originated in contact with native vegetation of South America. The intensive expansion of CRD and SJP lineages in Brazil started probably linked to the beginning of the local citrus industry. The high prevalence of CiLV-C in the citrus belt of Brazil likely ensues from the intensive connectivity between orchards, which represents a potential risk toward pathogen saturation across the region.

## Introduction

Brazil is the leading sweet orange producer in the world. With almost 197.7 million sweet orange (*Citrus x sinensis (L.) Osbeck*) trees^1^, the citrus belt São Paulo (SP)—Minas Gerais (MG) is the largest citrus cultivation area in South America and accounts for more than 80% of the Brazilian sweet orange production (Bassanezi et al., 2019). Citrus orchard yields may be impacted by citrus leprosis (CL) disease, ranked first among the viral diseases affecting this crop in Brazil (Ramos-González et al., 2018). Control of CL reaches up to US$ 54 million/year, a value representing about 5% of the management cost of orchards in the main Brazilian citrus belt (Bassanezi et al., 2019).

Despite the multi-etiological character of CL, citrus leprosis virus C (CiLV-C) is, by far, the prevalent causal agent in Brazil (Ramos-González et al., 2016, 2017, 2018; Chabi-Jesus et al., 2018). The virus infects several species within the genus *Citrus* and their hybrids, although with different degrees of severity. While sweet oranges show high susceptibility, mandarins (*C. reshni*, *C. reticulata*, and *C. deliciosa*) are moderately resistant, and lemons (*C. limon*) and limes (*C. aurantifolia*) are considered resistant (Bastianel et al., 2018). CiLV-C also naturally infects *Commelina benghalensis* and *Swinglea glutinosa* and can be experimentally transmitted to plants of 28 families (León et al., 2008; Nunes et al., 2012; Garita et al., 2014; Arena et al., 2017).

*Citrus leprosis virus C* is the type species of the genus *Cilevirus*, family *Kitaviridae* (Locali-Fabris et al., 2006, 2012; Freitas-Astúa et al., 2018). In addition to cileviruses, the family also includes members of the genera *Higrevirus* and *Blunervirus* (Melzer et al., 2012; Quito-Avila et al., 2013). Kitaviruses have bacilliform or spherical virions, divided positive-sense single-stranded RNA genomes, and likely share common ancestors with arthropod-infecting viruses of the group negevirus and nege/kita-like viruses (Roy et al., 2015; Kondo et al., 2020; Quito-Avila et al., 2020; Ramos-González et al., 2020).

Aside from CiLV-C, the genus *Cilevirus* also includes citrus leprosis virus C2 and passion fruit green spot virus (PfGSV) (Roy et al., 2013; Ramos-González et al., 2020). The canonical cilevirus genome comprises six open reading frames (ORFs) split into two molecules, RNA1 and RNA2. RNA1 is ∼9.0 kb in length and includes two ORFs encoding the RNA-dependent RNA polymerase (*RdRp*) and the putative coat protein (*p29*). RNA2 is ∼5.0 kb in length and has four ORFs (*p15*, *p61*, *p32*, and *p24*). In CiLV-C, the RNA2 also contains an intergenic region (IR) of ∼1 kb located between the ORFs *p15* and *p61*. P15, P61, and P24 are proteins without definitively associated functions, although the first two seem to be involved in the suppression of the RNA silencing mechanism (Leastro et al., 2020) and the latter one is conserved among cileviruses, higreviruses, and an increasing number of arthropod-infecting viruses (Kuchibhatla et al., 2014; Kondo et al., 2020; Ramos-González et al., 2020). The *p32* encodes a movement protein (MP) of the 30K superfamily (Mushegian and Elena, 2015; Leastro et al., 2021).

**FIGURE 1:**
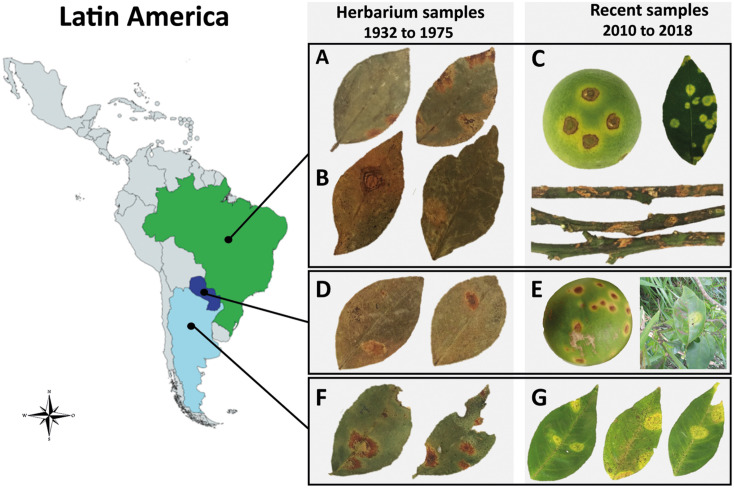
Symptoms of citrus leprosis (CL) disease and place of collection of the infected *Citrus* spp. samples across Latin America. **(A,B,D,F)** Samples conserved at the Herbarium of Instituto Biológico, São Paulo, Brazil. **(C,E,G)** Fresh citrus samples. **(A)** CiLV-C isolate Jbt02, Jaboticabal, São Paulo, Brazil, 1975. **(B)** CiLV-C isolate Urg01, Uruguaiana, Rio Grande do Sul, Brazil, 1937. **(C)** CiLV-C isolate Bar25, São Paulo, Brazil, 2018. **(D)** CiLV-C isolate Asu02 from Asunción, Paraguay, 1937. **(E)** CiLV-C isolate PY03, Paraguay, 2010 (fruit) and 2015 (leaf). **(F)** CiLV-C isolate Ar06, Misiones, Argentina, 1937. **(G)** CiLV-C isolate AR04, Corrientes, Argentina, 2017.

CiLV-C does not systemically infect its host plants, it only causes local chlorotic and/or necrotic lesions in leaves, fruits, and branches (Figure 1), which may result from an incompatible interaction led by a hypersensitivity-like response (Arena et al., 2016, 2020). The viral spread, even to different points in an infected plant, is exclusively mediated by viruliferous mites of, mainly, the species *Brevipalpus yothersi* (Ramos-González et al., 2016). *B. papayensis* is also able to transmit the virus under experimental conditions (Nunes et al., 2018). Nonetheless, the CiLV-C/*Brevipalpus* spp. interaction has not been fully characterized yet. While biological and electron microscopy data suggest a circulative transmission (Kitajima et al., 2003, 2008; Tassi et al., 2017), non-conclusive molecular assays suggested the viral multiplication in the *Brevipalpus* cells. Negative-sense CiLV-C viral genomes detected in mite extracts (Roy et al., 2015) could have been remnants from the infected plant cells after mite feeding (Tassi et al., 2017). Moreover, the specific detection of the viral negative-strand RNA by reverse transcription-polymerase chain reaction (RT-PCR) could have been the result of false amplification due to either self-priming of the positive-strand RNA or the primer activity of other cellular nucleic acids (Haddad et al., 2007; Boncristiani et al., 2009; Haist et al., 2015; Strydom and Pietersen, 2018).

Preliminary studies revealed that the CiLV-C population has a low genetic variability (π < 0.01) and is subdivided into the clades CRD and SJP (Ramos-González et al., 2016). Type viruses of each lineage share ∼85% genome nucleotide identity, except the 5′-ends of their RNA2 molecules. With ∼98% nucleotide sequence identity, the high uniformity of the genomic segments compressing the *p15-IR* regions is likely a consequence of a natural recombination process (Ramos-González et al., 2016). The lineage CRD is prevalent throughout Latin America, whereas, until 2015, the lineage SJP was only detected in three counties in the northwestern region of the state of São Paulo, Brazil (Ramos-González et al., 2016). However, most aspects concerning the diversity, distribution, transmissibility, and virulence of these strains remain largely unknown.

In this study, we investigated the distribution, dynamic and evolutionary parameters of the CiLV-C population through the analysis of 430 fresh or herbarium samples of CL-affected *Citrus* spp. tissues collected from commercial or non-commercial citrus orchards between 1932 and 2020. We also reconstructed the evolutionary history of CiLV-C and contextualized it with the origin and expansion of citrus crops in the Americas.

## Materials and Methods

### Citrus Samples

A total of 430 individual or mixed lesions from leaves, fruits, or branches were collected from 304 citrus plants showing typical chlorotic and/or necrotic symptoms of CL (Table 1 and Supplementary Table 1). The RNA extracts were obtained from: (*i*) eight sweet orange (*Citrus sinensis*) samples stored at the Herbarium of Instituto Biológico, São Paulo, collected from 1932 to 1975 in Brazil (*n* = 6), Argentina (*n* = 1), and Paraguay (*n* = 1); *ii*) 41 leaf samples of *Citrus* spp. stored in −80°C freezer collected from 2003 to 2015 in Brazil (*n* = 31), Argentina (*n* = 6), Bolivia (*n* = 1), Paraguay (*n* = 1), and Colombia (*n* = 1); (*iii*) 37 sweet orange fruit samples collected from commercial citrus orchards in the citrus belt SP-MG in the period 2015–2016; (*iv*) 18 leaf samples of *Citrus* spp. collected from non-commercial citrus orchards in Brazil (*n* = 10), Argentina (*n* = 7), and Paraguay (*n* = 1) in the period 2015–2019; (*v*) 325 fruit lesions from 199 sweet orange trees collected in 196 commercial citrus orchards in the citrus belt SP-MG in the period 2017–2020; and (*vi*) one lesion from a sweet orange fruit collected in a commercial organic orchard, State of Pará, Brazil, in 2020.

**TABLE 1:** Summary of the set of *Citrus* spp. samples gathered in this study.

Place of collection	Orchard type	Year of collection	Number of analyzed samples (individual or mixed lesions)					
			
			CiLV-C strains					Total
				
			CRD	SJP	ASU^*b*^	CDR+SJP same tree^*c*^	CDR+SJP same lesion^*d*^	
**Brazil**								
SP and MG^*a*^	Non-commercial	1932–2020	18	2	–	0	0	20
	Commercial	2003–2014	12	0	–	0	0	12
		2015–2016	7	33	–	3	–	43
		2017–2020	42	207	–	2	74	325
Other states	Non-commercial	1937–2018	10	0	–	0	0	10
	Commercial	2020	1	0	–	0	0	1
**Other countries**								
Argentina	Non-commercial	1937–2019	14	0	–	0	0	14
Colombia	Non-commercial	2008	1	0	–	0	0	1
Bolivia	Non-commercial	2003	1	0	–	0	0	1
Paraguay	Non-commercial	1937–2019	2	0	1	0	0	3
Total			108	242	1	5	74	430

*CiLV-C strains were detected by reverse transcription-polymerase chain reactions and/or high-throughput sequencing.*

*^a^Brazilian states abbreviations: São Paulo (SP) and Minas Gerais (MG).*

*^b^Identification by HTS, giving that all available primers were not able to identify this strain.*

*^c^Members of the clades SJP and CRD detected in samples from the same tree.*

*^d^Members of the clades SJP and CRD detected in the same CL lesion.*

### RNA Isolation

RNA extraction was performed either from fresh or herbarium plant tissues. For fresh samples, about 100 mg of leaf lesions were ground in liquid nitrogen and the total RNA was extracted using Trizol^®^ according to the manufacturer’s recommendation (Thermo Fisher Scientific, Waltham, MA, United States). For the herbarium samples, in addition to the treatment with 0.01% diethylpyrocarbonate (DEPC) solution and 120°C sterilization, mortars and pestles were kept in an oven at 200°C for 48 h before the extractions. Approximately 600 mg of dry symptomatic tissues were ground in liquid nitrogen and processed following the Trizols^®^ procedure modified as previously described (Ramos-González et al., 2016). Regardless of the origin of samples, final RNA solutions were precipitated using 0.1 volume of sodium acetate 3 M and 2.5 volume of isopropanol, kept at −80°C for 12 h, and centrifuged at 10,000 × g for 10 min at 4°C. The concentration and quality of the RNA extracts were assessed by NanoDrop ND8000 spectrophotometer (Thermo Fisher Scientific), and 1.2% agarose gel stained with ethidium bromide (10 mg/mL) or Bioanalyzer 2100 (Agilent Technologies, Santa Clara, United States), respectively. For samples collected in commercial citrus orchards from 2017 to 2020, the total RNA extracts were obtained from a single lesion found on the affected fruits. With this, we aimed to reduce the interference resulting from a putative intra-host virus variability and to detect whether viruses belonging to more than a clade could be infecting a single lesion (detailed in Supplementary Table 1).

### Detection of CiLV-C and Other Citrus Leprosis Symptom Producing Viruses by RT-PCR

Five hundred nanograms of total RNA were used for cDNA synthesis in a final reaction volume of 20 μL using the RevertAid H Minus First-Strand cDNA Synthesis Kit (Thermo Fisher Scientific). The presence of CL-associated viruses was assessed by PCR using cDNA as template (3 μL), specific primer pairs (Table 2), and GoTaq G2 Master Mix Green kit (Promega, Madison, WI, United States). For CiLV-C, in addition to primers for the detection of *p29* (Ramos-González et al., 2016) and *p32* (Locali et al., 2003), a set of strain-specific and degenerate primers were developed based on available GenBank *p24* sequences (CiLV-C RNA2 of the isolates SJP01 and Crd01: KP336747 and NC008170, respectively). To do this, sequences were aligned using MUSCLE implemented in MEGA version 7.0.21 (Kumar et al., 2016), and primers were designed using Geneious software platform version 11.1.4 (Kearse et al., 2012) (Table 2). The thermal cycles were as follows: 94°C, 3 min; 35 cycles of 94°C, 30 s; 54°C, 30 s; 72°C, 30 s; and a final extension at 72°C for 5 min. To confirm the specificity of primers, the amplicons were resolved on a 1% agarose gel, excised, purified, and Sanger sequenced. The putative presence of the cilevirus citrus leprosis virus C2 (CiLV-C2) (Roy et al., 2013) and the dichorhaviruses citrus leprosis virus N (CiLV-N) (Ramos-González et al., 2017), citrus chlorotic spot virus (CiCSV) (Chabi-Jesus et al., 2018), and orchid fleck virus (OFV) (Kubo et al., 2009) were screened by PCR using previously described primers (Table 2).

**TABLE 2:** Primer list used for the detection of viruses associated with citrus leprosis disease by reverse transcription-polymerase chain reaction.

Virus^*a*^	Target	Primer sequence (5′–3′)	Ta^*b*^ (°C)	Amplicon size (bp)	References
CiLV-C	*p32*	F: GCGTATTGGCGTT GGATTTCTGAC	56	339	Locali et al., 2003
		R: TGTATACCAAGCC GCCTGTGAACT			
	*p29*	F: ACCGTGAATTTGT ATTTTGTCA		1,000	Ramos-González et al., 2016
		R: CAGCTGGAAGAGA CTAGAAA			
	*p15*	F: GTCAAGTGATATCC ATTTTGCTTG		667	Ramos-González et al., 2016
		R: TCATCGTCTTTTC TGTAACCG			
	*p24*	F: CGCAGTTTCCTAA TAACACC	54	322	This study
		R: GCTTTATGCTGAA CTCCC			
CiLV-C CRD	*p29*	F: CAGAAGGCCGAGG TTGTAAAG	56	330	Ramos-González et al., 2016
		R: GTAGTGATCACT GAACTCGAATACC			
	*p24*	F: ATGTTGGCAACG GAAAGTT	54	522	This study
		R: GTGAACAGGGTTG AAAAAGTT			
CiLV-C SJP	*p29*	F: GTAARCAAAAGG TCGAGGTTGTCC	56	456	Ramos-González et al., 2016
		R: TCTGTTGTCTAGC AGCRAGTAATG			
	*p24*	F: CTCATGATATCCTTG ATGACC	54	393	This study
		R: GACTAATAAGGTT GAGAAGGTTG			
CiLV-C2	*p29*	F: ATGAGTAACATTG TGTCGTTTTCGTTGT	56	795	Roy et al., 2013
		R: TCACTCTTCCTGTT CATCAACCTGTT			
OFV +CiLV-N	*L*	F: CAASTGTCATGCC TGCATGG	54	362	Ramos-González et al., 2017
		R: TTGATRCATGATG CRAGRCTGTATG			
CiCSV	*G*	F: CTGTTTTGCCCAT GCTAC		500	Chabi-Jesus et al., 2018
		R: CCTCCTCTTCTAG CGTCAT			

*^a^Virus or clade-specific viruses identified by each primer pair. Virus acronyms: CiLV-C, citrus leprosis virus C; CiLV-C CRD, lineage CRD; CiLV-C SJP, lineage SJP; CiLV-C2, citrus leprosis virus C2; OFV, orchid fleck virus; CiLV-N, citrus leprosis virus N; CiCSV, citrus chlorotic spot virus.*

*^b^Ta: PCR annealing temperature. F and R indicate forward and reverse primers, respectively.*

### Partial Sequencing of CiLV-C Isolates

The complete sequence of *p29* (795 nts) and partial sequence of *p32* (288 nts) in the RNA1 and RNA2, respectively, of CiLV-C isolates, were obtained using described primers (Locali et al., 2003; Ramos-González et al., 2016; Table 2). Amplicons were obtained from 26 samples collected in non-commercial citrus regions in Brazil and Argentina, from 2006 to 2019, and 31 from commercial citrus orchards inside the citrus belt SP-MG, in the period 2017 to 2019 (Supplementary Table 1). After RT-PCR, amplicons were purified using Wizard SV Gel and PCR Clean-Up System (Promega, Madison, WI, United States), and cloned into pGEM-T-Easy (Promega, Madison, WI, United States). Plasmids were transformed into *Escherichia coli* DH10β competent cells by electroporation, and 5–10 recombinant clones derived from each sample were sequenced by the Sanger method (Instituto Biológico, SP, Brazil). PCR products from some samples collected in non-commercial orchards (Supplementary Table 1) were directly sequenced after the purification using the Wizard SV Gel and PCR Clean-Up System (Promega).

### High-Throughput Sequencing of CiLV-C Genomes

Small RNA (sRNA) from the herbarium samples were sequenced on an Illumina HiSeq 2500 system (Illumina, San Diego, United States) either at Genewiz (South Plainfield, NJ, United States) or BGI (Shenzhen, Guangdong, China) (Table 3). For most of the non-herbarium samples, total RNA extracts were processed at the Laboratory of Animal Biotechnology of the University of São Paulo (Piracicaba, SP, Brazil). Poly(A) enrichment of the RNA extracts and cDNA libraries were prepared with Illumina TruSeq Stranded mRNA Library Prep Kit (Illumina, San Diego, United States). Sequencing was performed in an Illumina HiSeq 2500 system using HiSeq SBS v4 High Output Kit (Illumina, San Diego, United States). Paired-end reads of 2 × 125 bp were generated. The viral sequence in the sample BR_SP_Jmr01 was obtained using the Ion GeneStudio^TM^ S5 System (Thermo Fisher Scientific) at the Instituto Biológico, São Paulo, Brazil. The sRNA library from this sample was obtained using Ion Total RNA-Seq Kit v2 (Thermo Fisher Scientific). The quality of reads obtained by all the methodologies was checked using FastQC (Andrews, 2010) and the adaptor sequences were removed using the Trimmomatic (Bolger et al., 2014). Reads from all types of libraries were assembled with SPAdes (Bankevich et al., 2012) although using different *k*-mer sizes: 15, 17, 19 for sRNA libraries, and 33, 43, 55 for poly(A)-enriched RNA libraries. Viral contigs were identified using the Basic Local Alignment Search Tool (BLASTx and/or BLASTn) implemented in Geneious using a local database including viral reference genomes retrieved from the NCBI virus database^2^ (Hatcher et al., 2017). After the identification and when necessary, reads were mapped to the reference genomes in an iterative mapping approach (Tsai et al., 2010) using Bowtie2 or BBMap to fill gaps and extend the end sequences of viral genomes (Langmead and Salzberg, 2012; Bushnell, 2014).

**TABLE 3:** Brief description of the 18 high-throughput sequencing (HTS) libraries obtained in this study.

Host species/variety	Local of collection city/state/country	Year of collection	Sequencing company	HTS library	Number of reads	RNA molecule	CiLV-C-derived reads and contigs			
							
							% of viral-derived reads in the library	Number of assembled contigs	Contig length range (nts)	Viral coverage (%)
**Herbarium samples**										
*Citrus sinensis*	Jacarei, SP, BR^2^	1932	Genewiz, United States	siRNA	59,435,658	1	0.01	15	150–1423	99.3
						2	0.01	9	148–2116	99.6
*C. sinensis* (Washington Navel)	Piracicaba, SP, BR	1932	Genewiz, United States	siRNA	71,037,882	1	0.01	6	121–4168	100
						2	0.01	3	1478–1916	100
*C. sinensis*	Uruguaiana, RS, BR	1937	BGI, China	siRNA	63,116,147	1	2.1	3	235–7026	100
						2	1.1	2	24499–2317	100
*C. sinensis* (Washington Navel)	Asuncion, PY	1937	BGI, China	siRNA	61,507,832	1	20.2	6	403–3709	100
						2	14.6	4	1085–3781	100
*C. sinensis*	Santa’Ana, Misiones, AR	1937	BGI, China	siRNA	62,116,588	1	6.5	2	427–8144	100
						2	4.3	1	4785	100
*C. sinensis* (Washington Navel)	Limeira, SP, BR	1939	Genewiz, United States	siRNA	74,407,978	1	0.02	6	187–7577	100
						2	0.01	4	4850–4858	100
*C. sinensis* (Pera)	Sao Paulo, SP, BR	1941	BGI, China	siRNA	59,404,138	1	0.05	19	147–988	100
						2	0.05	9	144–707	100
*Citrus* sp.	Jaboticabal, SP, BR	1975	Genewiz, United States	siRNA	56,790,128	1	16.4	22	117–4750	100
						2	7.7	19	125–4428	100
**Sample stored at −80°C**										
*C. sinensis*	AR	2006	Esalq, USP, Brazil	mRNA	14,872,149	1	14.2	23	154–8724	100
						2	2.5	5	162–5276	100
**Samples from fresh tissues**										
*C. sinensis*	Piracicaba, SP, BR	2016	Esalq, USP, Brazil	mRNA	15,553,431	1	5.5	2	630–8147	100
						2	1.2	1	4963	100
*C. sinensis*	Corrientes, AR	2017	Esalq, USP, Brazil	mRNA	14,410,186	1	1.6	1	8747	100
						2	1.4	2	292–4742	100
*C. reticulata*	Piracicaba, SP, BR	2018	Esalq, USP, Brazil	mRNA	15,165,394	1	0.9	3	416–8755	100
						2	0.2	1	4975	100
*C. sinensis*	Sud Mennucci, SP, BR	2018	Esalq, USP, Brazil	mRNA	16,377,921	1	20.6	32	93–821	100
						2	6.8	30	58–892	100
*C. sinensis*	Vitoria, ES, BR	2018	Esalq, USP, Brazil	mRNA	13,111,075	1	6.7	8	181–7800	100
						2	1.6	1	4968	100
*C. sinensis*	Capitão Poço, PA, BR	2020	Esalq, USP, Brazil	mRNA	15,822,801	1	3.8	6	151–7781	100
						2	2.2	7	218–2345	100
*C. sinensis*	Limeira, SP, BR	2020	Esalq, USP, Brazil	mRNA	15,390,982	1	26.9	23	69–4377	100
						2	11.5	34	104–1031	100
*C. reticulata*	Jumirim, SP, BR	2020	Instituto Biológico, SP, Brazil	siRNA	10,557,136	1	13.3	8	250–3340	100
						2	6.7	4	470–1428	100
*C. sinensis*	Santo Antônio da Posse, SP, BR	2020	Esalq, USP, Brazil	mRNA	16,571,118	1	9.9	27	82–7820	100
						2	6.5	24	58–892	100

*^*a*^Country: BR, Brazil; AR, Argentina; PY, Paraguay.*

### Recombination and Reassortment Analyses

Recombination events were assessed using seven methods (RDP, GENECONV, Bootscan, Maxchi, Chimaera, SiScan, and Topal) implemented in RDP version 5.5 (Martin et al., 2015) and GARD (Kosakovsky Pond et al., 2006). Sequences were aligned using the MUSCLE, MAFFT, and Clustal software, implemented in Geneious. Recombination events detected by more than three programs (*p* ≤ 0.05) implemented in the RDP vs. 5.5 were considered as recombinants. Due to the length heterogeneity of sequences available, four independent analyses were carried out: (*i*) complete sequences of each CiLV-C genome (8,984 nts of RNA1 and 5,077 nts of RNA2; *n* = 23); (*ii*) *p29* (RNA1) (795 nts; *n* = 190); (*iii)* partial *p32* (RNA2) (288 nts; *n* = 270); (*iv*) and a partial RNA2 concatenated sequences [complete *p15* ORF (393 nts)—intergenic region (934 nts, upstream the *p61* ORF)—partial *p32* (288 nts); *n* = 56]. The reassortment events were analyzed based on the topology of the phylogenetic trees. All CiLV-C sequences available at the GenBank were retrieved and incorporated into this and further *in silico* analyses (Supplementary Table 1).

### Phylogenetic Analyses Based on Complete Genomes and CiLV-C ORFs

Nucleotide sequence alignments were performed using MUSCLE implemented in MEGA version 7.0.21 (Kumar et al., 2016). Best-fit models for nucleotide substitutions were determined with Bayesian Information Criterion (BIC) implemented in MEGA version 7.0.21 (Kumar et al., 2016). They were as follow: model GTR+G for RNA1 (*n* = 23), HKY+G+I for both the RNA2 (*n* = 23) and the concatenated partial RNA2 sequences (*p15*-IR-*p32*, *n* = 56), and HKY+G for the nucleotide sequences alignments of *p29* (*n* = 190) and *p32* (*n* = 270). Phylogenetic trees were generated by Bayesian inference using a variant of Markov chain Monte Carlo (MCMC) with MrBayes, implemented in Geneious (Huelsenbeck and Ronquist, 2001; Kearse et al., 2012), with 6,000,000 generations and cognate sequences from CiLV-C2_Colombia (NC038848 and NC038849) as outgroup. Genomic regions involved in the recombination events were excluded before the phylogenetic tree building to minimize their influence on tree topologies. Trees were viewed and edited using iTOL version 4 (Letunic and Bork, 2019). Nucleotide distances within and between clades were calculated using MEGA version 7.0.21.

### Temporal Phylogenetic Analyses of the CiLV-C Population

Assessment of the time to the most recent common ancestor (tMRCA) of CiLV-C isolates was carried out using BEAST software version 1.10.04 (Suchard et al., 2018). Two datasets were evaluated. They comprised (*i*) the concatenated sequences of all CiLV-C ORFs (except the *p15* of RNA2 because its putative recombinant origin) (11,473 nts; *n* = 23 isolates) and (*ii*) concatenated sequences of the complete *p29* and the partial *p32* ORFs (1,083 nts, *n* = 132 isolates). In samples from which more than one haplotype was sequenced, only those showing divergent sequences were included in the analyses (Supplementary Table 2), but nucleotide diversity inside a given sample was always lower than 0.007. “Non-clock” maximum likelihood phylogenetic trees were reconstructed with the best evolutionary model (TN93+G) using IQtree software version 1.5.5 (Nguyen et al., 2015), and the temporal signal was evaluated by TempEst.

To assess the evolutionary history of the CiLV-C population, the Bayesian Markov Chain Monte Carlo (MCMC) was estimated using the BEAST version 1.10.4 The best model of nucleotide substitution for the two analyzed datasets was TN93+G. The Bayesian skygrid model (number of parameters = 20; time of last transition point = 88) was selected as the tree coalescent model and using the strict clock. The MCMC analyses were performed with 100 million generations, sampling a tree every 1,000 steps. MCMC convergence was assessed by estimating the effective sample sizes (ESS) using Tracer version 1.7 (Rambaut et al., 2018). ESS > 100 are moderate values whilst values > 200 are considered better, according to the instruction manual of the software^3^. The maximum clade credibility (MCC) tree was created by discarding the initial 10% of the chains and summarized in TreeAnnotator version 1.10.4. The phylogenetic tree was viewed and edited using IcyTree (Vaughan, 2017).

### Population Genetics and Selection Tests

Population genetic parameters, i.e., diversity of nucleotide (π) and haplotype (Hd), the number of polymorphic sites (s), nucleotide differences (k), average mutation rates (θ), and haplotypes (H); and the ratio of non-synonymous (dN) to synonymous (dS) nucleotide substitutions (ω = dN/dS) were calculated using DnaSP v. 6.12.03 (Rozas et al., 2017). Selection in polymorphic sites of the CiLV-C ORFs was calculated using Fast Unconstrained Bayesian AppRoximation for Inferring Selection (FUBAR), Fixed Effects Likelihood (FEL), and Mixed Effects Model of Evolution (MEME) methods with the GTR model, implemented in Datamonkey 2.0 (Weaver et al., 2018). For the identification of the amino acids under selection and their involvement in the protein structure, predicted secondary structures of deduced amino acid sequences of the P29 and MP proteins from definitive and tentative members of the genus *Cilevirus*: CiLV-C Crd01 (NC008169 and NC008170), CiLV-C_SJP01 (KP336746 and KP336747), CiLV-C2_Colombia (NC038848 and NC038849), hibiscus strain of CiLV-C2_Hawaii (MG253805 and MG253804) and PfGSV_Snp1 (MK804171 and MK804172) were obtained using PROMALS (PROfile Multiple Alignment with Local Structure) (Pei and Grishin, 2007).

### Tests of Selective Neutrality and Differentiation in the CiLV-C Population

CiLV-C population expansion was evaluated by the statistical tests Tajima’s D (Tajima, 1989), Fu and Li’s F and D (Fu and Li, 1993), and Fu’s FS (Fu, 1997), implemented in DnaSP v. 6.12.03 package (Rozas et al., 2017). They estimated the difference between two measures of genetic diversity, i.e., the mean number of pairwise differences and the number of segregating sites. In these tests, negative values denote populations in expansion or after a recent bottleneck, whereas positive values mean a decrease in population size and/or balancing selection.

Demographic expansions of CiLV-C subpopulations assessed by mismatch distributions (distribution of pairwise nucleotide differences) were performed based on the sum of squared deviation (SSD) and Harpending’s Raggedness index (HRI) using Arlequin v. 3.5.2.2 (Excoffier and Lischer, 2010). The HRI test determines whether an observed mismatch distribution is drawn from an expanded (small raggedness index or non-significative) or a stationary population (large raggedness index), while the SSD quantifies the smoothness of the observed mismatch distribution and a non-significant result indicates an expanding population (Rogers and Harpending, 1992; Harpend, 1994).

Genetic subdivision of the CiLV-C population was assessed using the following tests implemented in DnaSP and Arlequin v. 3.5.2.2: the nearest-neighbor statistic (*Snn*) (Hudson, 2000), Hudson’s test statistics [*Hst* (haplotype-based statistics), *Kst* (nucleotide-based statistics)], Wright’s fixation index (*Fst*), and gene flow (*Nm*) (Hudson et al., 1992). The *Snn* is a measure of how often the nearest neighbors of sequences are found in the same locality (Hudson, 2000). *Snn* values range from 0.5 to 1, being the lowest indexes a sign that isolates from both locations are part of the same population and the highest ones that the populations in the two locations are highly differentiated. *Hst* and *Kst* statistics calculate the level of differentiation based on haplotypes and nucleotides, respectively, and values close to zero mean no differentiation. On the other hand, based on the proportion of the total genetic variance contained in a subpopulation, the *Fst* test provides insights into the evolutionary processes that influence the structure of genetic variation within and among populations (Hudson et al., 1992). *Nm*, the number of migrants successfully entering a population per generation, was used to measure gene flow (migration) between populations [FST≠1/(4*Nm*+1)]. Besides, the partitioning of variation at different levels was calculated by Analysis of Molecular Variance (AMOVA) in Arlequin using 1,000 permutations.

## Results

The presence of CiLV-C was confirmed in all the 430 symptomatic samples collected from 304 plants of sweet orange, six of mandarin, and in other five citrus plants whose species could not be determined (Supplementary Table 1). RT-PCR tests for the specific detection of citrus-infecting *Brevipalpus*-transmitted viruses other than CiLV-C indicated the absence of the cilevirus CiLV-C2 and the dichorhaviruses CiLV-N, CiCSV, and OFV (Table 1). Overall, this study included CiLV-C isolates collected from Argentina, Brazil, Bolivia, Colombia, and Paraguay during the period 1932–2020. Based on the high production volume and the large size of the farming area, ∼92% of the samples were collected from the citrus belt SP-MG, Brazil. All the analyzed samples showed typical symptoms of citrus leprosis disease, i.e., chlorotic and/or necrotic lesions on leaves and fruits and necrotic lesions on branches (Figure 1).

### Near-Complete Genome Sequencing of New CiLV-C Isolates Reveals a Novel Divergent Strain

Total RNA extracts of leaves from eight herbarium, nine fresh, and one −80°C frozen-conserved samples were obtained (Table 3). RNA integrity number (RIN) of the extracts prepared from the herbarium samples was low, i.e., 1.6–2.1. Despite this, *de novo* assembling of the raw reads using the SPAdes enabled the recovery of more than 80–90% of the CiLV-C genomes from the high-throughput sequencing (HTS) libraries. Particularly, from the sample of sweet orange collected in 1941, in SP, Brazil, few and shorter contigs were obtained and only ∼60% of the CiLV-C genome could be determined. In this case, gaps between contiguous contigs were filled after a new round of assembling using BBMap and the CiLV-C genome as a reference. In sum, approaches combining *de novo* assemblies and the iterative mapping increased the genome coverages by about 100% in several samples. The lower genome coverage observed during the initial assembly steps of some herbarium samples seemed independent of the collection year and the laboratory where the HTS libraries were processed. Rather, it appeared to be intrinsically associated with the conservation procedure of every single sample, as previously observed (Hartung et al., 2015). Recovery rates of viral genomes higher than 98% were obtained from all fresh or −80°C conserved samples using the same wet lab and *in silico* procedures and tools. Overall, the complete or near-complete genomes of 18 studied isolates of CiLV-C were obtained.

**FIGURE 2:**
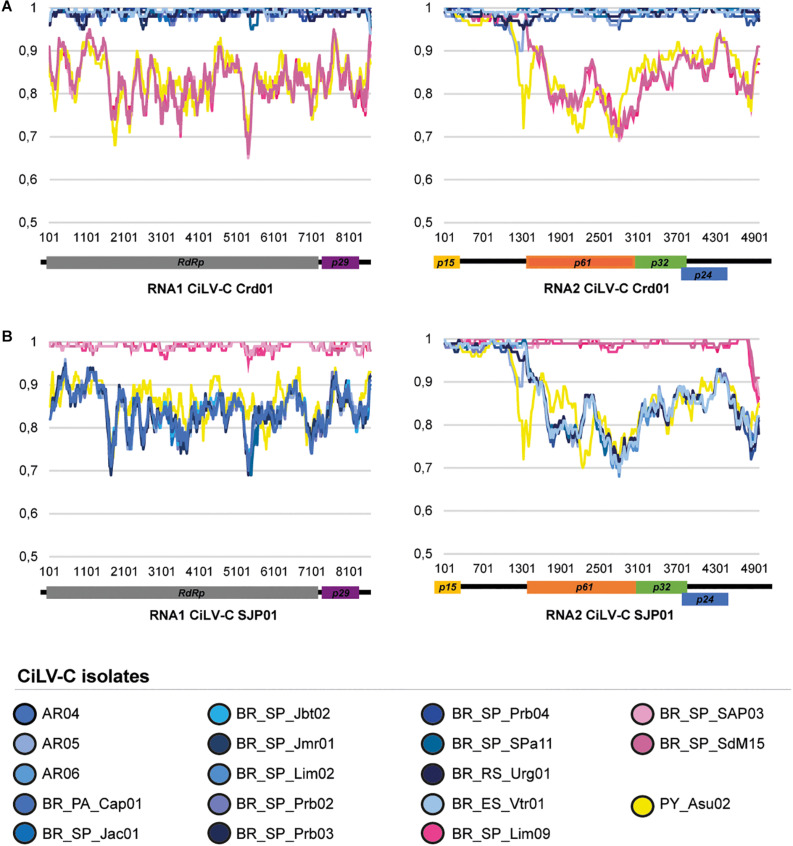
Similarity plots of the nucleotide sequences of the RNA1 and RNA2 of the CiLV-C isolates. The full-length genome sequences of the 18 studied CiLV-C isolates were compared to those of the type-member viruses of the clades CRD **(A)** and SJP **(B)**. Curves depict the comparison between the analyzed and a reference genome. Each plotted point is the percent identity (vertical axis) within a sliding window 200 bp wide centered on the position plotted, with a step size between points of 20 bp. The horizontal axis indicates the nucleotide positions across the RNA1 and RNA2 molecules of the reference genomes. The horizontal bars above the curves are a cartoon of the ORFs of the CiLV-C genome. Plots were generated using SimPlot (Lole et al., 1999).

The pairwise comparison of the genome sequences of the studied HTS CiLV-C isolates with those of the type viruses of the clades CRD (isolate Crd01, NC008169 and NC008170) and SJP (isolate SJP01, KP336746, and KP336747) showed values of nucleotide sequence identity that ranged from 84.0 to 99.8% and separates them into three groups (Figure 2 and Supplementary Table 3). Fourteen isolates showed the highest identity values with the reference sequence of the clade CRD, three with that of the clade SJP, while the isolate CiLV-C_PY_Asu02, collected in Asunción, Paraguay in 1937, typified a novel diversity of CiLV-C and shared less than 86% nucleotide sequence identity with the reference genomes.

CiLV-C_PY_Asu02 shows the same genomic organization of the type member of the genus *Cilevirus*. The profile of nucleotide sequence identity across the genomes of the isolates PY_Asu02 and Crd01 follows the same pattern observed in the comparison between the type viruses of the clades SJP and CRD (Figure 2). Deduced amino acid sequences of ORFs from CiLV-C_PY_Asu02 and CiLV-C Crd01 show pairwise identity values ranging from 81% for P61, to 100% for P15, which resembles what is observed in the comparison among proteins from viruses of the clades SJP and CRD (Table 4). Notably, the stretch of nucleotide sequences at the 5′-end of the RNA2 in CiLV-C_PY_Asu02, which includes the ORF *p15* and part of the IR, is highly conserved between the isolates of the three clades, suggesting a common origin. CiLV-C_PY_Asu02 shows percentages of nucleotide sequence identity lower than 50% with the cileviruses CiLV-C2 and PfGSV (Table 4), confirming that the isolate PY_Asu02 represents a viral diversity previously unknown among members of the species *Citrus leprosis virus C*.

**TABLE 4:** Nucleotide (nt) and deduced amino acid (aa) sequence identities between CiLV-C_PY_Asu02 and other members of the genus *Cilevirus.*

CiLV-C PY_Asu02^*a*^	CiLV-C Crd01		CiLV-C SJP01		CiLV-C2_Co		CiLV-C2_Hw		PfGSV_Snp1	
					
	nt	aa	nt	aa	nt	aa	nt	aa	nt	aa
**RNA1**	**86.2**	–	85.1	–	57.2	–	58.0	–	57.1	–
*RdRp*	86.3	93.4	**88.2**	**94.9**	59.6	58.86	59.8	58.7	59.2	57.9
*p29*	**86.0**	**94.0**	85.6	89.8	43.7	34.54	45.5	33.9	44.4	32.6
**RNA2**	**85.8**	–	85.1	–	44.3	–	43.3	–	43.4	–
*p15*	**98.5**	**100**	**98.5**	**100**	28.5	19.61	34.9	20.4	27.7	19.2
IR	**85.0**	–	84.0	–	29.1	–	31.4	–	26.9	–
*p61*	82.1	81.1	**82.9**	**83.8**	44.7	31.72	43.4	33.9	45.3	32.6
*p32*	**89.3**	**95.0**	86.5	94.0	55.3	51.16	54.9	50.5	52.5	53.9
*p24*	88.6	94.0	**89.2**	**94.4**	60.9	60.63	60.3	59.7	60.9	60.4

*The highest values are highlighted in bold.*

*^a^GenBank accession numbers of each isolate: CiLV-C_PY_Asu02: MT554532 (RNA1) and MT554546 (RNA2); CiLV-C Crd01: NC008169 and (RNA1) and NC008170 (RNA2); CiLV-C SJP01: KP336746 (RNA1) and KP336747 (RNA2); CiLV-C2_Co: NC038848 (RNA1) and NC038849 (RNA2); CiLV-C2_Hw: MG253805 (RNA1) and MG253804 (RNA2); and PfGSV_Snp1: MK804171 (RNA1) and MK804172 (RNA2).*

### RNA2 of CiLV-C Strains Harbor Signals of Recombination

Putative signals of recombination events were detected in the RNA2 of CiLV-C using two datasets comprising the complete (*n* = 23) and partial (*n* = 56) sequences of the molecule. Partial RNA2 molecules contained the concatenate sequences of *p15*-IR-*p32*.

**FIGURE 3:**
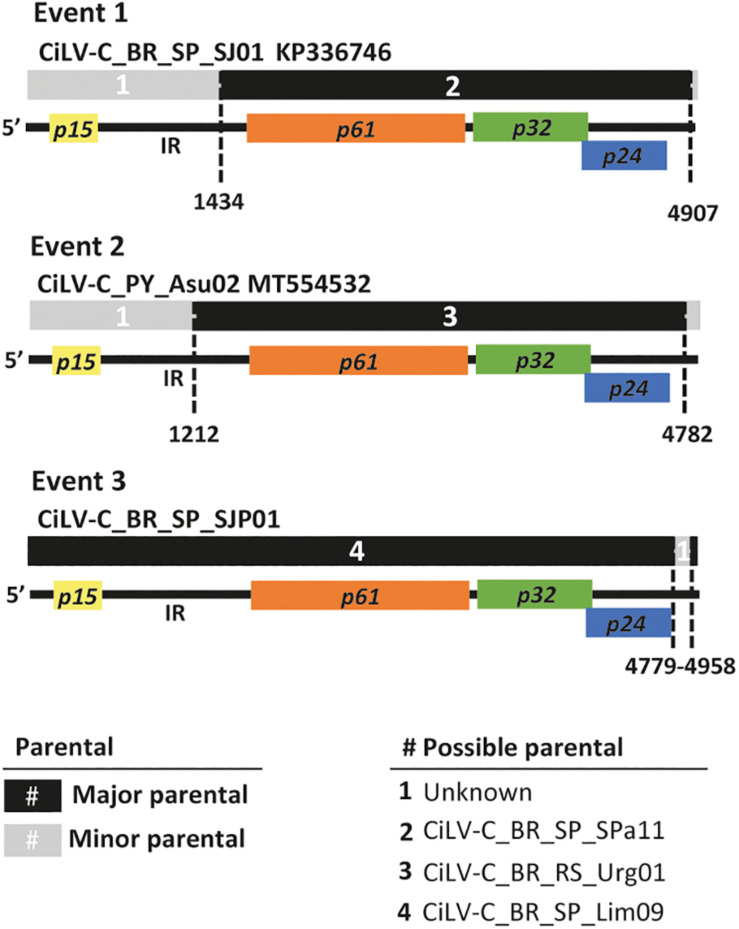
Evidence of recombination in the RNA2 of CiLV-C. The diagram depicts three recombination events detected using the alignment of the completed RNA2 molecules of 23 isolates. Multicolored horizontal bars represent the genome of the recombinant molecules. Major and minor parentals are indicated by black and gray bars and numbers (#) as described in the legend. Dashed vertical lines indicate the recombination breakpoints using a cartoon representing the RNA2 molecule of CiLV-C as reference. Event 1 shows the recombination found in all isolates of the clade SJP, event 2 was detected in the isolate PY_Asu02 (clade ASU), and event 3 in BR_SP_SJP01 (clade SJP). Recombination events were detected by more than four programs implemented in the software RDP. Details of these and other recombination events are shown in Supplementary Table 4.

In the analyses of the complete RNA2 molecules, at least four out of seven programs implemented in the RDP version 5.5 identified recombinant events involving *p15* and the intergenic region (IR) (Figure 3 and Supplementary Table 4). For instance, in the isolates BR_SP_SJP01, BR_SP_SJP05, BR_SP_Lim09, BR_SP_SAP03, and BR_SP_SdM15 of the clade SJP, as well as in the isolate CiLV-C_PY_Asu02, the seven programs suggested two breakpoints, one inside the IR and the second one closer to the 3′-end of their molecules. In both cases, isolates from the clade CRD (BR_SP_SPa11 or BR_RS_Urg01) were detected as the minor parents while the major parents were indeterminates. The third event, in the 3′-end of the RNA2 of CiLV-C_BR_SP_SJP01, involves CiLV-C_BR_SP_Lim09 as the major parent and an unknown minor parent.

When the alignments of the concatenated sequences were used, despite the artificial organization of the sequences, four events could be detected. The first two were identified in the *p15* and the IR of thirteen isolates belonging to the clade SJP, with CiLV-C_BR_PA_Bel01 as the minor parent and unknown major parents; the third event was detected within the IR of the isolate CiLV-C_BR_PR_Mgf01, with the isolate CiLV-C_AR05 as the minor parent and an unknown major parent. Finally, the fourth event, detected in the isolate PY_Asu02, has breakpoints in the IR and the beginning of *p32*, and the isolate BR_PR_Ldb01 was indicated as the minor parent while the major parent could not be identified (Supplementary Table 4). Recombination events using separately either the partial sequences of the ORF *p32* (*n* = 270) and those of the complete ORF *p29* (*n* = 190) were not detected.

### Phylogenetic and Genetic Analyses Support the Existence of Three Distinct Clades of CiLV-C: CRD, SJP, and ASU

Datasets grouping CiLV-C sequences generated in this work and those retrieved from GenBank were used for phylogenetic analyses. They comprised, in total, 23 complete or near-complete genomes, 167 complete *p29* sequences, and 247 partial *p32* sequences from isolates collected in six countries across Latin America in the period 1932–2020.

**FIGURE 4:**
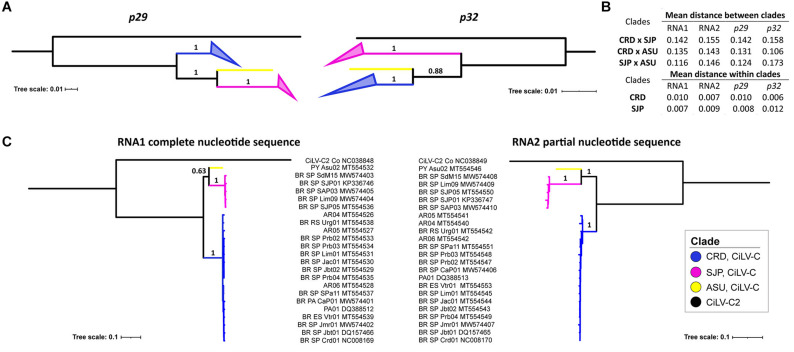
Phylogenetic relationships and genetic distances among CiLV-C strains. Phylogenetic trees were generated by Bayesian inference using MrBayes and based on *p29* (190 isolates, 795 nts) and *p32* (270 isolates, 288 nts, partial) **(A)**, and the complete sequences of the RNA1 molecules and partial sequences of the RNA2 molecules (*p61*, *p32*, and *p24*) of 23 isolates of CiLV-C **(B)**. Inter- and intra-clade nucleotide distances were calculated considering independent ORFs and complete genomic molecules **(C)**. The maximum clade credibility trees were generated with 6,000,000 generations and using the appropriate sequences of CiLV-C2_Colombia (NC038848 and NC038849) as an outgroup. Tree branches were identified according to CiLV-C lineages.

Bayesian phylogenetic reconstructions using the data sets of *p29* and *p32* showed three major branches, where the two largest ones encompassed the isolates of the previously identified lineages CRD and SJP (Figure 4A and Supplementary Figure 1). The third branch, supported with a high value of posterior probability (0.88) and hereafter called the lineage ASU, included only the isolate CiLV-C_PY_Asu02, collected in Asunción, Paraguay, in 1937. The subdivision in three clades of the CiLV-C population was also supported by the analysis of intra- and inter- clades genetic distances. Using both *p29* and *p32*, inter-clade genetic distances (0.106 ≤ *d*_*inter*__–__*clade*_ ≤ 0.173) were, generally, 11-fold higher than the intraclade distances (*d*_*intra*__–__*clade*_ ≤ 0.01) (Figure 4B). Moreover, trees constructed with the complete genome sequences showed the same clade topology observed in the analyses using the independent ORFs (Figure 4C). It is noteworthy that for the construction of the RNA2 tree, the first 1,434 nts of the 5′-end of each molecule were removed considering the putative origin by recombination of this genomic region. Reassortment events between isolates from either different or the same clade were not observed.

Nucleotide (π) and haplotype diversity (Hd) values intrinsic to the CiLV-C population were calculated based on ORFs *p29* and *p32* (partial sequence) (Table 5). Although the nucleotide diversity of the whole population (SJP+CRD+ASU) was relatively very low (π∼0.07), its value was roughly 10-fold higher than those observed for the independent clades SJP and CRD (π∼0.006–0.01), and the pattern was similar regardless of the analyzed ORF, i.e., *p29* or *p32*. The haplotype diversity was close to 1 in any of the analyzed groups.

**TABLE 5:** Population genetics parameters and assessment of selection pressure for ORF *p29* (795 nts) and *p32* (288 nts, partial sequence) of CiLV-C.

ORF	Dataset (number of isolates)	H	Hd	π	ω (dN/dS)
***p29***	SJP+CRD+ASU (190)	159	0.9975	0.07519	0.278
	SJP+CRD (189)	158	0.9975	0.07501	0.294
	SJP (106)	87	0.995	0.00803	0.359
	CRD (83)	71	0.995	0.00994	0.448
***p32***	SJP+CRD+ASU (270)	70	0.930	0.07284	0.137
	SJP+CRD (269)	69	0.929	0.07224	0.165
	SJP (190)	49	0.917	0.01010	0.362
	CRD (80)	27	0.777	0.00631	0.145

*Independent analyses were carried out with different combinations of viral isolates according to phylogenetic clades.*

*H, number of haplotypes; Hd, haplotype diversity; π, nucleotide diversity; and ω = dN/dS, ratio of non-synonymous (dN) to synonymous (dS) substitutions.*

### Viruses of the Clade CRD Are Spread Across the Continent Whereas Those of the Clade SJP Are Restricted to the Brazilian Citrus Belt SP-MG

**FIGURE 5:**
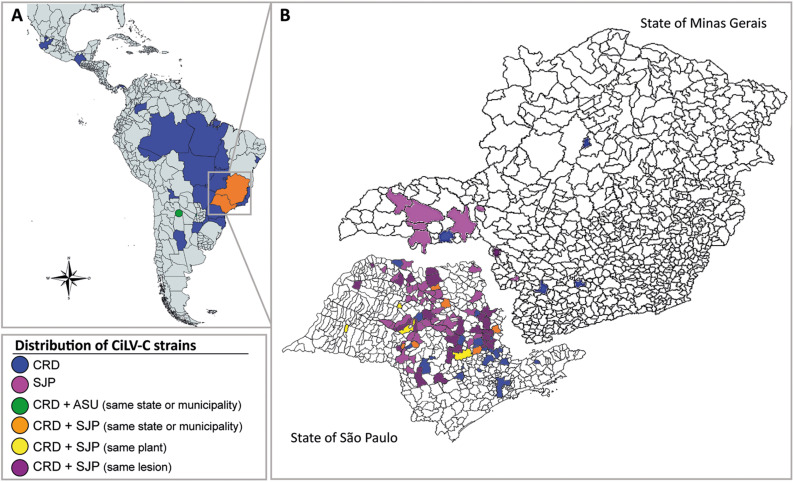
Distribution of CiLV-C strains in Latin America **(A)** and across municipalities in the Brazilian states of São Paulo and Minas Gerais **(B)** in the period from 1932 to 2020. Viral detection was based on RT-PCR assays for specific identification of *p29* and *p24* from isolates of the CRD and SJP clade. CiLV-C_ASU was identified by high-throughput sequencing. The color-coded legend indicates the presence of a viral isolate, concomitantly or at a different time in the same plant, in single or mixed infections. The map also depicts the distribution of CiLV-C isolates whose sequences were already available in the GenBank database.

Spatial and temporal distribution of isolates of the clades CRD and SJP were assessed by RT-PCR using clade-specific primers for the detection of *p29* (RNA1) and *p24* (RNA2) sequences. Results were screened for the spatial and temporal distribution of members of each clade (Table 1, Figure 5, and Supplementary Table 1). Unfortunately, the genomic characterization of the isolate CiLV-C_PY_Asu02 was obtained when most of the samples had been already evaluated. Therefore, information on the current distribution of ASU clade viruses, if they are still circulating, is not available. An *in silico* analysis indicated that in case viruses of the clade ASU might be present in the evaluated samples, primers used in the detection of members of the clades CRD and SJP would not be able to detect them, at least under the thermal cycling conditions performed in this study.

A broad-based analysis of the data indicated the presence of members of the clade CRD distributed between commercial (141/186; 75.8%) and non-commercial (45/186, 24.2%) orchards all over the period 1932–2020. In contrast, members of the clade SJP were almost exclusively found in commercial orchards in the citrus belt SP-MG (Table 1 and Figure 5). In commercial orchards, from the samples collected during the period 2003–2020, 64% of lesions (240/375) were infected with viruses of the clade SJP, 16.2% with CRD (61/375), and 19.8% (75/375) exhibited mixed infections involving viruses of the two clades (Figure 5). Interestingly, out of commercial orchards, isolates of the clade SJP were detected in only two samples collected in a backyard tree in São José do Rio Preto, SP, in the neighborhood of the citrus belt SP-MG.

### CiLV-C SJP and CRD Subpopulations Are Mainly Under Purifying Selection

Analysis based on the nucleotide sequences of *p29* and *p32* suggested purifying selection on the CiLV-C subpopulations CRD and SJP (Table 5). The strength of negative selection is weaker on *p32* (ω = 0.137) than on *p29* (ω = 0.278). When subpopulations were compared by each ORF, the highest values corresponded to *p29* of the clade CRD (ω*_*p29*_* = 0.448). Consistent with the low ω values detected, a large number of amino acids under purifying selection were identified across the amino acid sequences of P29 and MP, as supported by the FUBAR, FEL, and MEME programs implemented in Datamonkey 2.0 (Weaver et al., 2018; Supplementary Table 5). Much of the positions detected under negative selection using the FEL method matched with those described in a previous report (Ramos-González et al., 2016).

### CiLV-C SJP and CRD Are Two Genetically Distinct and Expanding Subpopulations

*Snn*, *Fst*, *Kst*, and *Hst* tests were implemented to evaluate the genetic differentiation between the CiLV-C subpopulations. *Snn* and *Fst* values ≥ 1 showed a highly structured population with significant genetic differentiation between the clades CRD and SJP (Table 6). *Kst* and *Hst* values indicated a higher level of genetic differentiation considering the *p29* than the *p32* sequences. Gene flow (*Nm*) values calculated for both ORF sequences were smaller than 0.03 indicating that the gene flow among populations was infrequent or almost non-existent (Table 6).

**TABLE 6:** Population genetic differentiation analysis based on ORFs *p29* (795 nts) and *p32* (288 nts, partial) of CiLV-C.

ORF	Subpopulation	*Snn*	*K*_*st*_^*a*^	*H*_*st*_^*a*^	*F*_*st*_	*Nm*
*p29*	CRD and SJP	1.00000	0.40534	0.00246	0.93675	0.03
*p32*	CRD and SJP	1.00000	0.50932	0.07276	0.94339	0.03

*^a^p-value: 0.01 < p < 0.05.*

*Snn, nearest-neighbor statistic; Hst, Hudson’s test statistics; Kst, nucleotide-based statistics; Fst, Wright’s fixation index, and Nm, gene flow.*

Moreover, the molecular variance (AMOVA) test carried out for the detection of genetic differentiation between the CiLV-C subpopulations revealed the largest variance between the subpopulations CRD and SJP (∼93%), whereas it reached only 7% within each subpopulation (Table 7). With AMOVA statistic values close to 1, the *Fst* results based on *p29* and *p32* allowed us to refute the Null hypothesis of the non-differentiation between CiLV-C subpopulations.

**TABLE 7:** Analysis of molecular variance (AMOVA) for CiLV-C sub-populations of the clades CRD and SJP, based on ORFs *p29* (795 nts) and *p32* (288 nts, partial).

Source of variance		d.f.^*a*^	Sum of squares	Variance components	Percentage of variation	AMOVA statistics	*p*-value
***p29***	Among sub-populations	1	5185.937	55.66483 Va	94.07	0.94067	0.00
	Within sub-populations	187	656.593	3.51119 Vb	5.93		
	Total	188	5842.529	59.17602			
***p32***	Among sub-populations	1	2258.226	20.22056 Va	92.49	0.92491	0.00
	Within sub-populations	267	438.320	1.64165 Vb	7.51		
	Total	268	2696.546	21.86221			

*^*a*^Degrees of freedom.*

Neutrality tests of the CiLV-C subpopulations were estimated using three statistic tests (Fu and Li’s D and F, Fu’s Fs, and Tajima’s D) (Table 8). All values for *p29* and *p32* were negative or non-significant. These results suggested that the CiLV-C subpopulations are not neutral, but possibly expanding. To further address this question, we determined whether the data fit the sudden expansion model using the sum of square deviations (SSD) and Harpending’s Raggedness index (HRI). Both SSD and HRI values were non-significant (Table 8), supporting the hypothesis of population expansion.

**TABLE 8:** Genetic diversity indices, pairwise mismatch distributions, and neutrality tests (Fu and Li’s, Fu’s Fs, and Tajima’s D) based on ORFs *p29* (795 nts) and *p32* (288 nts, partial) of CiLV-C.

ORF	Sub-population	*s*	*k*	*θ*	Mismatch		Neutrality tests			
						
					SSD^*a*^	HRI^*a*^	Fu and Li’s D^*b*^	Fu and Li’s F^*b*^	Fu’s Fs^*c*^	Tajima’s D
*p29*	SJP	138	6.383	144	0.0069	0.0075	−5.59231	−5.12367	−127.897	−2.54236^*d*^
	CRD	154	7.841	158	0.0048	0.0033	−6.13159	−5.57164	−86.537	−2.55671^*d*^
*p32*	SJP	50	2.908	51	0.0235	0.0700	−5.86479	−5.01720	−33.099	−1.99819^*e*^
	CRD	27	1.494	28	0.0718	0.2135	−4.67051	−4.50782	−29.520	−2.28804^*f*^

*^a^Not significant p-values.*

*^b^p < 0.02.*

*^c^None of the statistics gave significant p-values.*

*^d^p < 0.001.*

*^e^p < 0.05.*

*^f^P < 0.01.*

*s, number of segregating sites; k, mean number of nucleotide differences; θ, mean mutation rate per site; SSD, sum of squared deviation; HRI, Harpending’s Raggedness index.*

### The Most Recent Common Ancestor of CiLV-C Lineages Dates Back, at Least, Approximately 1,500 Years Ago

**FIGURE 6:**
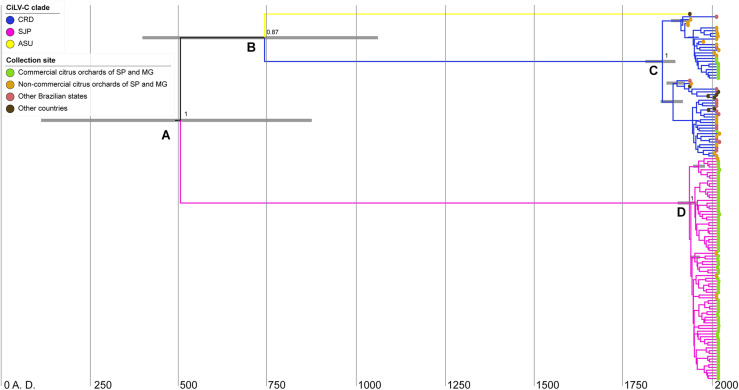
Bayesian maximum-clade-credibility time-scaled phylogenetic tree using the concatenated sequences of the *p29* (795 nts) and *p32* (288 nts, partial) ORFs from 132 CiLV-C isolates collected in the period 1932–2020 in South America. Horizontal gray bars on nodes **(A–D)** indicate the uncertainty for the date of each node (95% highest posterior density—HPD—intervals). Figures near the main nodes represent the posterior probability values. The phylogenetic tree was edited using IcyTree.

To investigate the evolutionary dynamics and the time to the most recent common ancestor (tMRCA) of CiLV-C lineages, two MCC trees were constructed using two datasets. The topology of both trees was similar (Figure 6 and Supplementary Figure 2). However, the tree using the concatenated *RdRd-p29-p61-p32-p24* reached only moderated values of ESS (100 ≤ ESS ≤ 200) for some statistic parameters, likely as a consequence of the lower number of sequences (*n* = 23). The MCC tree generated with the concatenated *p29-p32*, with a larger dataset (*n* = 132), showed a moderate temporal signal (correlation coefficient = 0.3 and *R*^2^ = 0.26, Supplementary Figure 3), and ESS values always > 300. This MCC tree included sequences of isolates collected in Argentina (*n* = 4), Panama (*n* = 1), Paraguay (*n* = 1), and Brazil (*n* = 126) during the period 1932–2020. Therefore, we selected the tree generated with the concatenated *p29*–*p32* as the best representative of the evolutionary history of CiLV-C in the Americas.

Based on the concatenated *p29*–*p32*, the most recent common ancestor (MRCA) of the three CiLV-C lineages dates back to 500 A.D. [supported by a posterior probability (PP) of 1 and 95% highest probability density (HPD) of 115–875 years A.D.] (node A in Figure 6). This ancestral virus diverged into viruses that gave rise to two lineages: SJP and another one, intermediary, that subsequently diverged into the lineages ASU and CRD (node B). The MRCA of the lineages ASU and CRD dates back to 740 A.D. (PP = 0.87 and HPD 95% = 400–1061 years A.D.). Hence, the diversification events represented in nodes A and B overlap in the posterior probability distributions (Figure 6). Diversification of clade CRD happened around 160 years ago, ∼1860 A.D. (node C, PP = 1 and HPD 95% = 1812–1897 years A.D.), whereas the diversification of clade SJP occurred ∼1940 A.D., less than 100 years ago (node D, PP = 1 and HPD 95% = 1856–1918 years A.D.) (Figure 6). Despite the moderate ESS of the MCC based on the concatenated *RdRd-p29-p61-p32-p24*, for the sake of a better understanding of the evolutionary viral process, the tMRCA of the three CiLV-C lineages was also assessed considering that dataset. The virus representing the MRCA of the main lineages dates back to ∼4000 B.C. (Supplementary Figure 2).

## Discussion

Citrus leprosis (CL) disease is a serious multi-etiological viral pathology affecting citrus in Latin America. In Brazil, the form of CL caused by the cilevirus CiLV-C is prevalent and, economically, the most detrimental virus-induced disease affecting the citrus orchards (Ramos-González et al., 2018). A former study reported the existence of two viral clades inside the population of CiLV-C (Ramos-González et al., 2016), but the ecology of these subpopulations could not be deeply assessed due to the limited number of viral isolate sequences available. In the present approach, which includes both fresh and herbarium citrus samples infected by CiLV-C, we obtained the complete and partial nucleotide sequences of the viral ORFs *p29* and *p32*, respectively, from 268 viral isolates. Among them, we revealed the near-complete genomes of 18 isolates, which were mostly collected during the first half of the last century.

Global analyses of the generated dataset by phylogenetic and populational statistical tools confirmed the existence of the two previously identified clades CRD and SJP (Ramos-González et al., 2016), whereas negative results of the Fu and Li’s D and Tajima’s D neutrality tests and the non-significative pairwise mismatch distribution indicated the expansion of the viral population or more likely a population that frequently undergoes bottlenecks (Table 8). Analyses also showed that CRD and SJP subpopulations are genetically well-differentiated (F*st* ≥ 0.92), have very low genetic diversity (π∼0.01) where almost each haplotype is unique (0.8 < Hd < 0.99), and, as a whole, are under purifying selection (ω < 0.5). Such values are the quantitative expression ensuing from the sum of biological factors underlying the CL pathosystem, which, in general, may lead the CiLV-C subpopulations to continuous bottlenecks (Ramos-González et al., 2016). CiLV-C has a very limited natural known host range, infects a reduced number of cells around the inoculation sites, and, in nature, is exclusively transmitted by *Brevipalpus* mites, the only mean by which the virus can reach new infection foci even within a single leaf due to the absence of systemic movement capacity (Freitas-Astúa et al., 2018). Interestingly, the population of coffee ringspot virus, a dichorhavirus with some biological features comparable to those of CiLV-C, e.g., absence of systemic infection, few natural hosts, and *Brevipalpus* mite transmission, also displays low variability and purifying selection (Ramalho et al., 2016).

While the vast majority of the samples evaluated in this study were infected by CiLV-C isolates of the clades CRD and SJP, the herbarium sample collected in Paraguay, in 1937, revealed to be unique (Figures 4, 6). In the phylogenetic analyses using either individual ORFs or complete genomic sequences, the isolate PY_Asu02 was separated into a third branch, called clade ASU (Figure 4). The genome of CiLV-C_PY_Asu02 has the typical organization of cileviruses, with roughly 86% nucleotide sequence identity with the reference genomes of the clades CRD and SJP. Notably, the stretch encompassing the 5′-end of its RNA2 shows high conservation with members of the other two clades and likely originated by recombination (Figure 3), as also observed in other CiLV-C strains (Ramos-González et al., 2016). Recombinant strains can result in viruses with improved virulence, best adaptability to a changing environment, or expansion in the host ranges and vector species (García-Arenal et al., 2001). Even though the role of the 5′-end of the CiLV-C RNA2 is not well understood yet, the highly conserved nucleotide sequence across viruses of the three CiLV-C clades (Figure 2) highlights the participation of this region in viral biology and, particularly, points out the involvement of recombination events shaping the genome of cileviruses.

The set of CiLV-C infected samples gathered in this work is the largest ever undertaken. Its composition is heterogeneous, showing temporal and geographical biases following the trend of the relative importance of citrus crop and the incidence of CL across Latin America. Despite this, the holistic analysis of the dataset discloses ecological relationships between members of the clades CRD and SJP whose extension and authenticity still need to be proven, e.g., (*i*) inside the citrus belt SP-MG, viruses of both lineages are unevenly distributed, (*ii*) members of the clade SJP are more frequently found in single infection (63%) than those of the CRD (16%), (*iii*) mixed infections in the same lesion were detected in 20% of the samples, whereas (*iv*) simultaneous infection of viruses of the clades CRD-SJP in the same orchard or tree accounts for 1% of samples (Figure 5), (*v*) viruses of the clade SJP were detected neither in areas outside the citrus belt SP-MG nor in samples collected before 2015, and (*vi*) except for the isolate PY_Asu02, which belongs to clade ASU, all other samples collected out of the citrus belt SP-MG were infected by viruses of the clade CRD, including a sample from an organic commercial orchard collected in Pará, Brazil. The higher prevalence of viruses of the clade SJP in commercial orchards of the citrus belt SP-MG suggests this subpopulation could have some adaptive advantages over those in the CRD one. Preliminary studies on the diversity of *Brevipalpus* mites in Brazil revealed a large genetic variability of the *B. yothersi* population, however, the association, if any, between a given mite haplotype and any host plant or geographical origin remains to be addressed (Sánchez-Velázquez et al., 2015; Salinas-Vargas et al., 2016). Similarly, the nature of the *Brevipalpus* mite interaction with clade-specific CiLV-C strains and its significance on virus ecology are also lacking. On the other hand, the existence of a large number of CL lesions with mixed infections gives support to the recombination events detected *in silico* and suggests the simultaneous transmission of viruses from different clades by a single mite, and/or the sequential arrival of mites bearing a single viral strain to the same lesion. Conceivably all possibilities may happen in nature, but whatever is the case, the increased attractiveness of *Brevipalpus* mites by CiLV-C-infected leaves (Arena et al., 2016) might be a factor contributing to the occurrence of mixed infections.

Natives of Southwest Asia, plants of the genus *Citrus* have spread worldwide and their introduction into America, in the early sixteenth century, is intrinsically linked to the advent of the European colonization. Commercial exploration of the citrus crop in Brazil began in the seventeenth century (Carvalho et al., 2019; Passos et al., 2019), but expanded and became one of the most important Brazilian commodities in the middle of the twentieth century (Mattos et al., 2005; Neves et al., 2011; Carvalho et al., 2019). According to the phylodynamic analysis based on *p29-p32*, the most recent common ancestor (tMRCA) of CiLV-C lineages was dated in 500 A.D., whereas intensive but less deep diversification processes inside the clades CRD and SJP have been occurring from the nineteenth century. Based on a second dataset that included the sequences *RdRp-p29-p61-p32-p24* of 23 isolates of the three lineages, the MRCA of the main lineages dates back more than 3000 years before. However, since the MCC quality parameters with the second dataset only reached moderate ESS values, the resultant tMRCA must be only considered as a framework for further evaluations. It could be expected that the analysis of new sequences will allow higher accuracy in the determination of tMRCA providing a better description of the evolutionary history of CiLV-C lineages. Regardless of this, bringing together the timelines described by the two datasets we can conclude that (*i*) ancestors of the three viral clades might have been originated in contact with native ecosystems of South America and (*ii*), the expansion of the citriculture in Brazil and other countries in the region, has contributed, although in a low rate, with the intra-clade diversifications of CiLV-C.

CL is considered an emerging disease affecting a crop that, at least in Brazil, encompasses a relatively low genetic diversity and high-density plantings. Increasing pieces of evidence strongly hypothesized that wild ecosystems are a major source of diversity of plant viruses, which have co-evolved with their wild hosts long before they were domesticated (Pagan and Holmes, 2010; Rodríguez-Nevado et al., 2017). In addition to some *Citrus* spp. and their hybrids, only plants of the species *Commelina benghalensis* and *Swinglea glutinosa* are reported as natural hosts of CiLV-C (León et al., 2008; Nunes et al., 2012; Garita et al., 2014; Freitas-Astúa et al., 2018), all of them are exotic to the Americas (Weniger et al., 2001; Webster et al., 2005). Therefore, the natural host range of CiLV-C is likely not yet fully known or the interaction with its wild hosts might no longer exist in nature. The distribution and dispersion of cileviruses are mediated by the polyphagous *Brevipalpus* mites, capable of feeding on more than 150 genera of plants, including several crops, ornamentals, and forest plants (Childers et al., 2003). Consequently, the CiLV-C vector is likely the main path between native and exotic plants in the Americas (Freitas-Astúa et al., 2018). Similar to CiLV-C, the cilevirus CiLV-C2 also infects citrus plants and seems to have a narrow range of known natural hosts, e.g., *Citrus* spp., *S. glutinosa, Hibiscus* sp., and *Dieffenbachia* sp. (Melzer et al., 2013; Roy et al., 2015, 2018). In contrast, PfGSV is the only known cilevirus infecting native plants of the Americas, e.g., *Passiflora* spp. (Kitajima et al., 1997; Ramos-González et al., 2020). This virus can be found naturally infecting more than twenty plant species and, notably, symptoms are not lesions as locally restricted as those observed in citrus plants infected by CiLV-C. Since the interaction of CiLV-C with citrus plants is dominated by a hypersensitive-like response (Arena et al., 2016, 2020), the expansion of the host range to *Citrus* spp. might have resulted in CiLV-C fitness reduction. Alternatively, based on the low variability of viruses inside each CiLV-C clade, a thought-provoking question is whether CiLV-C can be considered a specialist virus, whose interaction with citrus is carefully selected to act as a helper (effector-like) factor of the mite infestation to suppress the plant defenses (Arena et al., 2016, 2018, 2020).

Altogether, this study provides the most complete snapshot of the CiLV-C population to date. Throughout molecular epidemiology analyses, we have revealed the structure, sources of the genetic variability, and forces involved in the recent evolution of this viral population. The evolutionary history of CiLV-C may be strongly influenced by interaction with its main known host, *Citrus* spp. during a relatively short period, which at most includes the last 500 years. Maximum values of variability inside the population are typified by its subdivision into the clades ASU, first identified in this study, CRD, and SJP. These three clades are the outcome of diversification processes that occurred before the viral contact with the citrus host. Moreover, besides the highly frequent bottlenecks as a result of mite transmission, the incompatible host-virus interaction with an intensive crop with a relatively low genetic variability, likely prevents the expansion and diversification of the CiLV-C subpopulations. In practical terms, our results confirm the possibility of recovering viral sequences present in herbarium citrus leaf samples despite the low RIN values of the RNA extracts, as previously successfully described using dried fruit peels (Hartung et al., 2015). Moreover, the current study reinforces the prevalence and wide distribution of CiLV-C in the largest citrus commercial area of Brazil and reveals the urgency for updating detection systems able to identify the presence of CiLV-C variants whose epidemiological profiles are currently unknown.

## Data Availability Statement

The datasets presented in this study can be found in the NCBI Genbank Database. The accession numbers can be found in Supplementary Table 1.

## Author Contributions

CC-J and PR-G conceptualized and wrote the manuscript. CC-J, PR-G, MP-B, and RH worked in laboratory analyses. CC-J, PR-G, and RF worked on the formal analysis. PR-G, AV, and JF-A supervised the study. JF-A and EK were responsible for funding acquisition. AM and RB assisted in the collection of fresh citrus samples. All authors reviewed the final manuscript.

## Conflict of Interest

The authors declare that the research was conducted in the absence of any commercial or financial relationships that could be construed as a potential conflict of interest.

## Footnotes

1 https://www.fundecitrus.com.br/

2 https://www.ncbi.nlm.nih.gov/labs/virus/vssi/#/

3 https://beast2.blogs.auckland.ac.nz/increasing-esss/

## Supplementary Material

Supplementary Figure 1 Bayesian maximum clade credibility tree inferred using a Markov Chain Monte Carlo (MCMC) of the CiLV-C of the lineages CRD (blue branches), SJP (pink branches), and ASU (yellow branches) based on *p29* **(A)** and *p32* **(B)** ORFs. CiLV-C2 sequences (black branches) were used as an outgroup and trees were generated with 6,000,000 generations. The color-coded based collection sites show whether the sample was obtained from commercial orchards in the citrus belt of São Paulo (SP) and Minas Gerais (MG) states (in green), from non-commercial citrus trees of SP and MG (in gold), from other Brazilian states (in mauve), or other countries, i.e., Argentina, Colombia, Mexico, Panama, and Paraguay (in brown).

Supplementary Figure 2 Bayesian maximum-clade-credibility (MCC) time-scaled phylogenetic tree **(A)** and analyses of the temporal signal by linear regression approach **(B)** using the complete sequence of *RdRp*–*p29*–*p61*–*p32*–*p24* ORFs from 23 CiLV-C isolates collected in the period 1932–2020 in South America. **(A)** Horizontal gray bars on nodes A, B, C, and D indicate the uncertainty for the date of each node (95% highest posterior density—HPD—intervals). Figures near the main nodes represent the posterior probability values. The color-coded based collection sites show whether the sample was obtained from commercial orchards in the citrus belt of São Paulo (SP) and Minas Gerais (MG) states (in green), from non-commercial citrus trees of SP and MG (in gold), from other Brazilian states (in mauve), or other countries, i.e., Argentina, Colombia, Mexico, Panama, and Paraguay (in brown). The phylogenetic tree was edited using IcyTree; **(B)** The temporal signal was calculated using TempEst v. 1.5.3 software. Root-to-tip divergence as a function of sampling time for Maximum likelihood non-clock tree generated by IQtree v. 1.5.5 software.

Supplementary Figure 3 Analyses of the temporal signal by linear regression approach from the concatenate of the *p29* (795 nt) and partial *p32* (288 nt) of 132 isolates of CiLV-C using TempEst v. 1.5.3 software. Root-to-tip divergence as a function of sampling time for Maximum likelihood non-clock tree generated by IQtree v. 1.5.5 software.

Supplementary Table 1 Complete list of plant samples and citrus leprosis virus C (CiLV-C) sequences analyzed in this study.

Supplementary Table 2 Nucleotide and haplotypic diversities of ORFs *p29* and *p32* of CiLV-C. Nucleotide sequences were amplified and cloned from samples of infected *Citrus sinensis* trees collected in commercial orchards of the citrus belt São Paulo—Minas Gerais, Brazil, in 2017.

Supplementary Table 3 Nucleotide and deduced amino acid identities (%) among CiLV-C isolates described in this study and the type members of the clades CRD (CiLV-C_BR_SP_Crd01, GenBank accession numbers NC008169 and NC008170) and SJP (CiLV-C_BR_SP_SJP01, GB acc. numbers KP336746 and KP336747).

Supplementary Table 4 Recombination events detected in CiLV-C sequences determined by RDP software version 5.5.

Supplementary Table 5 Summary of selection analysis of the *p29* and *p32* in CiLV-C.
