# Supplementary figures and images for: Molecular Epidemiology of Citrus Leprosis Virus C: A New Viral Lineage and Phylodynamic of the Main Viral Subpopulations in the Americas
Source: Front Microbiol. 2021 Apr 29;12:641252. doi: 10.3389/fmicb.2021.641252 (PMC8116597; doi:10.3389/fmicb.2021.641252)

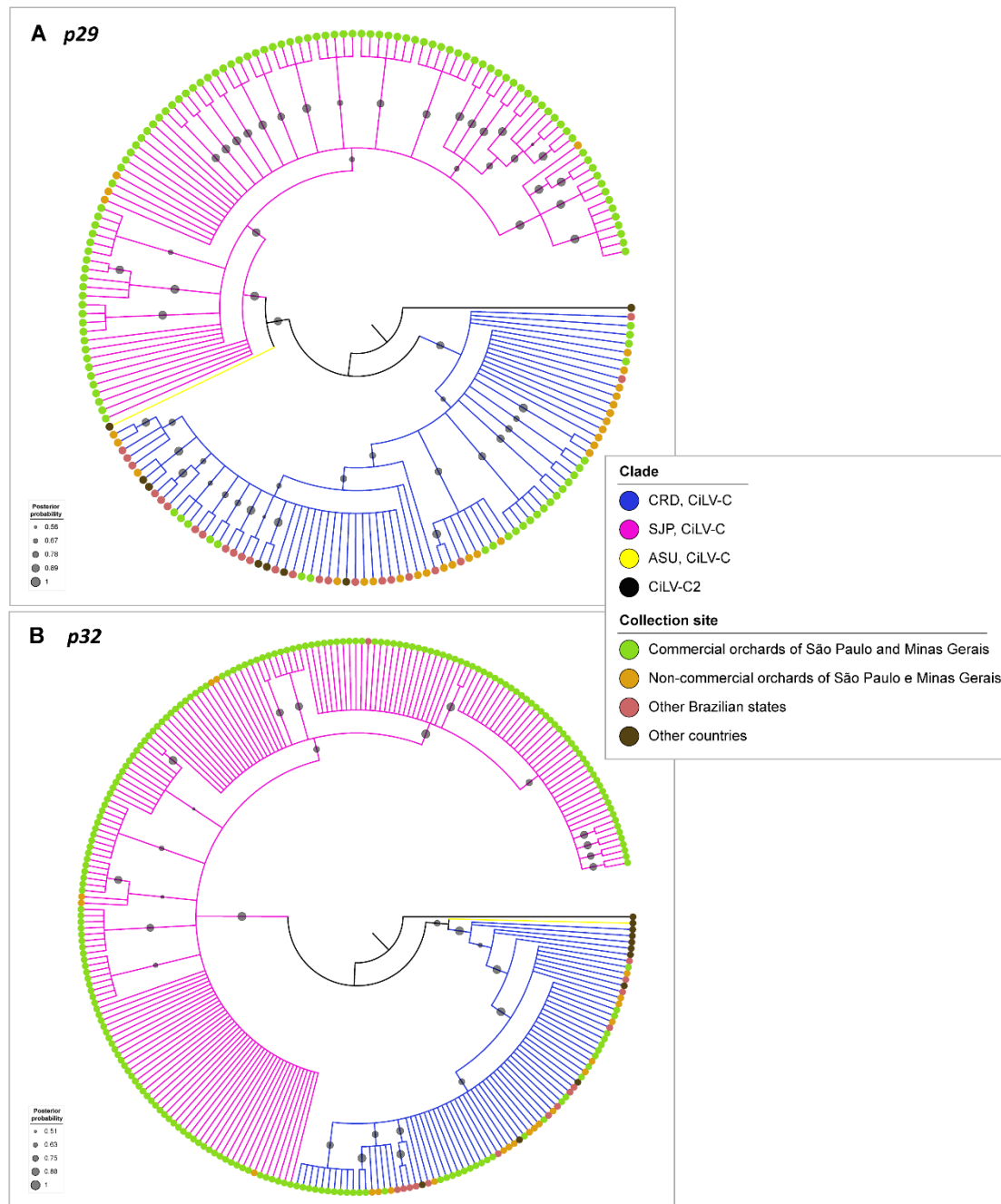

**Supplementary Figure S1.**

Supplement: Supplementary Figure 1 — Bayesian maximum clade credibility tree inferred using a Markov Chain Monte Carlo (MCMC) of the CiLV-C of the lineages CRD (blue branches), SJP (pink branches), and ASU (yellow branches) based on p29 (A) and p32 (B) ORFs. CiLV-C2 sequences (black branches) were used as an outgroup and trees were generated with 6,000,000 generations. The color-coded based collection sites show whether the sample was obtained from commercial orchards in the citrus belt of São Paulo (SP) and Minas Gerais (MG) states (in green), from non-commercial citrus trees of SP and MG (in gold), from other Brazilian states (in mauve), or other countries, i.e., Argentina, Colombia, Mexico, Panama, and Paraguay (in brown). [file Image_1.PDF]

**A**

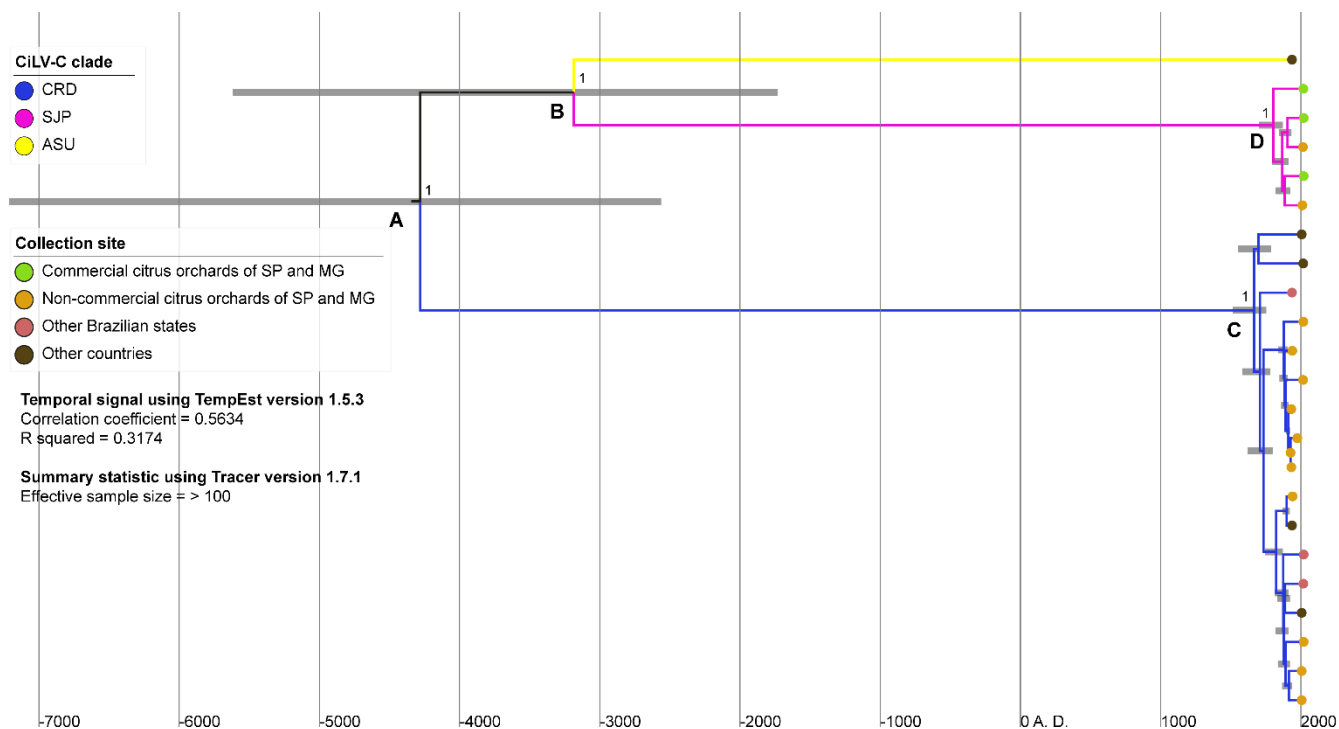

**B**

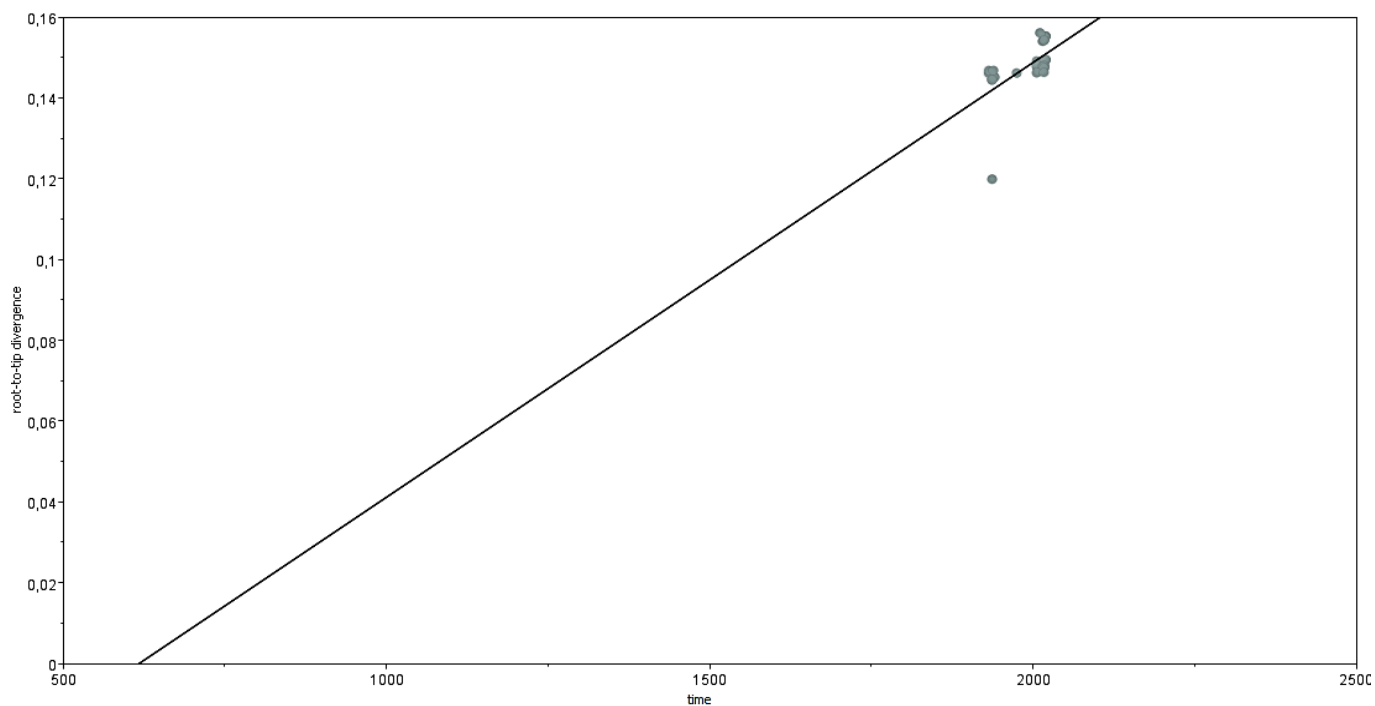

**Supplementary Figure S2.**

Supplement: Supplementary Figure 2 — Bayesian maximum-clade-credibility (MCC) time-scaled phylogenetic tree (A) and analyses of the temporal signal by linear regression approach (B) using the complete sequence of RdRp–p29–p61–p32–p24 ORFs from 23 CiLV-C isolates collected in the period 1932–2020 in South America. (A) Horizontal gray bars on nodes A, B, C, and D indicate the uncertainty for the date of each node (95% highest posterior density—HPD—intervals). Figures near the main nodes represent the posterior probability values. The color-coded based collection sites show whether the sample was obtained from commercial orchards in the citrus belt of São Paulo (SP) and Minas Gerais (MG) states (in green), from non-commercial citrus trees of SP and MG (in gold), from other Brazilian states (in mauve), or other countries, i.e., Argentina, Colombia, Mexico, Panama, and Paraguay (in brown). The phylogenetic tree was edited using IcyTree; (B) The temporal signal was calculated using TempEst v. 1.5.3 software. Root-to-tip divergence as a function of sampling time for Maximum likelihood non-clock tree generated by IQtree v. 1.5.5 software. [file Image_2.PDF]

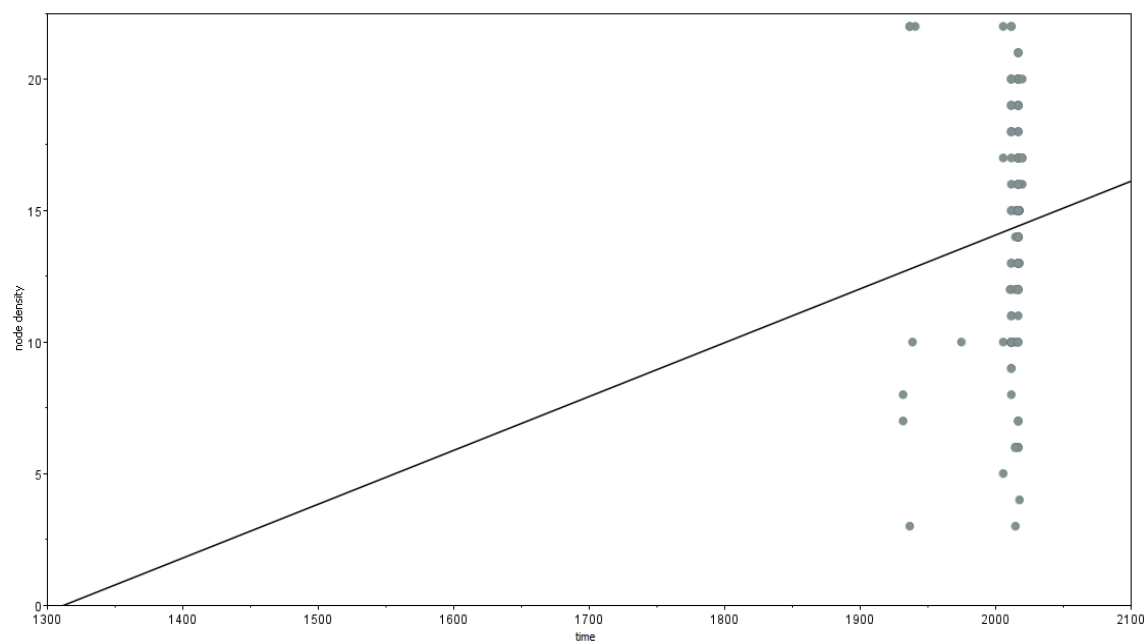

**Supplementary Figure S3.**

Supplement: Supplementary Figure 3 — Analyses of the temporal signal by linear regression approach from the concatenate of the p29 (795 nt) and partial p32 (288 nt) of 132 isolates of CiLV-C using TempEst v. 1.5.3 software. Root-to-tip divergence as a function of sampling time for Maximum likelihood non-clock tree generated by IQtree v. 1.5.5 software. [file Image_3.PDF]
