# Supplementary material for: Molecular Epidemiology of Citrus Leprosis Virus C: A New Viral Lineage and Phylodynamic of the Main Viral Subpopulations in the Americas
Source: Front Microbiol. 2021 Apr 29;12:641252. doi: 10.3389/fmicb.2021.641252 (PMC8116597; doi:10.3389/fmicb.2021.641252)
Supplement: Supplementary Table 1 — Complete list of plant samples and citrus leprosis virus C (CiLV-C) sequences analyzed in this study. [file Table_1.docx]

**Supplementary Table S1**. Complete list of plant samples and citrus leprosis virus C (CiLV-C) sequences analyzed in this study.

| **Sample no.** | **Host no.** | **Host (species/variety)** | **Sample collection^a^** | **Tissue type** | **Collection site** | **Orchard type** | **Collection year** | **CiLV-C phylogenetic clade^c^** | **Isolate identification^d^** | **Genomic region analyzed^e^** | **GenBank accession number** |
| --- | --- | --- | --- | --- | --- | --- | --- | --- | --- | --- | --- |
|  |  |  |  |  | **City, state, country^b^** |  |  |  |  |  |  |
| **Stored in the Herbarium of Instituto Biológico** | | | | | | | | | | | |
| 1 | 1 | *Citrus sinensis* | pool | Leaf | Jacareí, SP, BR | non-commercial | 1932 | CRD | BR_SP_Jac01 | RNA1 | MT554530 |
|  |  |  |  |  |  |  |  |  |  | RNA2 | MT554544 |
| 2 | 2 | *C. sinensis* | pool | Leaf | Piracicaba, SP, BR | non-commercial | 1932 | CRD | BR_SP_Prb04 | RNA1 | MT554535 |
|  |  |  |  |  |  |  |  |  |  | RNA2 | MT554549 |
| 3 | 3 | *C. sinensis* | pool | Leaf | Uruguaiana, RS, BR | non-commercial | 1937 | CRD | BR_RS_Urg01 | RNA1 | MT554538 |
|  |  |  |  |  |  |  |  |  |  | RNA2 | MT554552 |
| 4 | 4 | *C. sinensis* | pool | Leaf | Santa’Ana, Misiones, AR | non-commercial | 1937 | CRD | AR06 | RNA1 | MT554528 |
|  |  |  |  |  |  |  |  |  |  | RNA2 | MT554542 |
| 5 | 5 | *C. sinensis* | pool | Leaf | Assuncion, PY | not available | 1937 | ASU | PY_Asu02 | RNA1 | MT554532 |
|  |  |  |  |  |  |  |  |  |  | RNA2 | MT554546 |
| 6 | 6 | *C. sinensis* | pool | Leaf | Limeira, SP, BR | non-commercial | 1939 | CRD | BR_SP_Lim01 | RNA1 | MT554531 |
|  |  |  |  |  |  |  |  |  |  | RNA2 | MT554545 |
| 7 | 7 | *C. sinensis* | pool | Leaf | São Paulo, SP, BR | non-commercial | 1941 | CRD | BR_SP_SPa11 | RNA1 | MT554551 |
|  |  |  |  |  |  |  |  |  |  | RNA2 | MT554537 |
| 8 | 8 | *C. sinensis* | pool | Leaf | Jaboticabal, SP, BR | non-commercial | 1975 | CRD | BR_SP_Jbt02 | RNA1 | MT554529 |
|  |  |  |  |  |  |  |  |  |  | RNA2 | MT554543 |
| **Stored at - 80ºC** | | | | | | | | | | | |
| 9 | 9 | *Citrus reticulata* | pool | Leaf | Amparo, SP, BR | commercial | 2003 | CRD | BR_SP_Amp03 | *p29* | NS^f^ |
|  |  |  |  |  |  |  |  |  |  | *p32* | NS |
| 10 | 10 | *Citrus* sp. | pool | Leaf | Yapacaní, BO | non-commercial | 2003 | CRD | BO_ Ypc01 | *p29* | NS |
|  |  |  |  |  |  |  |  |  |  | *p32* | NS |
| 11 | 11 | *Citrus* sp. | pool | Leaf | Maringá, PR | non-commercial | 2004 | CRD | BR_PR_Mgf02 | *p29* | NS |
|  |  |  |  |  |  |  |  |  |  | *p32* | NS |
| 12 | 12 | *C. sinensis* | pool | Leaf | Argentina | non-commercial | 2006 | CRD | AR04 | RNA1 | MT554526 |
|  |  |  |  |  |  |  |  |  |  | RNA2 | MT554540 |
| 13 | 13 | *C. sinensis* | pool | Leaf | Colombia | non-commercial | 2008 | CRD | CO02 | *p29* | NS |
|  |  |  |  |  |  |  |  |  |  | *p32* | NS |
| 14 | 14 | *Citrus* sp. | pool | Leaf | Paraguay | non-commercial | 2010 | CRD | PY03 | *p29* | NS |
|  |  |  |  |  |  |  |  |  |  | *p32* | NS |
| 15 | 15 | *C. sinensis* | pool | Leaf | Amparo, SP, BR | commercial | 2012 | CRD | BR_SP_Amp02 | *p29* | MN954965 |
|  |  |  |  |  |  |  |  |  |  | *p32* | NS |
| 16 | 16 | *C. sinensis* | pool | Leaf | Araras, SP, BR | non-commercial | 2012 | CRD | BR_SP_Ara02 | *p29* | MN954966 |
|  |  |  |  |  |  |  |  |  |  | *p32* | NS |
| 17 | 17 | *C. sinensis* | pool | Leaf | Borborema, SP, BR | commercial | 2012 | CRD | BR_SP_Brm03 | *p29* | MN954987 |
|  |  |  |  |  |  |  |  |  |  | *p32* | NS |
| 18 | 18 | *C. sinensis* | pool | Leaf | Brasília, DF, BR | non-commercial | 2012 | CRD | BR_DF_Bsb02 | *p29* | MN954988 |
|  |  |  |  |  |  |  |  |  |  | *p32* | NS |
| 19 | 19 | *C. sinensis* | pool | Leaf | Colina, SP, BR | commercial | 2012 | CRD | BR_SP_Cln02 | *p29* | MN955004 |
|  |  |  |  |  |  |  |  |  |  | *p32* | NS |
| 20 | 20 | *C. sinensis* | pool | Leaf | Comendador Gomes, MG, BR | commercial | 2012 | CRD | BR_MG_Cgz02 | *p29* | MN955010 |
|  |  |  |  |  |  |  |  |  |  | *p32* | NS |
| 21 | 21 | *C. sinensis* | pool | Leaf | Conchal, SP, BR | commercial | 2012 | CRD | BR_SP_Cch02 | *p29* | MN955011 |
|  |  |  |  |  |  |  |  |  |  | *p32* | NS |
| 22 | 22 | *C. sinensis* | pool | Leaf | Cordeirópolis, SP, BR | non-commercial | 2012 | CRD | BR_SP_Crd04 | *p29* | MN955012 |
|  |  |  |  |  |  |  |  |  |  | *p32* | NS |
| 23 | 23 | *C. sinensis* | pool | Leaf | Cordeirópolis, SP, BR | non-commercial | 2012 | CRD | BR_SP_Crd05 | *p29* | NS |
|  |  |  |  |  |  |  |  |  |  | *p32* | NS |
| 24 | 24 | *C. sinensis* | pool | Leaf | Cordeirópolis, SP, BR | non-commercial | 2012 | CRD | BR_SP_Crd06 | *p29* | NS |
|  |  |  |  |  |  |  |  |  |  | *p32* | NS |
| 25 | 25 | *C. sinensis* | pool | Leaf | Cordeirópolis, SP, BR | commercial | 2012 | CRD | BR_SP_Crd07 | *p29* | NS |
|  |  |  |  |  |  |  |  |  |  | *p32* | NS |
| 26 | 26 | *C. sinensis* | pool | Leaf | Itu, SP, BR | commercial | 2012 | CRD | BR_SP_Itu01 | *p29* | MN955038 |
|  |  |  |  |  |  |  |  |  |  | *p32* | NS |
| 27 | 27 | *C. sinensis* | pool | Leaf | Londrina, PR, BR | non-commercial | 2012 | CRD | BR_PR_Ldb02 | *p29* | MN955018 |
|  |  |  |  |  |  |  |  |  |  | *p32* | NS |
| 28 | 28 | *C. sinensis* | pool | Leaf | Manaus, AM, BR | non-commercial | 2012 | CRD | BR_AM_Mao02 | *p29* | MN955019 |
|  |  |  |  |  |  |  |  |  |  | *p32* | NS |
| 29 | 29 | *C. sinensis* | pool | Leaf | Mirandópolis, SP, BR | commercial | 2012 | CRD | BR_SP_Mrn02 | *p29* | MN955020 |
|  |  |  |  |  |  |  |  |  |  | *p32* | NS |
| 30 | 30 | *C. sinensis* | pool | Leaf | Palmas, TO, BR | non-commercial | 2012 | CRD | BR_TO_Pnw02 | *p29* | MN955066 |
|  |  |  |  |  |  |  |  |  |  | *p32* | NS |
| 31 | 31 | *C. sinensis* | pool | Leaf | Santo Antônio da Posse, SP, BR | commercial | 2012 | CRD | BR_SP_SAP02 | *p29* | MN955046 |
|  |  |  |  |  |  |  |  |  |  | *p32* | NS |
| 32 | 32 | *C. sinensis* | pool | Leaf | Serra Negra, SP, BR | non-commercial | 2012 | CRD | BR_SP_Sng02 | *p29* | MN955047 |
|  |  |  |  |  |  |  |  |  |  | *p32* | NS |
| 33 | 33 | *C. sinensis* | pool | Leaf | Tanguá, RJ, BR | non-commercial | 2012 | CRD | BR_RJ_Tng02 | *p29* | MN955057 |
|  |  |  |  |  |  |  |  |  |  | *p32* | NS |
| 34 | 34 | *C. sinensis* | pool | Leaf | Tatuí, SP, BR | commercial | 2012 | CRD | BR_SP_Tti02 | *p29* | MN955063 |
|  |  |  |  |  |  |  |  |  |  | *p32* | NS |
| 35 | 35 | *C. sinensis* | pool | Leaf | Terenos, MS, BR | non-commercial | 2012 | CRD | BR_MS_Trn02 | *p29* | MN955064 |
|  |  |  |  |  |  |  |  |  | BR_MS_Trn03 | *p29* | MN955065 |
| 36 | 36 | *C. sinensis* | pool | Leaf | Argentina | non-commercial | 2012 | CRD | AR03 | *p29* | MN954967 |
|  |  |  |  |  |  |  |  |  |  | *p32* | NS |
| 37 | 37 | *Citrus sp.* | pool | Leaf | Argentina | non-commercial | 2012 | CRD | AR07 | *p29* | NS |
|  |  |  |  |  |  |  |  |  |  | *p32* | NS |
| 38 | 38 | *C. sinensis* (Hamlin) | pool | Leaf | Argentina | non-commercial | 2013 | CRD | AR08 | *p29* | NS |
|  |  |  |  |  |  |  |  |  |  | *p32* | NS |
| 39 | 39 | *C. sinensis* (Hamlin) | pool | Leaf | Argentina | non-commercial | 2013 | CRD | AR09 | *p29* | NS |
|  |  |  |  |  |  |  |  |  |  | *p32* | NS |
| 40 | 40 | *Citrus clementina* | pool | Leaf | Argentina | non-commercial | 2013 | CRD | AR10 | *p29* | NS |
|  |  |  |  |  |  |  |  |  |  | *p32* | NS |
| 41 | 41 | *C. sinensis* | pool | Leaf | Piracicaba, SP, BR | non-commercial | 2014 | CRD | BR_SP_Prb05 | *p29* | NS |
|  |  |  |  |  |  |  |  |  |  | *p32* | NS |
| 42 | 42 | *C. sinensis* | pool | Leaf | Araras, SP, BR | commercial | 2014 | CRD | BR_SP_Ara03 | *p29* | NS |
|  |  |  |  |  |  |  |  |  |  | *p32* | NS |
| 43 | 43 | *C. sinensis* | pool | Leaf | Cosmorama, MG, SP | commercial | 2015 | SJP | BR_SP_Csm02 | *p29* | MN955013 |
|  |  |  |  |  |  |  |  |  | BR_SP_Csm03 | *p29* | MN955014 |
| 44 | 44 | *C. sinensis* | pool | Leaf | Sud Mennucci, SP, BR | commercial | 2015 | SJP | BR-SP_SdM02 | *p29* | MN955077 |
|  |  |  |  |  |  |  |  |  |  | *p32* | NS |
| 45 | 45 | *C. sinensis* | pool | Leaf | Sud Mennucci, SP, BR | commercial | 2015 | SJP | BR-SP_SdM04 | *p29* | MN955078 |
|  |  |  |  |  |  |  |  |  |  | *p32* | NS |
| 46 | 46 | *C. sinensis* | pool | Leaf | Sud Mennucci, SP, BR | commercial | 2015 | SJP | BR-SP_SdM06 | *p29* | MN955079 |
|  |  |  |  |  |  |  |  |  |  | *p32* | NS |
| 47 | 47 | *C. sinensis* | pool | Leaf | Sud Mennucci, SP, BR | commercial | 2015 | SJP | BR-SP_SdM08 | *p29* | MN955080 |
|  |  |  |  |  |  |  |  |  |  | *p32* | NS |
| 48 | 48 | *C. sinensis* | pool | Leaf | Sud Mennucci, SP, BR | commercial | 2015 | SJP | BR-SP_SdM10 | *p29* | MN955081 |
|  |  |  |  |  |  |  |  |  |  | *p32* | NS |
| 49 | 49 | *C. sinensis* | pool | Leaf | Sud Mennucci, SP, BR | commercial | 2015 | SJP | BR-SP_SdM12 | *p29* | MN955082 |
|  |  |  |  |  |  |  |  |  |  | *p32* | NS |
| **Fresh tissue** | | | | | | | | | | | |
| 50 | 50 | *Citrus* sp. | pool | Leaf | Argentina | non-commercial | 2015 | CRD | AR11 | *p29* | NS |
|  |  |  |  |  |  |  |  |  |  | *p32* | NS |
| 51 | 51 | *C. sinensis* | pool | Fruit | Barretos, SP, BR | commercial | 2015 | SJP | BR_SP_Bar19 | *p29* | NS |
|  |  |  |  |  |  |  |  |  |  | *p32* | NS |
| 52 | 52 | *C. sinensis* | pool | Fruit | Barretos, SP, BR | commercial | 2015 | SJP | BR_SP_Bar20 | *p29* | NS |
|  |  |  |  |  |  |  |  |  |  | *p32* | NS |
| 53 | 53 | *C. sinensis* | pool | Fruit | Barretos, SP, BR | commercial | 2015 | SJP | BR_SP_Bar21 | *p29* | NS |
|  |  |  |  |  |  |  |  |  |  | *p32* | NS |
| 54 | 54 | *C. sinensis* | pool | Fruit | Barretos, SP, BR | commercial | 2015 | SJP | BR_SP_Bar22 | *p29* | NS |
|  |  |  |  |  |  |  |  |  |  | *p32* | NS |
| 55 | 55 | *C. sinensis* | pool | Fruit | Barretos, SP, BR | commercial | 2015 | SJP | BR_SP_Bar23 | *p29* | NS |
|  |  |  |  |  |  |  |  |  |  | *p32* | NS |
| 56 | 56 | *C. sinensis* | pool | Fruit | Barretos, SP, BR | commercial | 2015 | SJP | BR_SP_Bar24 | *p29* | NS |
|  |  |  |  |  |  |  |  |  |  | *p32* | NS |
| 57 | 57 | *C. sinensis* | pool | Fruit | Itirapina, SP, BR | commercial | 2015 | CRD | BR_SP_Irp01 | *p29* | NS |
|  |  |  |  |  |  |  |  |  |  | *p32* | NS |
| 58 | 58 | *C. reticulata* | pool | Fruit | São Jose do Rio Preto, SP, BR | commercial | 2015 | SJP | BR_SP_SJP06 | *p29* | NS |
|  |  |  |  |  |  |  |  |  |  | *p32* | NS |
| 59 | 59 | *C. reticulata* | pool | Fruit | São Jose do Rio Preto, SP, BR | commercial | 2015 | SJP | BR_SP_SJP07 | *p29* | NS |
|  |  |  |  |  |  |  |  |  |  | *p32* | NS |
| 60 | 60 | *C. sinensis* | pool | Fruit | Adolfo, SP, BR | commercial | 2016 | CRD+SJP | BR_SP_Adf01 | *p29* | NS |
|  |  |  |  |  |  |  |  |  | BR_SP_Adf02 | *p32* | NS |
| 61 | 61 | *C. sinensis* | pool | Fruit | Araraquara, SP, BR | commercial | 2016 | SJP | BR_SP_Arq01 | *p29* | NS |
|  |  |  |  |  |  |  |  |  |  | *p32* | NS |
| 62 | 62 | *C. sinensis* (Valencia) | pool | Fruit | Avaré, SP, BR | commercial | 2016 | CRD | BR_SP_Avr01 | *p29* | NS |
|  |  |  |  |  |  |  |  |  |  | *p32* | NS |
| 63 | 63 | *C. sinensis* | pool | Fruit | Bebedouro, SP, BR | commercial | 2016 | SJP | BR_SP_Beb32 | *p29* | NS |
|  |  |  |  |  |  |  |  |  |  | *p32* | NS |
| 64 | 64 | *C. sinensis* | pool | Leaf | Brasília, DF, BR | non-commercial | 2016 | CRD | BR_DF_Bsb03 | *p29* | NS |
|  |  |  |  |  |  |  |  |  |  | *p32* | NS |
| 65 | 65 | *C. sinensis* | pool | Fruit | Caiabu, SP, BR | commercial | 2016 | CRD+SJP | BR_SP_Cab01 | *p29* | NS |
|  |  |  |  |  |  |  |  |  | BR_SP_Cab02 | *p32* | NS |
| 66 | 66 | *C. sinensis* | pool | Fruit | Colômbia, SP, BR | commercial | 2016 | SJP | BR_SP_Clb10 | *p29* | NS |
|  |  |  |  |  |  |  |  |  |  | *p32* | NS |
| 67 | 67 | *C. sinensis* | pool | Fruit | Colômbia, SP, BR | commercial | 2016 | SJP | BR_SP_Clb11 | *p29* | NS |
|  |  |  |  |  |  |  |  |  |  | *p32* | NS |
| 68 | 68 | *C. sinensis* | pool | Fruit | Colômbia, SP, BR | commercial | 2016 | SJP | BR_SP_Clb12 | *p29* | NS |
|  |  |  |  |  |  |  |  |  |  | *p32* | NS |
| 69 | 69 | *C. sinensis* | pool | Fruit | Colômbia, SP, BR | commercial | 2016 | SJP | BR_SP_Clb13 | *p29* | NS |
|  |  |  |  |  |  |  |  |  |  | *p32* | NS |
| 70 | 70 | *C. sinensis* | pool | Fruit | Colômbia, SP, BR | commercial | 2016 | SJP | BR_SP_Clb14 | *p29* | NS |
|  |  |  |  |  |  |  |  |  |  | *p32* | NS |
| 71 | 71 | *C. sinensis* | pool | Fruit | Colômbia, SP, BR | commercial | 2016 | SJP | BR_SP_Clb15 | *p29* | NS |
|  |  |  |  |  |  |  |  |  |  | *p32* | NS |
| 72 | 72 | *C. sinensis* | pool | Fruit | Colômbia, SP, BR | commercial | 2016 | SJP | BR_SP_Clb16 | *p29* | NS |
|  |  |  |  |  |  |  |  |  |  | *p32* | NS |
| 73 | 73 | *C. sinensis* | pool | Fruit | Comendador Gomes, MG, BR | commercial | 2016 | CRD | BR_MG_Cgz03 | *p29* | NS |
|  |  |  |  |  |  |  |  |  |  | *p32* | NS |
| 74 | 74 | *C. sinensis* | pool | Fruit | Comendador Gomes, MG, BR | commercial | 2016 | CRD | BR_MG_Cgz04 | *p29* | NS |
|  |  |  |  |  |  |  |  |  |  | *p32* | NS |
| 75 | 75 | *C. sinensis* | pool | Fruit | Frutal, MG, BR | commercial | 2016 | SJP | BR_MG_Frt01 | *p29* | NS |
|  |  |  |  |  |  |  |  |  |  | *p32* | NS |
| 76 | 76 | *C. sinensis* | pool | Fruit | Guarantã, SP, BR | commercial | 2016 | SJP | BR_SP_Grt01 | *p29* | NS |
|  |  |  |  |  |  |  |  |  |  | *p32* | NS |
| 77 | 77 | *C. sinensis* | pool | Fruit | Guimbê, SP, BR | commercial | 2016 | SJP | BR_SP_Gua09 | *p29* | NS |
|  |  |  |  |  |  |  |  |  |  | *p32* | NS |
| 78 | 78 | *C. sinensis* | pool | Fruit | Jaboticabal, SP, BR | commercial | 2016 | SJP | BR_SP_Jbt03 | *p29* | NS |
|  |  |  |  |  |  |  |  |  |  | *p32* | NS |
| 79 | 79 | *C. sinensis* | pool | Fruit | Limeira, SP, BR | commercial | 2016 | SJP | BR_SP_Lim02 | *p29* | NS |
|  |  |  |  |  |  |  |  |  |  | *p32* | NS |
| 80 | 80 | *C. sinensis* | pool | Fruit | Olímpia, SP, BR | commercial | 2016 | SJP | BR_SP_Olm01 | *p29* | NS |
|  |  |  |  |  |  |  |  |  |  | *p32* | NS |
| 81 | 81 | *C. sinensis* | pool | Leaf | Piracicaba, SP, BR | non-commercial | 2016 | CRD | BR_SP_Prb02 | RNA1 | MT554533 |
|  |  |  |  |  |  |  |  |  |  | RNA2 | MT554547 |
| 82 | 82 | *C. sinensis* | pool | Leaf | Piracicaba, SP, BR | non-commercial | 2016 | CRD | BR_SP_Prb06 | *p29* | NS |
|  |  |  |  |  |  |  |  |  |  | *p32* | NS |
| 83 | 83 | *C. sinensis* | pool | Fruit | Pirajuí, SP, BR | commercial | 2016 | CRD+SJP | BR_SP_Prj01 | *p29* | NS |
|  |  |  |  |  |  |  |  |  | BR_SP_Prj02 | *p32* | NS |
| 84 | 84 | *C. sinensis* | pool | Fruit | Pirapora, MG, BR | commercial | 2016 | CRD | BR_MG_Prp01 | *p29* | NS |
|  |  |  |  |  |  |  |  |  |  | *p32* | NS |
| 85 | 85 | *C. sinensis* | pool | Fruit | Pirassununga, SP, BR | commercial | 2016 | SJP | BR_SP_Prg06 | *p29* | NS |
|  |  |  |  |  |  |  |  |  |  | *p32* | NS |
| 86 | 86 | *C. sinensis* (Pera) | pool | Fruit | Santa Cruz do Rio Pardo, SP, BR | commercial | 2016 | SJP | BR_SP_SCP01 | *p29* | NS |
|  |  |  |  |  |  |  |  |  |  | *p32* | NS |
| 87 | 87 | *C. sinensis* | pool | Fruit | Tabatinga, SP, BR | commercial | 2016 | SJP | BR_SP_Tbt01 | *p29* | NS |
|  |  |  |  |  |  |  |  |  |  | *p32* | NS |
| 88 | 88 | *C. sinensis* | pool | Fruit | Tremenbé, SP, BR | commercial | 2016 | SJP | BR_SP_Tmb01 | *p29* | NS |
|  |  |  |  |  |  |  |  |  |  | *p32* | NS |
| 89 | 89 | *C. sinensis* | pool | Fruit | Uru, SP, BR | commercial | 2016 | CRD | BR_SP_Uru01 | *p29* | NS |
|  |  |  |  |  |  |  |  |  |  | *p32* | NS |
| 90 | 90 | *C. sinensis* | pool | Leaf | Corrientes, Argentina | non-commercial | 2017 | CRD | AR04 | *p29* | NS |
|  |  |  |  |  |  |  |  |  |  | *p32* | NS |
| 91 | 91 | *C. sinensis* (Folha murcha) | single | Fruit | Aguaí, SP, BR | commercial | 2017 | CRD+SJP | BR_SP_Agi01 | *p29* | MN954955 |
|  |  |  |  |  |  |  |  |  |  | *p32* | MN955083 |
|  |  |  |  |  |  |  |  |  |  | *p24* | NS |
|  |  |  |  |  |  |  |  |  | BR_SP_Agi02 | *p29* | MN954956 |
|  |  |  |  |  |  |  |  |  |  | *p32* | MN955084 |
|  |  |  |  |  |  |  |  |  |  | *p24* | NS |
|  |  |  |  |  |  |  |  |  | BR_SP_Agi03 | *p29* | MN954957 |
|  |  |  |  |  |  |  |  |  |  | *p32* | MN955085 |
|  |  |  |  |  |  |  |  |  |  | *p24* | NS |
|  |  |  |  |  |  |  |  |  | BR_SP_Agi04 | *p29* | NS |
|  |  |  |  |  |  |  |  |  |  | *p32* | MN955086 |
|  |  |  |  |  |  |  |  |  |  | *p24* | NS |
|  |  |  |  |  |  |  |  |  | BR_SP_Agi05 | *p29* | NS |
|  |  |  |  |  |  |  |  |  |  | *p32* | MN955087 |
|  |  |  |  |  |  |  |  |  |  | *p24* | NS |
|  |  |  |  |  |  |  |  |  | BR_SP_Agi06 | *p29* | NS |
|  |  |  |  |  |  |  |  |  |  | *p32* | MN955088 |
|  |  |  |  |  |  |  |  |  |  | *p24* | NS |
|  |  |  |  |  |  |  |  |  | BR_SP_Agi07 | *p29* | NS |
|  |  |  |  |  |  |  |  |  |  | *p32* | MN955089 |
|  |  |  |  |  |  |  |  |  |  | *p24* | NS |
| 92-106 |  |  |  |  |  |  |  | CRD+SJP | BR_SP_Agi13 to Agi27 | *p29* | NS |
|  |  |  |  |  |  |  |  |  |  | *p32* | NS |
|  |  |  |  |  |  |  |  |  |  | *p24* | NS |
| 107 | 92 | *C. sinensis* (Valencia) | single | Fruit | Aguaí, SP, BR | commercial | 2017 | CRD+SJP | BR_SP_Agi08 | *p29* | MN954958 |
|  |  |  |  |  |  |  |  |  |  | *p32* | MN955090 |
|  |  |  |  |  |  |  |  |  |  | *p24* | NS |
|  |  |  |  |  |  |  |  |  | BR_SP_Agi09 | *p29* | MN954959 |
|  |  |  |  |  |  |  |  |  |  | *p32* | MN955091 |
|  |  |  |  |  |  |  |  |  |  | *p24* | NS |
|  |  |  |  |  |  |  |  |  | BR_SP_Agi10 | *p29* | MN954960 |
|  |  |  |  |  |  |  |  |  |  | *p32* | NS |
|  |  |  |  |  |  |  |  |  |  | *p24* | NS |
|  |  |  |  |  |  |  |  |  | BR_SP_Agi11 | *p29* | MN954961 |
|  |  |  |  |  |  |  |  |  |  | *p32* | NS |
|  |  |  |  |  |  |  |  |  |  | *p24* | NS |
|  |  |  |  |  |  |  |  |  | BR_SP_Agi12 | *p29* | NS |
|  |  |  |  |  |  |  |  |  |  | *p32* | NS |
|  |  |  |  |  |  |  |  |  |  | *p24* | NS |
| 108 | 93 | *C. sinensis* (Hamlin) | single | Fruit | Altinópolis, SP, BR | commercial | 2017 | SJP | BR_SP_Alt01 | *p29* | MN954962 |
|  |  |  |  |  |  |  |  |  |  | *p32* | MN955092 |
|  |  |  |  |  |  |  |  |  |  | *p24* | NS |
|  |  |  |  |  |  |  |  |  | BR_SP_Alt02 | *p29* | MN954963 |
|  |  |  |  |  |  |  |  |  |  | *p32* | MN955093 |
|  |  |  |  |  |  |  |  |  |  | *p24* | NS |
|  |  |  |  |  |  |  |  |  | BR_SP_Alt03 | *p29* | MN954964 |
|  |  |  |  |  |  |  |  |  |  | *p32* | MN955094 |
|  |  |  |  |  |  |  |  |  |  | *p24* | NS |
|  |  |  |  |  |  |  |  |  | BR_SP_Alt04 | *p29* | NS |
|  |  |  |  |  |  |  |  |  |  | *p32* | MN955095 |
|  |  |  |  |  |  |  |  |  |  | *p24* | NS |
|  |  |  |  |  |  |  |  |  | BR_SP_Alt05 | *p29* | NS |
|  |  |  |  |  |  |  |  |  |  | *p32* | MN955096 |
|  |  |  |  |  |  |  |  |  |  | *p24* | NS |
|  |  |  |  |  |  |  |  |  | BR_SP_Alt06 | *p29* | NS |
|  |  |  |  |  |  |  |  |  |  | *p32* | NS |
|  |  |  |  |  |  |  |  |  |  | *p24* | MN955097 |
|  |  |  |  |  |  |  |  |  | BR_SP_Alt07 | *p29* | NS |
|  |  |  |  |  |  |  |  |  |  | *p32* | NS |
|  |  |  |  |  |  |  |  |  |  | *p24* | NS |
| 109-123 |  |  | single |  |  |  |  | SJP | BR_SP_Alt08 to Alt22 | *p29* | NS |
|  |  |  |  |  |  |  |  |  |  | *p32* | NS |
|  |  |  |  |  |  |  |  |  |  | *p24* | NS |
| 124 | 94 | *C. sinensis* (New hall) | pool | Leaf | Argentina | non-commercial | 2017 | CRD | AR12 | *p29* | NS |
|  |  |  |  |  |  |  |  |  |  | *p32* | NS |
|  |  |  |  |  |  |  |  |  |  | *p24* | NS |
| 125 | 95 | *C. sinensis* (Hamlin) | single | Fruit | Barretos, SP, BR | commercial | 2017 | SJP | BR_SP_Bar01 | *p29* | MN954970 |
|  |  |  |  |  |  |  |  |  |  | *p32* | MN955100 |
|  |  |  |  |  |  |  |  |  |  | *p24* | NS |
|  |  |  |  |  |  |  |  |  | BR_SP_Bar02 | *p29* | MN954971 |
|  |  |  |  |  |  |  |  |  |  | *p32* | MN955101 |
|  |  |  |  |  |  |  |  |  |  | *p24* | NS |
|  |  |  |  |  |  |  |  |  | BR_SP_Bar03 | *p29* | MN954972 |
|  |  |  |  |  |  |  |  |  |  | *p32* | MN955102 |
|  |  |  |  |  |  |  |  |  |  | *p24* | NS |
|  |  |  |  |  |  |  |  |  | BR_SP_Bar04 | *p29* | MN954973 |
|  |  |  |  |  |  |  |  |  |  | *p32* | MN955103 |
|  |  |  |  |  |  |  |  |  |  | *p24* | NS |
|  |  |  |  |  |  |  |  |  | BR_SP_Bar05 | *p29* | MN954974 |
|  |  |  |  |  |  |  |  |  |  | *p32* | MN955104 |
|  |  |  |  |  |  |  |  |  |  | *p24* | NS |
|  |  |  |  |  |  |  |  |  | BR_SP_Bar06 | *p29* | NS |
|  |  |  |  |  |  |  |  |  |  | *p32* | MN955105 |
|  |  |  |  |  |  |  |  |  |  | *p24* | NS |
|  |  |  |  |  |  |  |  |  | BR_SP_Bar07 | *p29* | NS |
|  |  |  |  |  |  |  |  |  |  | *p32* | MN955106 |
|  |  |  |  |  |  |  |  |  |  | *p24* | NS |
| 126 |  |  | single |  |  |  |  | SJP | BR_SP_Bar08 | *p29* | MN954975 |
|  |  |  |  |  |  |  |  |  |  | *p32* | MN955107 |
|  |  |  |  |  |  |  |  |  |  | *p24* | NS |
|  |  |  |  |  |  |  |  |  | BR_SP_Bar09 | *p29* | MN954976 |
|  |  |  |  |  |  |  |  |  |  | *p32* | MN955108 |
|  |  |  |  |  |  |  |  |  |  | *p24* | NS |
|  |  |  |  |  |  |  |  |  | BR_SP_Bar10 | *p29* | MN954977 |
|  |  |  |  |  |  |  |  |  |  | *p32* | MN955109 |
|  |  |  |  |  |  |  |  |  |  | *p24* | NS |
|  |  |  |  |  |  |  |  |  | BR_SP_Bar11 | *p29* | MN954978 |
|  |  |  |  |  |  |  |  |  |  | *p32* | MN955110 |
|  |  |  |  |  |  |  |  |  |  | *p24* | NS |
|  |  |  |  |  |  |  |  |  | BR_SP_Bar12 | *p29* | MN954979 |
|  |  |  |  |  |  |  |  |  |  | *p32* | MN955111 |
|  |  |  |  |  |  |  |  |  |  | *p24* | NS |
|  |  |  |  |  |  |  |  |  | BR_SP_Bar13 | *p29* | NS |
|  |  |  |  |  |  |  |  |  |  | *p32* | MN955112 |
|  |  |  |  |  |  |  |  |  |  | *p24* | NS |
|  |  |  |  |  |  |  |  |  | BR_SP_Bar14 | *p29* | NS |
|  |  |  |  |  |  |  |  |  |  | *p32* | MN955113 |
|  |  |  |  |  |  |  |  |  |  | *p24* | NS |
|  |  |  |  |  |  |  |  |  | BR_SP_Bar15 | *p29* | NS |
|  |  |  |  |  |  |  |  |  |  | *p32* | MN955114 |
|  |  |  |  |  |  |  |  |  |  | *p24* | NS |
|  |  |  |  |  |  |  |  |  | BR_SP_Bar16 | *p29* | NS |
|  |  |  |  |  |  |  |  |  |  | *p32* | MN955115 |
|  |  |  |  |  |  |  |  |  |  | *p24* | NS |
| 127-128 |  |  | single |  |  |  |  | SJP | BR_SP_Bar17 and Bar18 | *p29* | NS |
|  |  |  |  |  |  |  |  |  |  | *p32* | NS |
|  |  |  |  |  |  |  |  |  |  | *p24* | NS |
| 129 | 96 | *C. sinensis* (Hamlin) | single  single | Fruit | Bebedouro, SP, BR | commercial | 2017 | SJP | BR_SP_Beb01 | *p29* | MN954980 |
|  |  |  |  |  |  |  |  |  |  | *p32* | MN955116 |
|  |  |  |  |  |  |  |  |  |  | *p24* | NS |
|  |  |  |  |  |  |  |  |  | BR_SP_Beb02 | *p29* | MN954981 |
|  |  |  |  |  |  |  |  |  |  | *p32* | MN955117 |
|  |  |  |  |  |  |  |  |  |  | *p24* | NS |
|  |  |  |  |  |  |  |  |  | BR_SP_Beb03 | *p29* | MN954982 |
|  |  |  |  |  |  |  |  |  |  | *p32* | MN955118 |
|  |  |  |  |  |  |  |  |  |  | *p24* | NS |
|  |  |  |  |  |  |  |  |  | BR_SP_Beb04 | *p29* | MN954983 |
|  |  |  |  |  |  |  |  |  |  | *p32* | MN955119 |
|  |  |  |  |  |  |  |  |  |  | *p24* | NS |
|  |  |  |  |  |  |  |  |  | BR_SP_Beb05 | *p29* | MN954984 |
|  |  |  |  |  |  |  |  |  |  | *p32* | MN955120 |
|  |  |  |  |  |  |  |  |  |  | *p24* | NS |
|  |  |  |  |  |  |  |  |  | BR_SP_Beb06 | *p29* | NS |
|  |  |  |  |  |  |  |  |  |  | *p32* | MN955121 |
|  |  |  |  |  |  |  |  |  |  | *p24* | NS |
|  |  |  |  |  |  |  |  |  | BR_SP_Beb07 | *p29* | NS |
|  |  |  |  |  |  |  |  |  |  | *p32* | MN955122 |
|  |  |  |  |  |  |  |  |  |  | *p24* | NS |
|  |  |  |  |  |  |  |  |  | BR_SP_Beb08 | *p29* | NS |
|  |  |  |  |  |  |  |  |  |  | *p32* | MN955123 |
|  |  |  |  |  |  |  |  |  |  | *p24* | NS |
|  |  |  |  |  |  |  |  |  | BR_SP_Beb09 | *p29* | NS |
|  |  |  |  |  |  |  |  |  |  | *p32* | MN955124 |
|  |  |  |  |  |  |  |  |  |  | *p24* | NS |
|  |  |  |  |  |  |  |  |  | BR_SP_Beb10 | *p29* | NS |
|  |  |  |  |  |  |  |  |  |  | *p32* | MN955125 |
|  |  |  |  |  |  |  |  |  |  | *p24* | NS |
|  |  |  |  |  |  |  |  |  | BR_SP_Beb11 | *p29* | NS |
|  |  |  |  |  |  |  |  |  |  | *p32* | MN955126 |
|  |  |  |  |  |  |  |  |  |  | *p24* | NS |
| 130 |  |  | single |  |  |  |  | SJP | BR_SP_Beb12 | *p29* | MN954985 |
|  |  |  |  |  |  |  |  |  |  | *p32* | MN955127 |
|  |  |  |  |  |  |  |  |  |  | *p24* | NS |
|  |  |  |  |  |  |  |  |  | BR_SP_Beb13 | *p29* | MN954986 |
|  |  |  |  |  |  |  |  |  |  | *p32* | MN955128 |
|  |  |  |  |  |  |  |  |  |  | *p24* | NS |
|  |  |  |  |  |  |  |  |  | BR_SP_Beb14 | *p29* | NS |
|  |  |  |  |  |  |  |  |  |  | *p32* | MN955129 |
|  |  |  |  |  |  |  |  |  |  | *p24* | NS |
|  |  |  |  |  |  |  |  |  | BR_SP_Beb15 | *p29* | NS |
|  |  |  |  |  |  |  |  |  |  | *p32* | MN955130 |
|  |  |  |  |  |  |  |  |  |  | *p24* | NS |
|  |  |  |  |  |  |  |  |  | BR_SP_Beb16 | *p29* | NS |
|  |  |  |  |  |  |  |  |  |  | *p32* | MN955131 |
|  |  |  |  |  |  |  |  |  |  | *p24* | NS |
|  |  |  |  |  |  |  |  |  | BR_SP_Beb17 | *p29* | NS |
|  |  |  |  |  |  |  |  |  |  | *p32* | MN955132 |
|  |  |  |  |  |  |  |  |  |  | *p24* | NS |
|  |  |  |  |  |  |  |  |  | BR_SP_Beb18 | *p29* | NS |
|  |  |  |  |  |  |  |  |  |  | *p32* | MN955133 |
|  |  |  |  |  |  |  |  |  |  | *p24* | NS |
|  |  |  |  |  |  |  |  |  | BR_SP_Beb19 | *p29* | NS |
|  |  |  |  |  |  |  |  |  |  | *p32* | MN955134 |
|  |  |  |  |  |  |  |  |  |  | *p24* | NS |
|  |  |  |  |  |  |  |  |  | BR_SP_Beb20 | *p29* | NS |
|  |  |  |  |  |  |  |  |  |  | *p32* | MN955135 |
|  |  |  |  |  |  |  |  |  |  | *p24* | NS |
| 131 |  |  | single |  |  |  |  | SJP | BR_SP_Beb21 | *p29* | NS |
|  |  |  |  |  |  |  |  |  |  | *p32* | MN955136 |
|  |  |  |  |  |  |  |  |  |  | *p24* | NS |
|  |  |  |  |  |  |  |  |  | BR_SP_Beb22 | *p29* | NS |
|  |  |  |  |  |  |  |  |  |  | *p32* | MN955137 |
|  |  |  |  |  |  |  |  |  |  | *p24* | NS |
|  |  |  |  |  |  |  |  |  | BR_SP_Beb23 | *p29* | NS |
|  |  |  |  |  |  |  |  |  |  | *p32* | MN955138 |
|  |  |  |  |  |  |  |  |  |  | *p24* | NS |
|  |  |  |  |  |  |  |  |  | BR_SP_Beb24 | *p29* | NS |
|  |  |  |  |  |  |  |  |  |  | *p32* | MN955139 |
|  |  |  |  |  |  |  |  |  |  | *p24* | NS |
|  |  |  |  |  |  |  |  |  | BR_SP_Beb25 | *p29* | NS |
|  |  |  |  |  |  |  |  |  |  | *p32* | MN955140 |
|  |  |  |  |  |  |  |  |  |  | *p24* | NS |
|  |  |  |  |  |  |  |  |  | BR_SP_Beb26 | *p29* | NS |
|  |  |  |  |  |  |  |  |  |  | *p32* | MN955141 |
|  |  |  |  |  |  |  |  |  |  | *p24* | NS |
|  |  |  |  |  |  |  |  |  | BR_SP_Beb27 | *p29* | NS |
|  |  |  |  |  |  |  |  |  |  | *p32* | MN955142 |
|  |  |  |  |  |  |  |  |  |  | *p24* | NS |
|  |  |  |  |  |  |  |  |  | BR_SP_Beb28 | *p29* | NS |
|  |  |  |  |  |  |  |  |  |  | *p32* | MN955143 |
|  |  |  |  |  |  |  |  |  |  | *p24* | NS |
|  |  |  |  |  |  |  |  |  | BR_SP_Beb29 | *p29* | NS |
|  |  |  |  |  |  |  |  |  |  | *p32* | MN955144 |
|  |  |  |  |  |  |  |  |  |  | *p24* | NS |
|  |  |  |  |  |  |  |  |  | BR_SP_Beb30 | *p29* | NS |
|  |  |  |  |  |  |  |  |  |  | *p32* | MN955145 |
|  |  |  |  |  |  |  |  |  |  | *p24* | NS |
|  |  |  |  |  |  |  |  |  | BR_SP_Beb31 | *p29* | NS |
|  |  |  |  |  |  |  |  |  |  | *p32* | MN955146 |
|  |  |  |  |  |  |  |  |  |  | *p24* | NS |
| 132 | 97 | *C. sinensis* (Valencia) | pool | Leaf | Bella Vista, Corrientes, AR | non-commercial | 2017 | CRD | AR05 | RNA1 | MT554528 |
|  |  |  |  |  |  |  |  |  |  | RNA2 | MT554542 |
| 133 | 98 | *C. sinensis* (Pera) | single | Fruit | Brotas, SP, BR | commercial | 2017 | SJP | BR_SP_Bro01 | *p29* | MN954989 |
|  |  |  |  |  |  |  |  |  |  | *p32* | MN955147 |
|  |  |  |  |  |  |  |  |  |  | *p24* | NS |
|  |  |  |  |  |  |  |  |  | BR_SP_Bro02 | *p29* | MN954990 |
|  |  |  |  |  |  |  |  |  |  | *p32* | MN955148 |
|  |  |  |  |  |  |  |  |  |  | *p24* | NS |
|  |  |  |  |  |  |  |  |  | BR_SP_Bro03 | *p29* | MN954991 |
|  |  |  |  |  |  |  |  |  |  | *p32* | MN955149 |
|  |  |  |  |  |  |  |  |  |  | *p24* | NS |
|  |  |  |  |  |  |  |  |  | BR_SP_Bro04 | *p29* | MN954992 |
|  |  |  |  |  |  |  |  |  |  | *p32* | NS |
|  |  |  |  |  |  |  |  |  |  | *p24* | NS |
| 134 |  |  | single |  |  |  |  | SJP | BR_SP_Bro12 | *p29* | NS |
|  |  |  |  |  |  |  |  |  |  | *p32* | NS |
|  |  |  |  |  |  |  |  |  |  | *p24* | NS |
| 135 | 99 | *C. sinensis* (Natal) | single | Fruit | Brotas, SP, BR | commercial | 2017 | SJP | BR_SP_Bro05 | *p29* | MN954993 |
|  |  |  |  |  |  |  |  |  |  | *p32* | MN955150 |
|  |  |  |  |  |  |  |  |  |  | *p24* | NS |
|  |  |  |  |  |  |  |  |  | BR_SP_Bro06 | *p29* | MN954994 |
|  |  |  |  |  |  |  |  |  |  | *p32* | MN955151 |
|  |  |  |  |  |  |  |  |  |  | *p24* | NS |
|  |  |  |  |  |  |  |  |  | BR_SP_Bro07 | *p29* | MN954995 |
|  |  |  |  |  |  |  |  |  |  | *p32* | MN955152 |
|  |  |  |  |  |  |  |  |  |  | *p24* | NS |
|  |  |  |  |  |  |  |  |  | BR_SP_Bro08 | *p29* | NS |
|  |  |  |  |  |  |  |  |  |  | *p32* | MN955153 |
|  |  |  |  |  |  |  |  |  |  | *p24* | NS |
|  |  |  |  |  |  |  |  |  | BR_SP_Bro09 | *p29* | NS |
|  |  |  |  |  |  |  |  |  |  | *p32* | MN955154 |
|  |  |  |  |  |  |  |  |  |  | *p24* | NS |
|  |  |  |  |  |  |  |  |  | BR_SP_Bro10 | *p29* | NS |
|  |  |  |  |  |  |  |  |  |  | *p32* | MN955155 |
|  |  |  |  |  |  |  |  |  |  | *p24* | NS |
|  |  |  |  |  |  |  |  |  | BR_SP_Bro11 | *p29* | NS |
|  |  |  |  |  |  |  |  |  |  | *p32* | MN955154 |
|  |  |  |  |  |  |  |  |  |  | *p24* | NS |
|  |  |  |  |  |  |  |  |  | BR_SP_Bro11 | *p29* | NS |
|  |  |  |  |  |  |  |  |  |  | *p32* | MN955157 |
|  |  |  |  |  |  |  |  |  |  | *p24* | NS |
| 136-137 |  |  | single |  |  |  |  | SJP | BR_SP_Bro13 and Bro14 | *p29* | NS |
|  |  |  |  |  |  |  |  |  |  | *p32* | NS |
|  |  |  |  |  |  |  |  |  |  | *p24* | NS |
| 138 | 100 | *C. sinensis* (Natal) | single | Fruit | Cafelândia, SP, BR | commercial | 2017 | SJP | BR_SP_Cfl01 | *p29* | NS |
|  |  |  |  |  |  |  |  |  |  | *p32* | NS |
|  |  |  |  |  |  |  |  |  |  | *p24* | NS |
| 139 | 101 | *C. sinensis* (Natal) | single | Fruit | Cerqueira Cesar, SP, BR | commercial | 2017 | SJP | BR_SP_CrC01 | *p29* | MN954996 |
|  |  |  |  |  |  |  |  |  |  | *p32* | MN955158 |
|  |  |  |  |  |  |  |  |  |  | *p24* | NS |
|  |  |  |  |  |  |  |  |  | BR_SP_CrC02 | *p29* | MN954997 |
|  |  |  |  |  |  |  |  |  |  | *p32* | MN955159 |
|  |  |  |  |  |  |  |  |  |  | *p24* | NS |
|  |  |  |  |  |  |  |  |  | BR_SP_CrC03 | *p29* | MN954998 |
|  |  |  |  |  |  |  |  |  |  | *p32* | MN955160 |
|  |  |  |  |  |  |  |  |  |  | *p24* | NS |
|  |  |  |  |  |  |  |  |  | BR_SP_CrC04 | *p29* | MN954999 |
|  |  |  |  |  |  |  |  |  |  | *p32* | MN955161 |
|  |  |  |  |  |  |  |  |  |  | *p24* | NS |
|  |  |  |  |  |  |  |  |  | BR_SP_CrC05 | *p29* | NS |
|  |  |  |  |  |  |  |  |  |  | *p32* | NS |
|  |  |  |  |  |  |  |  |  |  | *p24* | NS |
|  |  |  |  |  |  |  |  |  | BR_SP_CrC06 | *p29* | MN955000 |
|  |  |  |  |  |  |  |  |  |  | *p32* | MN955162 |
|  |  |  |  |  |  |  |  |  |  | *p24* | NS |
|  |  |  |  |  |  |  |  |  | BR_SP_CrC07 | *p29* | NS |
|  |  |  |  |  |  |  |  |  |  | *p32* | MN955163 |
|  |  |  |  |  |  |  |  |  |  | *p24* | NS |
|  |  |  |  |  |  |  |  |  | BR_SP_CrC08 | *p29* | NS |
|  |  |  |  |  |  |  |  |  |  | *p32* | MN955164 |
|  |  |  |  |  |  |  |  |  |  | *p24* | NS |
| 140 |  |  | single |  |  |  |  | SJP | BR_SP_CrC09 | *p29* | MN955001 |
|  |  |  |  |  |  |  |  |  |  | *p32* | MN955165 |
|  |  |  |  |  |  |  |  |  |  | *p24* | NS |
|  |  |  |  |  |  |  |  |  | BR_SP_CrC10 | *p29* | MN955002 |
|  |  |  |  |  |  |  |  |  |  | *p32* | MN955166 |
|  |  |  |  |  |  |  |  |  |  | *p24* | NS |
|  |  |  |  |  |  |  |  |  | BR_SP_CrC11 | *p29* | MN955003 |
|  |  |  |  |  |  |  |  |  |  | *p32* | MN955167 |
|  |  |  |  |  |  |  |  |  |  | *p24* | NS |
|  |  |  |  |  |  |  |  |  | BR_SP_CrC12 | *p29* | NS |
|  |  |  |  |  |  |  |  |  |  | *p32* | MN955168 |
|  |  |  |  |  |  |  |  |  |  | *p24* | NS |
| 141-154 |  |  | single |  |  |  |  | SJP | BR_SP_CrC13 to CrC28 | *p29* | NS |
|  |  |  |  |  |  |  |  |  |  | *p32* | NS |
|  |  |  |  |  |  |  |  |  |  | *p24* | NS |
| 155 | 102 | *C. sinensis* (Hamlin) | single | Fruit | Colômbia, SP, BR | commercial | 2017 | SJP | BR_SP_Clb01 | *p29* | MN955005 |
|  |  |  |  |  |  |  |  |  |  | *p32* | NS |
|  |  |  |  |  |  |  |  |  |  | *p24* | NS |
|  |  |  |  |  |  |  |  |  | BR_SP_Clb02 | *p29* | MN955006 |
|  |  |  |  |  |  |  |  |  |  | *p32* | NS |
|  |  |  |  |  |  |  |  |  |  | *p24* | NS |
|  |  |  |  |  |  |  |  |  | BR_SP_Clb03 | *p29* | MN955007 |
|  |  |  |  |  |  |  |  |  |  | *p32* | NS |
|  |  |  |  |  |  |  |  |  |  | *p24* | NS |
|  |  |  |  |  |  |  |  |  | BR_SP_Clb04 | *p29* | MN955008 |
|  |  |  |  |  |  |  |  |  |  | *p32* | NS |
|  |  |  |  |  |  |  |  |  |  | *p24* | NS |
|  |  |  |  |  |  |  |  |  | BR_SP_Clb05 | *p29* | MN955009 |
|  |  |  |  |  |  |  |  |  |  | *p32* | NS |
|  |  |  |  |  |  |  |  |  |  | *p24* | NS |
| 156-159 |  |  | single |  |  |  | 2017 | SJP | BR_SP_Clb06 to Clb09 | *p29* | NS |
|  |  |  |  |  |  |  |  |  |  | *p32* | NS |
|  |  |  |  |  |  |  |  |  |  | *p24* | NS |
| 160 | 103 | *C. sinensis* (Pera) | single | Fruit | Guaimbê, SP, BR | commercial | 2017 | SJP | BR_SP_Gua01 | *p29* | MN955015 |
|  |  |  |  |  |  |  |  |  |  | *p32* | MN955169 |
|  |  |  |  |  |  |  |  |  |  | *p24* | NS |
|  |  |  |  |  |  |  |  |  | BR_SP_Gua02 | *p29* | MN955016 |
|  |  |  |  |  |  |  |  |  |  | *p32* | MN955170 |
|  |  |  |  |  |  |  |  |  |  | *p24* | NS |
|  |  |  |  |  |  |  |  |  | BR_SP_Gua03 | *p29* | NS |
|  |  |  |  |  |  |  |  |  |  | *p32* | MN955171 |
|  |  |  |  |  |  |  |  |  |  | *p24* | NS |
|  |  |  |  |  |  |  |  |  | BR_SP_Gua04 | *p29* | NS |
|  |  |  |  |  |  |  |  |  |  | *p32* | MN955172 |
|  |  |  |  |  |  |  |  |  |  | *p24* | NS |
|  |  |  |  |  |  |  |  |  | BR_SP_Gua05 | *p29* | NS |
|  |  |  |  |  |  |  |  |  |  | *p32* | MN955173 |
|  |  |  |  |  |  |  |  |  |  | *p24* | NS |
|  |  |  |  |  |  |  |  |  | BR_SP_Gua06 | *p29* | NS |
|  |  |  |  |  |  |  |  |  |  | *p32* | MN955174 |
|  |  |  |  |  |  |  |  |  |  | *p24* | NS |
|  |  |  |  |  |  |  |  |  | BR_SP_Gua07 | *p29* | NS |
|  |  |  |  |  |  |  |  |  |  | *p32* | MN955175 |
|  |  |  |  |  |  |  |  |  |  | *p24* | NS |
|  |  |  |  |  |  |  |  |  | BR_SP_Gua08 | *p29* | NS |
|  |  |  |  |  |  |  |  |  |  | *p32* | MN955176 |
|  |  |  |  |  |  |  |  |  |  | *p24* | NS |
| 161-182 |  |  | single |  |  |  | 2017 | SJP | BR_SP_Gua09 to Gua30 | *p29* | NS |
|  |  |  |  |  |  |  |  |  |  | *p32* | NS |
|  |  |  |  |  |  |  |  |  |  | *p24* | NS |
| 183 | 104 | *C. sinensis* (Pera) | single | Fruit | Guapiaçu, SP, BR | commercial | 2017 | SJP  SJP | BR_SP_Gup01 | *p29* | NS |
|  |  |  |  |  |  |  |  |  |  | *p32* | MN955177 |
|  |  |  |  |  |  |  |  |  |  | *p24* | NS |
|  |  |  |  |  |  |  |  |  | BR_SP_Gup02 | *p29* | NS |
|  |  |  |  |  |  |  |  |  |  | *p32* | MN955178 |
|  |  |  |  |  |  |  |  |  |  | *p24* | NS |
|  |  |  |  |  |  |  |  |  | BR_SP_Gup03 | *p29* | NS |
|  |  |  |  |  |  |  |  |  |  | *p32* | MN955179 |
|  |  |  |  |  |  |  |  |  |  | *p24* | NS |
|  |  |  |  |  |  |  |  |  | BR_SP_Gup04 | *p29* | NS |
|  |  |  |  |  |  |  |  |  |  | *p32* | MN955180 |
|  |  |  |  |  |  |  |  |  |  | *p24* | NS |
|  |  |  |  |  |  |  |  |  | BR_SP_Gup05 | *p29* | NS |
|  |  |  |  |  |  |  |  |  |  | *p32* | MN955181 |
|  |  |  |  |  |  |  |  |  |  | *p24* | NS |
|  |  |  |  |  |  |  |  |  | BR_SP_Gup06 | *p29* | NS |
|  |  |  |  |  |  |  |  |  |  | *p32* | MN955182 |
|  |  |  |  |  |  |  |  |  |  | *p24* | NS |
|  |  |  |  |  |  |  |  |  | BR_SP_Gup07 | *p29* | NS |
|  |  |  |  |  |  |  |  |  |  | *p32* | MN955183 |
|  |  |  |  |  |  |  |  |  |  | *p24* | NS |
|  |  |  |  |  |  |  |  |  | BR_SP_Gup08 | *p29* | NS |
|  |  |  |  |  |  |  |  |  |  | *p32* | MN955184 |
|  |  |  |  |  |  |  |  |  |  | *p24* | NS |
|  |  |  |  |  |  |  |  |  | BR_SP_Gup09 | *p29* | NS |
|  |  |  |  |  |  |  |  |  |  | *p32* | MN955185 |
|  |  |  |  |  |  |  |  |  |  | *p24* | NS |
|  |  |  |  |  |  |  |  |  | BR_SP_Gup10 | *p29* | NS |
|  |  |  |  |  |  |  |  |  |  | *p32* | MN955186 |
|  |  |  |  |  |  |  |  |  |  | *p24* | NS |
|  |  |  |  |  |  |  |  |  | BR_SP_Gup11 | *p29* | NS |
|  |  |  |  |  |  |  |  |  |  | *p32* | MN955187 |
|  |  |  |  |  |  |  |  |  |  | *p24* | NS |
|  |  |  |  |  |  |  |  |  | BR_SP_Gup12 | *p29* | NS |
|  |  |  |  |  |  |  |  |  |  | *p32* | MN955188 |
|  |  |  |  |  |  |  |  |  |  | *p24* | NS |
| 184 |  |  | single |  |  |  |  | SJP | BR_SP_Gup13 | *p29* | NS |
|  |  |  |  |  |  |  |  |  |  | *p32* | MN955189 |
|  |  |  |  |  |  |  |  |  |  | *p24* | NS |
|  |  |  |  |  |  |  |  |  | BR_SP_Gup14 | *p29* | NS |
|  |  |  |  |  |  |  |  |  |  | *p32* | MN955190 |
|  |  |  |  |  |  |  |  |  |  | *p24* | NS |
|  |  |  |  |  |  |  |  |  | BR_SP_Gup15 | *p29* | NS |
|  |  |  |  |  |  |  |  |  |  | *p32* | MN955191 |
|  |  |  |  |  |  |  |  |  |  | *p24* | NS |
|  |  |  |  |  |  |  |  |  | BR_SP_Gup16 | *p29* | NS |
|  |  |  |  |  |  |  |  |  |  | *p32* | MN955192 |
|  |  |  |  |  |  |  |  |  |  | *p24* | NS |
|  |  |  |  |  |  |  |  |  | BR_SP_Gup17 | *p29* | NS |
|  |  |  |  |  |  |  |  |  |  | *p32* | MN955193 |
|  |  |  |  |  |  |  |  |  |  | *p24* | NS |
|  |  |  |  |  |  |  |  |  | BR_SP_Gup18 | *p29* | NS |
|  |  |  |  |  |  |  |  |  |  | *p32* | MN955194 |
|  |  |  |  |  |  |  |  |  |  | *p24* | NS |
| 185 | 105 | *C. sinensis* (Valencia) | single | Fruit | Leme, SP, BR | commercial | 2017 | SJP | BR_SP_Lem01 | *p29* | NS |
|  |  |  |  |  |  |  |  |  |  | *p32* | NS |
|  |  |  |  |  |  |  |  |  |  | *p24* | NS |
| 186 | 106 | *C. sinensis* (Hamlin) | single | Fruit | Mogi Mirim, SP, BR | commercial | 2017 | CRD+SJP | BR_SP_MgM01 | *p29* | MN955021 |
|  |  |  |  |  |  |  |  |  |  | *p32* | MN955197 |
|  |  |  |  |  |  |  |  |  |  | *p24* | NS |
|  |  |  |  |  |  |  |  |  | BR_SP_MgM02 | *p29* | MN955022 |
|  |  |  |  |  |  |  |  |  |  | *p32* | MN955198 |
|  |  |  |  |  |  |  |  |  |  | *p24* | NS |
|  |  |  |  |  |  |  |  |  | BR_SP_MgM03 | *p29* | MN955023 |
|  |  |  |  |  |  |  |  |  |  | *p32* | MN955199 |
|  |  |  |  |  |  |  |  |  |  | *p24* | NS |
|  |  |  |  |  |  |  |  |  | BR_SP_MgM04 | *p29* | MN955024 |
|  |  |  |  |  |  |  |  |  |  | *p32* | MN955200 |
|  |  |  |  |  |  |  |  |  |  | *p24* | NS |
|  |  |  |  |  |  |  |  |  | BR_SP_MgM05 | *p29* | MN955025 |
|  |  |  |  |  |  |  |  |  |  | *p32* | MN955201 |
|  |  |  |  |  |  |  |  |  |  | *p24* | NS |
|  |  |  |  |  |  |  |  |  | BR_SP_MgM06 | *p29* | MN955026 |
|  |  |  |  |  |  |  |  |  |  | *p32* | MN955202 |
|  |  |  |  |  |  |  |  |  |  | *p24* | NS |
|  |  |  |  |  |  |  |  |  | BR_SP_MgM07 | *p29* | MN955027 |
|  |  |  |  |  |  |  |  |  |  | *p32* | MN955203 |
|  |  |  |  |  |  |  |  |  |  | *p24* | NS |
|  |  |  |  |  |  |  |  |  | BR_SP_MgM08 | *p29* | NS |
|  |  |  |  |  |  |  |  |  |  | *p32* | MN955204 |
|  |  |  |  |  |  |  |  |  |  | *p24* | NS |
|  |  |  |  |  |  |  |  |  | BR_SP_MgM09 | *p29* | NS |
|  |  |  |  |  |  |  |  |  |  | *p32* | MN955205 |
|  |  |  |  |  |  |  |  |  |  | *p24* | NS |
|  |  |  |  |  |  |  |  |  | BR_SP_MgM10 | *p29* | NS |
|  |  |  |  |  |  |  |  |  |  | *p32* | MN955206 |
|  |  |  |  |  |  |  |  |  |  | *p24* | NS |
| 187 |  |  | single |  |  |  |  | SJP | BR_SP_MgM11 | *p29* | MN955028 |
|  |  |  |  |  |  |  |  |  |  | *p32* | MN955207 |
|  |  |  |  |  |  |  |  |  |  | *p24* | NS |
|  |  |  |  |  |  |  |  |  | BR_SP_MgM12 | *p29* | MN955029 |
|  |  |  |  |  |  |  |  |  |  | *p32* | MN955208 |
|  |  |  |  |  |  |  |  |  |  | *p24* | NS |
|  |  |  |  |  |  |  |  |  | BR_SP_MgM13 | *p29* | MN955030 |
|  |  |  |  |  |  |  |  |  |  | *p32* | MN955209 |
|  |  |  |  |  |  |  |  |  |  | *p24* | NS |
|  |  |  |  |  |  |  |  |  | BR_SP_MgM14 | *p29* | MN955031 |
|  |  |  |  |  |  |  |  |  |  | *p32* | MN955210 |
|  |  |  |  |  |  |  |  |  |  | *p24* | NS |
|  |  |  |  |  |  |  |  |  | BR_SP_MgM15 | *p29* | MN955032 |
|  |  |  |  |  |  |  |  |  |  | *p32* | MN955211 |
|  |  |  |  |  |  |  |  |  |  | *p24* | NS |
|  |  |  |  |  |  |  |  |  | BR_SP_MgM16 | *p29* | NS |
|  |  |  |  |  |  |  |  |  |  | *p32* | MN955212 |
|  |  |  |  |  |  |  |  |  |  | *p24* | NS |
|  |  |  |  |  |  |  |  |  | BR_SP_MgM17 | *p29* | NS |
|  |  |  |  |  |  |  |  |  |  | *p32* | MN955213 |
|  |  |  |  |  |  |  |  |  |  | *p24* | NS |
|  |  |  |  |  |  |  |  |  | BR_SP_MgM18 | *p29* | NS |
|  |  |  |  |  |  |  |  |  |  | *p32* | MN955214 |
|  |  |  |  |  |  |  |  |  |  | *p24* | NS |
|  |  |  |  |  |  |  |  |  | BR_SP_MgM19 | *p29* | NS |
|  |  |  |  |  |  |  |  |  |  | *p32* | MN955215 |
|  |  |  |  |  |  |  |  |  |  | *p24* | NS |
|  |  |  |  |  |  |  |  |  | BR_SP_MgM20 | *p29* | NS |
|  |  |  |  |  |  |  |  |  |  | *p32* | MN955216 |
|  |  |  |  |  |  |  |  |  |  | *p24* | NS |
| 188-194 |  |  | single |  |  |  |  | SJP or CRD+SJP | BR_SP_MgM31 to MgM37 | *p29* | NS |
|  |  |  |  |  |  |  |  |  |  | *p32* | NS |
|  |  |  |  |  |  |  |  |  |  | *p24* | NS |
| 195 | 107 | *C. sinensis* (Pera) | single | Fruit | Mogi Mirim, SP, BR | commercial | 2017 | CRD | BR_SP_MgM21 | *p29* | NS |
|  |  |  |  |  |  |  |  |  |  | *p32* | MN955217 |
|  |  |  |  |  |  |  |  |  |  | *p24* | NS |
|  |  |  |  |  |  |  |  |  | BR_SP_MgM22 | *p29* | NS |
|  |  |  |  |  |  |  |  |  |  | *p32* | MN955218 |
|  |  |  |  |  |  |  |  |  |  | *p24* | NS |
|  |  |  |  |  |  |  |  |  | BR_SP_MgM23 | *p29* | NS |
|  |  |  |  |  |  |  |  |  |  | *p32* | MN955219 |
|  |  |  |  |  |  |  |  |  |  | *p24* | NS |
|  |  |  |  |  |  |  |  |  | BR_SP_MgM24 | *p29* | NS |
|  |  |  |  |  |  |  |  |  |  | *p32* | MN955220 |
|  |  |  |  |  |  |  |  |  |  | *p24* | NS |
|  |  |  |  |  |  |  |  |  | BR_SP_MgM25 | *p29* | NS |
|  |  |  |  |  |  |  |  |  |  | *p32* | MN955221 |
|  |  |  |  |  |  |  |  |  |  | *p24* | NS |
|  |  |  |  |  |  |  |  |  | BR_SP_MgM26 | *p29* | NS |
|  |  |  |  |  |  |  |  |  |  | *p32* | MN955222 |
|  |  |  |  |  |  |  |  |  |  | *p24* | NS |
|  |  |  |  |  |  |  |  |  | BR_SP_MgM27 | *p29* | NS |
|  |  |  |  |  |  |  |  |  |  | *p32* | MN955223 |
|  |  |  |  |  |  |  |  |  |  | *p24* | NS |
|  |  |  |  |  |  |  |  |  | sBR_SP_MgM28 | *p29* | NS |
|  |  |  |  |  |  |  |  |  |  | *p32* | MN955224 |
|  |  |  |  |  |  |  |  |  |  | *p24* | NS |
|  |  |  |  |  |  |  |  |  | BR_SP_MgM29 | *p29* | NS |
|  |  |  |  |  |  |  |  |  |  | *p32* | MN955225 |
|  |  |  |  |  |  |  |  |  |  | *p24* | NS |
|  |  |  |  |  |  |  |  |  | BR_SP_MgM30 | *p29* | NS |
|  |  |  |  |  |  |  |  |  |  | *p32* | MN955226 |
|  |  |  |  |  |  |  |  |  |  | *p24* | NS |
| 196-198 |  |  | single |  |  |  |  | CRD or SJP | BR_SP_MgM38 to MgM40 | *p29* | NS |
|  |  |  |  |  |  |  |  |  |  | *p32* | NS |
|  |  |  |  |  |  |  |  |  |  | *p24* | NS |
| 199 | 108 | *C. sinensis* | pool | Leaf | Piracicaba, SP, BR | non-commercial | 2017 | CRD | BR_SP_Prb07 | *p29* | NS |
|  |  |  |  |  |  |  |  |  |  | *p32* | NS |
|  |  |  |  |  |  |  |  |  |  | *p24* | NS |
| 200 | 109 | *C. sinensis* (Westin) | single | Fruit | Pirassununga, SP, BR | commercial | 2017 | SJP | BR_SP_Prg01 | *p29* | MN955035 |
|  |  |  |  |  |  |  |  |  |  | *p32* | MN955229 |
|  |  |  |  |  |  |  |  |  |  | *p24* |  |
|  |  |  |  |  |  |  |  |  | BR_SP_Prg02 | *p29* | MN955036 |
|  |  |  |  |  |  |  |  |  |  | *p32* | MN955230 |
|  |  |  |  |  |  |  |  |  |  | *p24* | NS |
|  |  |  |  |  |  |  |  |  | BR_SP_Prg03 | *p29* | MN955037 |
|  |  |  |  |  |  |  |  |  |  | *p32* | NS |
|  |  |  |  |  |  |  |  |  |  | *p24* | NS |
| 201 |  |  | single |  |  |  |  |  | BR_SP_Prg04 | *p29* | NS |
|  |  |  |  |  |  |  |  |  |  | *p32* | MN955231 |
|  |  |  |  |  |  |  |  |  |  | *p24* | NS |
|  |  |  |  |  |  |  |  |  | BR_SP_Prg05 | *p29* | NS |
|  |  |  |  |  |  |  |  |  |  | *p32* | MN955232 |
|  |  |  |  |  |  |  |  |  |  | *p24* | NS |
| 202 |  |  | single |  |  |  |  | SJP | BR_SP_Prg05 | *p29* | NS |
|  |  |  |  |  |  |  |  |  |  | *p32* | NS |
|  |  |  |  |  |  |  |  |  |  | *p24* | NS |
| 203 | 110 | *C. sinensis* (Valencia) | single | Fruit | Pongaí, SP, BR | commercial | 2017 | CRD+SJP | BR_SP_Png01 | *p29* | NS |
|  |  |  |  |  |  |  |  |  |  | *p32* | MN955233 |
|  |  |  |  |  |  |  |  |  |  | *p24* | NS |
|  |  |  |  |  |  |  |  |  | BR_SP_Png02 | *p29* | NS |
|  |  |  |  |  |  |  |  |  |  | *p32* | MN955234 |
|  |  |  |  |  |  |  |  |  |  | *p24* | NS |
|  |  |  |  |  |  |  |  |  | BR_SP_Png03 | *p29* | NS |
|  |  |  |  |  |  |  |  |  |  | *p32* | MN955235 |
|  |  |  |  |  |  |  |  |  |  | *p24* | NS |
|  |  |  |  |  |  |  |  |  | BR_SP_Png04 | *p29* | NS |
|  |  |  |  |  |  |  |  |  |  | *p32* | MN955236 |
|  |  |  |  |  |  |  |  |  |  | *p24* | NS |
|  |  |  |  |  |  |  |  |  | BR_SP_Png05 | *p29* | NS |
|  |  |  |  |  |  |  |  |  |  | *p32* | MN955237 |
|  |  |  |  |  |  |  |  |  |  | *p24* | NS |
|  |  |  |  |  |  |  |  |  | BR_SP_Png06 | *p29* | NS |
|  |  |  |  |  |  |  |  |  |  | *p32* | MN955238 |
|  |  |  |  |  |  |  |  |  |  | *p24* | NS |
|  |  |  |  |  |  |  |  |  | BR_SP_Png07 | *p29* | NS |
|  |  |  |  |  |  |  |  |  |  | *p32* | MN955239 |
|  |  |  |  |  |  |  |  |  |  | *p24* | NS |
|  |  |  |  |  |  |  |  |  | BR_SP_Png08 | *p29* | NS |
|  |  |  |  |  |  |  |  |  |  | *p32* | MN955240 |
|  |  |  |  |  |  |  |  |  |  | *p24* | NS |
| 204 |  |  | single |  |  |  |  | CRD+SJP | BR_SP_Png09 | *p29* | NS |
|  |  |  |  |  |  |  |  |  |  | *p32* | MN955241 |
|  |  |  |  |  |  |  |  |  |  | *p24* | NS |
|  |  |  |  |  |  |  |  |  | BR_SP_Png10 | *p29* | NS |
|  |  |  |  |  |  |  |  |  |  | *p32* | MN955242 |
|  |  |  |  |  |  |  |  |  |  | *p24* | NS |
|  |  |  |  |  |  |  |  |  | BR_SP_Png11 | *p29* | NS |
|  |  |  |  |  |  |  |  |  |  | *p32* | MN955243 |
|  |  |  |  |  |  |  |  |  |  | *p24* | NS |
| 205 | 111 | *C. sinensis* (Valencia) | single | Fruit | Santa Maria da Serra, SP, BR | commercial | 2017 | SJP | BR_SP_SSr01 | *p29* | MN955039 |
|  |  |  |  |  |  |  |  |  |  | *p32* | MN955244 |
|  |  |  |  |  |  |  |  |  |  | *p24* | NS |
|  |  |  |  |  |  |  |  |  | BR_SP_ SSr 02 | *p29* | MN955040 |
|  |  |  |  |  |  |  |  |  |  | *p32* | MN955245 |
|  |  |  |  |  |  |  |  |  |  | *p24* | NS |
|  |  |  |  |  |  |  |  |  | BR_SP_ SSr 03 | *p29* | MN955041 |
|  |  |  |  |  |  |  |  |  |  | *p32* | NS |
|  |  |  |  |  |  |  |  |  |  | *p24* | NS |
|  |  |  |  |  |  |  |  |  | BR_SP_ SSr04 | *p29* | MN955042 |
|  |  |  |  |  |  |  |  |  |  | *p32* | NS |
|  |  |  |  |  |  |  |  |  |  | *p24* | NS |
|  |  |  |  |  |  |  |  |  | BR_SP_ SSr05 | *p29* | MN955043 |
|  |  |  |  |  |  |  |  |  |  | *p32* | NS |
|  |  |  |  |  |  |  |  |  |  | *p24* | NS |
|  |  |  |  |  |  |  |  |  | BR_SP_ SSr06 | *p29* | MN955044 |
|  |  |  |  |  |  |  |  |  |  | *p32* | NS |
|  |  |  |  |  |  |  |  |  |  | *p24* | NS |
|  |  |  |  |  |  |  |  |  | BR_SP_ SSr 07 | *p29* | MN955045 |
|  |  |  |  |  |  |  |  |  |  | *p32* | NS |
|  |  |  |  |  |  |  |  |  |  | *p24* | NS |
| 206 | 112 | *C. sinensis* | single | Fruit | São Paulo, BR | commercial | 2017 | SJP | BR_SP01 | *p29* | NS |
|  |  |  |  |  |  |  |  |  |  | *p32* | MN955246 |
|  |  |  |  |  |  |  |  |  |  | *p24* | NS |
|  |  |  |  |  |  |  |  |  | BR_SP02 | *p29* | NS |
|  |  |  |  |  |  |  |  |  |  | *p32* | MN955247 |
|  |  |  |  |  |  |  |  |  |  | *p24* | NS |
|  |  |  |  |  |  |  |  |  | BR_SP03 | *p29* | NS |
|  |  |  |  |  |  |  |  |  |  | *p32* | MN955248 |
|  |  |  |  |  |  |  |  |  |  | *p24* | NS |
|  |  |  |  |  |  |  |  |  | BR_SP04 | *p29* | NS |
|  |  |  |  |  |  |  |  |  |  | *p32* | MN955249 |
|  |  |  |  |  |  |  |  |  |  | *p24* | NS |
|  |  |  |  |  |  |  |  |  | BR_SP05 | *p29* | NS |
|  |  |  |  |  |  |  |  |  |  | *p32* | MN955250 |
|  |  |  |  |  |  |  |  |  |  | *p24* | NS |
| 207 | 113 | *C. sinensis* | single | Fruit | São Paulo, BR | commercial | 2017 | CRD | BR_SP06 | *p29* | NS |
|  |  |  |  |  |  |  |  |  |  | *p32* | MN955251 |
|  |  |  |  |  |  |  |  |  |  | *p24* | NS |
|  |  |  |  |  |  |  |  |  | BR_SP07 | *p29* | NS |
|  |  |  |  |  |  |  |  |  |  | *p32* | MN955252 |
|  |  |  |  |  |  |  |  |  |  | *p24* | NS |
|  |  |  |  |  |  |  |  |  | BR_SP08 | *p29* | NS |
|  |  |  |  |  |  |  |  |  |  | *p32* | MN955253 |
|  |  |  |  |  |  |  |  |  |  | *p24* | NS |
|  |  |  |  |  |  |  |  |  | BR_SP09 | *p29* | NS |
|  |  |  |  |  |  |  |  |  |  | *p32* | MN955254 |
|  |  |  |  |  |  |  |  |  |  | *p24* | NS |
|  |  |  |  |  |  |  |  |  | BR_SP10 | *p29* | NS |
|  |  |  |  |  |  |  |  |  |  | *p32* | MN955255 |
|  |  |  |  |  |  |  |  |  |  | *p24* | NS |
| 208 | 114 | *C. sinensis* (Pera) | single | Fruit | Tambaú, SP, BR | commercial | 2017 | CRD | BR_SP_Tmb01 | *p29* | MN955048 |
|  |  |  |  |  |  |  |  |  |  | *p32* | MN955256 |
|  |  |  |  |  |  |  |  |  |  | *p24* | NS |
|  |  |  |  |  |  |  |  |  | BR_SP_Tmb02 | *p29* | MN955049 |
|  |  |  |  |  |  |  |  |  |  | *p32* | MN955257 |
|  |  |  |  |  |  |  |  |  |  | *p24* | NS |
|  |  |  |  |  |  |  |  |  | BR_SP_Tmb03 | *p29* | MN955050 |
|  |  |  |  |  |  |  |  |  |  | *p32* | MN955258 |
|  |  |  |  |  |  |  |  |  |  | *p24* | NS |
|  |  |  |  |  |  |  |  |  | BR_SP_Tmb04 | *p29* | NS |
|  |  |  |  |  |  |  |  |  |  | *p32* | MN955259 |
|  |  |  |  |  |  |  |  |  |  | *p24* | NS |
|  |  |  |  |  |  |  |  |  | BR_SP_Tmb05 | *p29* | NS |
|  |  |  |  |  |  |  |  |  |  | *p32* | MN955260 |
|  |  |  |  |  |  |  |  |  |  | *p24* | NS |
|  |  |  |  |  |  |  |  |  | BR_SP_Tmb06 | *p29* | NS |
|  |  |  |  |  |  |  |  |  |  | *p32* | MN955261 |
|  |  |  |  |  |  |  |  |  |  | *p24* | NS |
|  |  |  |  |  |  |  |  |  | BR_SP_Tmb07 | *p29* | NS |
|  |  |  |  |  |  |  |  |  |  | *p32* | MN955262 |
|  |  |  |  |  |  |  |  |  |  | *p24* | NS |
| 209-212 |  |  | single |  |  |  |  | CRD | BR_SP_Tmb14 to Tmb17 | *p29* | NS |
|  |  |  |  |  |  |  |  |  |  | *p32* | NS |
|  |  |  |  |  |  |  |  |  |  | *p24* | NS |
| 213 | 115 | *C. sinensis* (Valencia) | single | Fruit | Tambaú, SP, BR | commercial | 2017 | CRD+SJP | BR_SP_Tmb08 | *p29* | MN955051 |
|  |  |  |  |  |  |  |  |  |  | *p32* | MN955263 |
|  |  |  |  |  |  |  |  |  |  | *p24* | NS |
|  |  |  |  |  |  |  |  |  | BR_SP_Tmb09 | *p29* | MN955052 |
|  |  |  |  |  |  |  |  |  |  | *p32* | NS |
|  |  |  |  |  |  |  |  |  |  | *p24* | NS |
|  |  |  |  |  |  |  |  |  | BR_SP_Tmb10 | *p29* | MN955053 |
|  |  |  |  |  |  |  |  |  |  | *p32* | NS |
|  |  |  |  |  |  |  |  |  |  | *p24* | NS |
|  |  |  |  |  |  |  |  |  | BR_SP_Tmb11 | *p29* | MN955054 |
|  |  |  |  |  |  |  |  |  |  | *p32* | NS |
|  |  |  |  |  |  |  |  |  |  | *p24* | NS |
|  |  |  |  |  |  |  |  |  | BR_SP_Tmb12 | *p29* | MN955055 |
|  |  |  |  |  |  |  |  |  |  | *p32* | NS |
|  |  |  |  |  |  |  |  |  |  | *p24* | NS |
|  |  |  |  |  |  |  |  |  | BR_SP_Tmb13 | *p29* | MN955056 |
|  |  |  |  |  |  |  |  |  |  | *p32* | NS |
|  |  |  |  |  |  |  |  |  |  | *p24* | NS |
| 214-231 |  |  | single |  |  |  |  | CRD or CRD+SJP | BR_SP_Tmb18 to Tmb33 | *p29* | NS |
|  |  |  |  |  |  |  |  |  |  | *p32* | NS |
|  |  |  |  |  |  |  |  |  |  | *p24* | NS |
| 232 | 116 | *C. sinensis* (Hamlin) | single | Fruit | Taquaral, SP, BR | commercial | 2017 | SJP | BR_SP_Tql01 | *p29* | MN955058 |
|  |  |  |  |  |  |  |  |  |  | *p32* | MN955264 |
|  |  |  |  |  |  |  |  |  |  | *p24* | NS |
|  |  |  |  |  |  |  |  |  | BR_SP_Tql02 | *p29* | MN955059 |
|  |  |  |  |  |  |  |  |  |  | *p32* | MN955265 |
|  |  |  |  |  |  |  |  |  |  | *p24* | NS |
|  |  |  |  |  |  |  |  |  | BR_SP_Tql03 | *p29* | MN955060 |
|  |  |  |  |  |  |  |  |  |  | *p32* | MN955266 |
|  |  |  |  |  |  |  |  |  |  | *p24* | NS |
|  |  |  |  |  |  |  |  |  | BR_SP_Tql04 | *p29* | NS |
|  |  |  |  |  |  |  |  |  |  | *p32* | MN955267 |
|  |  |  |  |  |  |  |  |  |  | *p24* | NS |
|  |  |  |  |  |  |  |  |  | BR_SP_Tql05 | *p29* | NS |
|  |  |  |  |  |  |  |  |  |  | *p32* | MN955268 |
|  |  |  |  |  |  |  |  |  |  | *p24* | NS |
|  |  |  |  |  |  |  |  |  | BR_SP_Tql06 | *p29* | NS |
|  |  |  |  |  |  |  |  |  |  | *p32* | MN955269 |
|  |  |  |  |  |  |  |  |  |  | *p24* | NS |
|  |  |  |  |  |  |  |  |  | BR_SP_Tql07 | *p29* | NS |
|  |  |  |  |  |  |  |  |  |  | *p32* | MN955270 |
|  |  |  |  |  |  |  |  |  |  | *p24* | NS |
| 233-237 |  |  | single |  |  |  |  | CRD+SJP | BR_SP_Tql08-Tql12 | *p29* | NS |
|  |  |  |  |  |  |  |  |  |  | *p32* | NS |
|  |  |  |  |  |  |  |  |  |  | *p24* | NS |
| 238 | 117 | *C. sinensis* (Pera) | single | Fruit | Taquaritinga, SP, BR | commercial | 2017 | SJP | BR_SP_Tqt01 | *p29* | MN955061 |
|  |  |  |  |  |  |  |  |  |  | *p32* | MN955271 |
|  |  |  |  |  |  |  |  |  |  | *p24* | NS |
|  |  |  |  |  |  |  |  |  | BR_SP_Tqt02 | *p29* | MN955062 |
|  |  |  |  |  |  |  |  |  |  | *p32* | MN955272 |
|  |  |  |  |  |  |  |  |  |  | *p24* | NS |
|  |  |  |  |  |  |  |  |  | BR_SP_Tqt03 | *p29* | NS |
|  |  |  |  |  |  |  |  |  |  | *p32* | MN955273 |
|  |  |  |  |  |  |  |  |  |  | *p24* | NS |
|  |  |  |  |  |  |  |  |  | BR_SP_Tqt04 | *p29* | NS |
|  |  |  |  |  |  |  |  |  |  | *p32* | MN955274 |
|  |  |  |  |  |  |  |  |  |  | *p24* | NS |
| 239 | 118 | *C. sinensis* (Pera) | single | Fruit | Uberaba, MG, BR | commercial | 2017 | SJP | BR_MG_Uba02 | *p29* | MN955067 |
|  |  |  |  |  |  |  |  |  |  | *p32* | MN955275 |
|  |  |  |  |  |  |  |  |  |  | *p24* | NS |
|  |  |  |  |  |  |  |  |  | BR_MG_Uba03 | *p29* | MN955068 |
|  |  |  |  |  |  |  |  |  |  | *p32* | MN955276 |
|  |  |  |  |  |  |  |  |  |  | *p24* | NS |
|  |  |  |  |  |  |  |  |  | BR_MG_Uba04 | *p29* | MN955069 |
|  |  |  |  |  |  |  |  |  |  | *p32* | MN955277 |
|  |  |  |  |  |  |  |  |  |  | *p24* | NS |
|  |  |  |  |  |  |  |  |  | BR_MG_Uba05 | *p29* | NS |
|  |  |  |  |  |  |  |  |  |  | *p32* | MN955278 |
|  |  |  |  |  |  |  |  |  |  | *p24* | NS |
|  |  |  |  |  |  |  |  |  | BR_MG_Uba06 | *p29* | NS |
|  |  |  |  |  |  |  |  |  |  | *p32* | MN955279 |
|  |  |  |  |  |  |  |  |  |  | *p24* | NS |
|  |  |  |  |  |  |  |  |  | BR_MG_Uba07 | *p29* | NS |
|  |  |  |  |  |  |  |  |  |  | *p32* | MN955280 |
|  |  |  |  |  |  |  |  |  |  | *p24* |  |
| 240 |  |  | single |  |  |  |  | SJP | BR_MG_Uba08 | *p29* | MN955070 |
|  |  |  |  |  |  |  |  |  |  | *p32* | MN955281 |
|  |  |  |  |  |  |  |  |  |  | *p24* | NS |
|  |  |  |  |  |  |  |  |  | BR_MG_Uba09 | *p29* | MN955071 |
|  |  |  |  |  |  |  |  |  |  | *p32* | MN955282 |
|  |  |  |  |  |  |  |  |  |  | *p24* | NS |
|  |  |  |  |  |  |  |  |  | BR_MG_Uba10 | *p29* | NS |
|  |  |  |  |  |  |  |  |  |  | *p32* | MN955283 |
|  |  |  |  |  |  |  |  |  |  | *p24* | NS |
|  |  |  |  |  |  |  |  |  | BR_MG_Uba11 | *p29* | NS |
|  |  |  |  |  |  |  |  |  |  | *p32* | MN955284 |
|  |  |  |  |  |  |  |  |  |  | *p24* | NS |
|  |  |  |  |  |  |  |  |  | BR_MG_Uba12 | *p29* | NS |
|  |  |  |  |  |  |  |  |  |  | *p32* | MN955285 |
|  |  |  |  |  |  |  |  |  |  | *p24* | NS |
|  |  |  |  |  |  |  |  |  | BR_MG_Uba13 | *p29* | NS |
|  |  |  |  |  |  |  |  |  |  | *p32* | MN955286 |
|  |  |  |  |  |  |  |  |  |  | *p24* | NS |
|  |  |  |  |  |  |  |  |  | BR_MG_Uba14 | *p29* | NS |
|  |  |  |  |  |  |  |  |  |  | *p32* | MN955287 |
|  |  |  |  |  |  |  |  |  |  | *p24* | NS |
|  |  |  |  |  |  |  |  |  | BR_MG_Uba15 | *p29* | NS |
|  |  |  |  |  |  |  |  |  |  | *p32* | MN955288 |
|  |  |  |  |  |  |  |  |  |  | *p24* | NS |
|  |  |  |  |  |  |  |  |  | BR_MG_Uba16 | *p29* | NS |
|  |  |  |  |  |  |  |  |  |  | *p32* | MN955289 |
|  |  |  |  |  |  |  |  |  |  | *p24* | NS |
| 241 |  |  | single |  |  |  |  | SJP | BR_MG_Uba17 | *p29* | MN955072 |
|  |  |  |  |  |  |  |  |  |  | *p32* | NS |
|  |  |  |  |  |  |  |  |  |  | *p24* | NS |
|  |  |  |  |  |  |  |  |  | BR_MG_Uba18 | *p29* | MN955073 |
|  |  |  |  |  |  |  |  |  |  | *p32* | NS |
|  |  |  |  |  |  |  |  |  |  | *p24* | NS |
|  |  |  |  |  |  |  |  |  | BR_MG_Uba19 | *p29* | MN955074 |
|  |  |  |  |  |  |  |  |  |  | *p32* | NS |
|  |  |  |  |  |  |  |  |  |  | *p24* | NS |
|  |  |  |  |  |  |  |  |  | BR_MG_Uba20 | *p29* | MN955075 |
|  |  |  |  |  |  |  |  |  |  | *p32* | NS |
|  |  |  |  |  |  |  |  |  |  | *p24* | NS |
| 242-245 |  |  | single |  |  |  |  | SJP | BR_MG_Uba21 to Uba24 | *p29* | NS |
|  |  |  |  |  |  |  |  |  |  | *p32* | NS |
|  |  |  |  |  |  |  |  |  |  | *p24* | NS |
| 246 | 119 | *C. sinensis* | single | Fruit | Aguaí, SP, BR | commercial | 2018 | SJP | BR_SP_Agi_27 | *p29* | NS |
|  |  |  |  |  |  |  |  |  |  | *p24* | NS |
| 247 | 120 | *C. sinensis* (Natal) | single | Fruit | Aguaí, SP, BR | commercial | 2018 | CRD+SJP | BR_SP_Agi_28 | *p29* | NS |
|  |  |  |  |  |  |  |  |  |  | *p24* | NS |
| 248 | 121 | *C. sinensis* | single | Fruit | Aguaí, SP, BR | commercial | 2018 | SJP | BR_SP_Agi_29 | *p29* | NS |
|  |  |  |  |  |  |  |  |  |  | *p24* | NS |
| 249 | 122 | *C. sinensis* (Pera) | single | Fruit | Aguaí, SP, BR | commercial | 2018 | SJP | BR_SP_Agi_30 | *p29* | NS |
|  |  |  |  |  |  |  |  |  |  | *p24* | NS |
| 250 | 123 | *C. sinensis* (Pera) | single | Fruit | Aguaí, SP, BR | commercial | 2018 | CRD+SJP | BR_SP_Agi_31 | *p29* | NS |
|  |  |  |  |  |  |  |  |  |  | *p24* | NS |
|  |  |  |  |  |  |  |  |  | BR_SP_Agi_32 | *p29* | NS |
|  |  |  |  |  |  |  |  |  |  | *p24* | NS |
| 251 | 124 | *C. sinensis* (Pera) | single | Fruit | Aguaí, SP, BR | commercial | 2018 | CRD | BR_SP_Agi_33 | *p29* | NS |
|  |  |  |  |  |  |  |  |  |  | *p24* | NS |
| 252 | 125 | *C. sinensis* (Pera) | single | Fruit | Águas de Santa Barbara, SP, BR | commercial | 2018 | SJP | BR_SP_ASB01 | *p29* | NS |
|  |  |  |  |  |  |  |  |  |  | *p24* | NS |
| 253 | 126 | *C. sinensis* | single | Fruit | Altinópolis, SP, BR | commercial | 2018 | SJP | BR_SP_Alt23 | *p29* | NS |
|  |  |  |  |  |  |  |  |  |  | *p24* | NS |
| 254 | 127 | *C. sinensis* (Pera) | single | Fruit | Angatuba, SP, BR | commercial | 2018 | CRD+SJP | BR_SP_Ang01 | *p29* | NS |
|  |  |  |  |  |  |  |  |  |  | *p24* | NS |
| 255 | 128 | *C. sinensis* (Pera) | single | Fruit | Anhembi, SP, BR | commercial | 2018 | CRD+SJP | BR_SP_Amb01 | *p29* | NS |
|  |  |  |  |  |  |  |  |  |  | *p24* | NS |
| 256 | 129 | *C. sinensis* (Pera) | single | Fruit | Arandu, SP, BR | commercial | 2018 | CRD+SJP | BR_SP_Ard01 | *p29* | NS |
|  |  |  |  |  |  |  |  |  |  | *p24* | NS |
| 257 | 130 | *C. sinensis* (Pera) | single | Fruit | Artur Nogueira, SP, BR | commercial | 2018 | CRD | BR_SP_ArN01 | *p29* | NS |
|  |  |  |  |  |  |  |  |  |  | *p24* | NS |
| 258 | 131 | *C. sinensis* (Pera) | single | Fruit | Avaí, SP, BR | commercial | 2018 | SJP | BR_SP_Ava01 | *p29* | NS |
|  |  |  |  |  |  |  |  |  |  | *p24* | NS |
| 259 | 132 | *C. sinensis* (Pera) | single | Fruit | Avaré, SP, BR | commercial | 2018 | CRD+SJP | BR_SP_Avr02 | *p29* | NS |
|  |  |  |  |  |  |  |  |  |  | *p24* | NS |
|  |  |  |  |  |  |  |  |  | BR_SP_Avr03 | *p29* | NS |
|  |  |  |  |  |  |  |  |  |  | *p24* | NS |
| 260 | 133 | *C. sinensis* (Pera) | single | Fruit | Avaré, SP, BR | commercial | 2018 | SJP | BR_SP_Avr04 | *p29* | NS |
|  |  |  |  |  |  |  |  |  |  | *p24* | NS |
| 261 | 134 | *C. sinensis* (Pera) | single | Fruit | Avaré, SP, BR | commercial | 2018 | CRD | BR_SP_Avr05 | *p29* | NS |
|  |  |  |  |  |  |  |  |  |  | *p24* | NS |
| 262 | 135 | *C. sinensis* (Pera) | single | Fruit | Barretos, SP, BR | commercial | 2018 | CRD+SJP | BR_SP_Bar25 | *p29* | NS |
|  |  |  |  |  |  |  |  |  |  | *p24* | NS |
| 263 | 136 | *C. sinensis* (Pera) | single | Fruit | Bauru, SP, BR | commercial | 2018 | SJP | BR_SP_Bur01 | *p29* | NS |
|  |  |  |  |  |  |  |  |  |  | *p24* | NS |
| 264 | 137 | *C. sinensis* (Pera) | single | Fruit | Bebedouro, SP, BR | commercial | 2018 | SJP | BR_SP_Beb33 | *p29* | NS |
|  |  |  |  |  |  |  |  |  |  | *p24* | NS |
| 265 | 138 | *C. sinensis* | single | Fruit | Boa Esperança do Sul, SP, BR | commercial | 2018 | SJP | BR_SP_BES01 | *p29* | NS |
|  |  |  |  |  |  |  |  |  |  | *p24* | NS |
| 264 | 139 | *C. sinensis* | single | Fruit | Boa Esperança do Sul, SP, BR | commercial | 2018 | SJP | BR_SP_BES02 | *p29* | NS |
|  |  |  |  |  |  |  |  |  |  | *p24* | NS |
| 267 | 140 | *C. sinensis* (Pera) | single | Fruit | Brotas, SP, BR | commercial | 2018 | CRD+SJP | BR_SP_Bro15 | *p29* | NS |
|  |  |  |  |  |  |  |  |  |  | *p24* | NS |
| 268 | 141 | *C. sinensis* | single | Fruit | Brotas, SP, BR | commercial | 2018 | SJP | BR_SP_Bro16 | *p29* | NS |
|  |  |  |  |  |  |  |  |  |  | *p24* | NS |
| 269 | 142 | *C. sinensis* (Pera) | single | Fruit | Brotas, SP, BR | commercial | 2018 | SJP | BR_SP_Bro17 | *p29* | NS |
|  |  |  |  |  |  |  |  |  |  | *p24* | NS |
| 270 | 143 | *C. sinensis* | single | Fruit | Cabrália Paulista, SP, BR | commercial | 2018 | CRD | BR_SP_CbP01 | *p29* | NS |
|  |  |  |  |  |  |  |  |  |  | *p24* | NS |
| 271 | 144 | *C. sinensis* | single | Fruit | Cajobi, SP, BR | commercial | 2018 | SJP | BR_SP_Cjb01 | *p29* | NS |
|  |  |  |  |  |  |  |  |  |  | *p24* | NS |
| 272 | 145 | *C. sinensis* | single | Fruit | Casa Branca, SP, BR | commercial | 2018 | CRD+SJP | BR_SP_CsB02 | *p29* | NS |
|  |  |  |  |  |  |  |  |  |  | *p24* | NS |
|  |  |  |  |  |  |  |  |  | BR_SP_CsB03 | *p29* | NS |
|  |  |  |  |  |  |  |  |  |  | *p24* | NS |
| 273 | 146 | *C. sinensis* | single | Fruit | Casa Branca, SP, BR | commercial | 2018 | CRD+SJP | BR_SP_CsB04 | *p29* | NS |
|  |  |  |  |  |  |  |  |  |  | *p24* | NS |
|  |  |  |  |  |  |  |  |  | BR_SP_CsB05 | *p29* | NS |
|  |  |  |  |  |  |  |  |  |  | *p24* | NS |
| 274 | 147 | *C. sinensis* (Pera) | single | Fruit | Casa Branca, SP, BR | commercial | 2018 | CRD+SJP | BR_SP_CsB06 | *p29* | NS |
|  |  |  |  |  |  |  |  |  | BR_SP_CsB07 | *p24* | NS |
| 275 | 148 | *C. sinensis* (Pera) | single | Fruit | Casa Branca, SP, BR | commercial | 2018 | CRD | BR_SP_CsB08 | *p29* | NS |
|  |  |  |  |  |  |  |  |  |  | *p24* | NS |
| 276 | 149 | *C. sinensis* | single | Fruit | Casa Branca, SP, BR | commercial | 2018 | CRD | BR_SP_CsB09 | *p29* | NS |
|  |  |  |  |  |  |  |  |  |  | *p24* | NS |
| 277 | 150 | *C. sinensis* | single | Fruit | Casa Branca, SP, BR | commercial | 2018 | CRD+SJP | BR_SP_CsB10 | *p29* | NS |
|  |  |  |  |  |  |  |  |  |  | *p24* | NS |
|  |  |  |  |  |  |  |  |  | BR_SP_CsB11 | *p29* | NS |
|  |  |  |  |  |  |  |  |  |  | *p24* | NS |
| 278 | 151 | *C. sinensis* (Pera) | single | Fruit | Cedral, SP, BR | commercial | 2018 | SJP | BR_SP_Cdl01 | *p29* | NS |
|  |  |  |  |  |  |  |  |  |  | *p24* | NS |
| 279 | 152 | *C. sinensis* (Pera) | single | Fruit | Cerqueira Cesar, SP, BR | commercial | 2018 | SJP | BR_SP_CrC29 | *p29* | NS |
|  |  |  |  |  |  |  |  |  |  | *p24* | NS |
| 280 | 153 | *C. sinensis* | single | Fruit | Colina, SP, BR | commercial | 2018 | CRD | BR_SP_Cln03 | *p29* | NS |
|  |  |  |  |  |  |  |  |  |  | *p24* | NS |
| 281 | 154 | *C. sinensis* (Pera) | single | Fruit | Colina, SP, BR | commercial | 2018 | SJP | BR_SP_Cln04 | *p29* | NS |
|  |  |  |  |  |  |  |  |  |  | *p24* | NS |
| 282 | 155 | *C. sinensis* | single | Fruit | Colômbia, SP, BR | commercial | 2018 | CRD+SJP | BR_SP_Clb17 | *p29* | NS |
|  |  |  |  |  |  |  |  |  | BR_SP_Clb18 | *p24* | NS |
| 283 | 156 | *C. sinensis* | single | Fruit | Comendador Gomes, MG, BR | commercial | 2018 | SJP | BR_MG_Cgz05 | *p29* | NS |
|  |  |  |  |  |  |  |  |  |  | *p24* | NS |
| 284 | 157 | *C. sinensis* | single | Fruit | Conchal, SP, BR | commercial | 2018 | CRD+SJP | BR_SP_Cch03 | *p29* | NS |
|  |  |  |  |  |  |  |  |  | BR_SP_Cch04 | *p24* | NS |
| 285 | 158 | *C. sinensis* | single | Fruit | Conchal, SP, BR | commercial | 2018 | CRD+SJP | BR_SP_Cch05 | *p29* | NS |
|  |  |  |  |  |  |  |  |  | BR_SP_Cch06 | *p24* | NS |
| 286 | 159 | *C. sinensis* | single | Fruit | Conchal, SP, BR | commercial | 2018 | CRD | BR_SP_Cch07 | *p29* | NS |
|  |  |  |  |  |  |  |  |  |  | *p24* | NS |
| 287 | 160 | *C. sinensis* (Natal) | single | Fruit | Conchal, SP, BR | commercial | 2018 | SJP | BR_SP_Cch08 | *p29* | NS |
|  |  |  |  |  |  |  |  |  |  | *p24* | NS |
| 288 | 161 | *C. sinensis* (Natal) | single | Fruit | Conchal, SP, BR | commercial | 2018 | SJP | BR_SP_Cch09 | *p29* | NS |
|  |  |  |  |  |  |  |  |  |  | *p24* | NS |
| 289 | 162 | *C. sinensis* | single | Fruit | Conchal, SP, BR | commercial | 2018 | CRD+SJP | BR_SP_Cch10 | *p29* | NS |
|  |  |  |  |  |  |  |  |  | BR_SP_Cch11 | *p24* | NS |
| 290 | 163 | *C. sinensis* | single | Fruit | Engenheiro Coelho, SP, BR | commercial | 2018 | SJP | BR_SP_EgC01 | *p29* | NS |
|  |  |  |  |  |  |  |  |  |  | *p24* | NS |
| 291 | 164 | *C. sinensis* | single | Fruit | Engenheiro Coelho, SP, BR | commercial | 2018 | CRD+SJP | BR_SP_EgC02 | *p29* | NS |
|  |  |  |  |  |  |  |  |  |  | *p24* | NS |
| 292 | 165 | *C. sinensis* (Natal) | single | Fruit | Estiva Gerbi, SP, BR | commercial | 2018 | CRD | BR_SP_EtG01 | *p29* | NS |
|  |  |  |  |  |  |  |  |  |  | *p24* | NS |
| 293 | 166 | *C. sinensis* | single | Fruit | Fernando Prestes, SP, BR | commercial | 2018 | SJP | BR_SP_FrP01 | *p29* | NS |
|  |  |  |  |  |  |  |  |  |  | *p24* | NS |
| 294 | 167 | *C. sinensis* (Pera) | single | Fruit | Fernão, SP, BR | commercial | 2018 | SJP | BR_SP_Frn01 | *p29* | NS |
|  |  |  |  |  |  |  |  |  |  | *p24* | NS |
| 295 | 168 | *C. sinensis* (Pera) | single | Fruit | Frutal, MG, BR | commercial | 2018 | SJP | BR_MG_Frt02 | *p29* | NS |
|  |  |  |  |  |  |  |  |  |  | *p24* | NS |
| 296 | 169 | *C. sinensis* | single | Fruit | Frutal, MG, BR | commercial | 2018 | SJP | BR_MG_Frt03 | *p29* | NS |
|  |  |  |  |  |  |  |  |  |  | *p24* | NS |
| 297 | 170 | *C. sinensis* | single | Fruit | Gavião Peixoto, SP, BR | commercial | 2018 | SJP | BR_SP_GvP01 | *p29* | NS |
|  |  |  |  |  |  |  |  |  |  | *p24* | NS |
| 298 | 171 | *C. sinensis* | single | Fruit | Gavião Peixoto, SP, BR | commercial | 2018 | SJP | BR_SP_GvP02 | *p29* | NS |
|  |  |  |  |  |  |  |  |  |  | *p24* | NS |
| 299 | 172 | *C. sinensis* (Pera) | single | Fruit | Getulina, SP, BR | commercial | 2018 | SJP | BR_SP_Gtl01 | *p29* | NS |
|  |  |  |  |  |  |  |  |  |  | *p24* | NS |
| 300 | 173 | *C. sinensis* | single | Fruit | Guarantã, SP, BR | commercial | 2018 | SJP | BR_SP_Grt02 | *p29* | NS |
|  |  |  |  |  |  |  |  |  |  | *p24* | NS |
| 301 | 174 | *C. sinensis* | single | Fruit | Guarantã, SP, BR | commercial | 2018 | CRD+SJP | BR_SP_Grt03 | *p29* | NS |
|  |  |  |  |  |  |  |  |  | BR_SP_Grt04 | *p24* | NS |
| 302 | 175 | *C. sinensis* (Pera) | single | Fruit | Iacanga, SP, BR | commercial | 2018 | SJP | BR_SP_Icg01 | *p29* | NS |
|  |  |  |  |  |  |  |  |  |  | *p24* | NS |
| 303 | 176 | *C. sinensis* | single | Fruit | Ibirá, SP, BR | commercial | 2018 | SJP | BR_SP_Ibr01 | *p29* | NS |
|  |  |  |  |  |  |  |  |  |  | *p24* | NS |
| 304 | 177 | *C. sinensis* (Natal) | single | Fruit | Ibiraci, MG, BR | commercial | 2018 | CRD+SJP | BR_SP_Ibc01 | *p29* | NS |
|  |  |  |  |  |  |  |  |  | BR_SP_Ibc02 | *p24* | NS |
| 305 | 178 | *C. sinensis* (Pera) | single | Fruit | Ibitinga, SP, BR | commercial | 2018 | SJP | BR_SP_Ibt01 | *p29* | NS |
|  |  |  |  |  |  |  |  |  |  | *p24* | NS |
| 306 | 179 | *C. sinensis* (Natal) | single | Fruit | Ibitinga, SP, BR | commercial | 2018 | SJP | BR_SP_Ibt02 | *p29* | NS |
|  |  |  |  |  |  |  |  |  |  | *p24* | NS |
| 307 | 180 | *C. sinensis* | single | Fruit | Ipiguá, SP, BR | commercial | 2018 | SJP | BR_SP_Ipg01 | *p29* | NS |
|  |  |  |  |  |  |  |  |  |  | *p24* | NS |
| 308 | 181 | *C. sinensis* | single | Fruit | Itápolis, SP, BR | commercial | 2018 | SJP | BR_SP_Itp01 | *p29* | NS |
|  |  |  |  |  |  |  |  |  |  | *p24* | NS |
| 309 | 182 | *C. sinensis* (Natal) | single | Fruit | Itápolis, SP, BR | commercial | 2018 | SJP | BR_SP_Itp02 | *p29* | NS |
|  |  |  |  |  |  |  |  |  |  | *p24* | NS |
| 310 | 183 | *C. sinensis* (Pera) | single | Fruit | Itápolis, SP, BR | commercial | 2018 | SJP | BR_SP_Itp03 | *p29* | NS |
|  |  |  |  |  |  |  |  |  |  | *p24* | NS |
| 311 | 184 | *C. sinensis* (Pera) | single | Fruit | Itápolis, SP, BR | commercial | 2018 | SJP | BR_SP_Itp04 | *p29* | NS |
|  |  |  |  |  |  |  |  |  |  | *p24* | NS |
| 312 | 185 | *C. sinensis* | single | Fruit | Itápolis, SP, BR | commercial | 2018 | CRD | BR_SP_Itp05 | *p29* | NS |
|  |  |  |  |  |  |  |  |  |  | *p24* | NS |
| 313 | 186 | *C. sinensis* (Natal) | single | Fruit | Itápolis, SP, BR | commercial | 2018 | SJP | BR_SP_Itp06 | *p29* | NS |
|  |  |  |  |  |  |  |  |  |  | *p24* | NS |
| 314 | 187 | *C. sinensis* | single | Fruit | Itápolis, SP, BR | commercial | 2018 | CRD+SJP | BR_SP_Itp07 | *p29* | NS |
|  |  |  |  |  |  |  |  |  | BR_SP_Itp08 | *p24* | NS |
| 315 | 188 | *C. sinensis* | single | Fruit | Itápolis, SP, BR | commercial | 2018 | SJP | BR_SP_Itp08 | *p29* | NS |
|  |  |  |  |  |  |  |  |  |  | *p24* | NS |
| 316 | 189 | *C. sinensis* | single | Fruit | Itápolis, SP, BR | commercial | 2018 | CRD+SJP | BR_SP_Itp09 | *p29* | NS |
|  |  |  |  |  |  |  |  |  |  | *p24* | NS |
|  |  |  |  |  |  |  |  |  | BR_SP_Itp10 | *p29* | NS |
|  |  |  |  |  |  |  |  |  |  | *p24* | NS |
| 317 | 190 | *C. sinensis* (Pera) | single | Fruit | Ituiutaba, MG, BR | commercial | 2018 | SJP | BR_MG_Itb01 | *p29* | MN955017 |
|  |  |  |  |  |  |  |  |  |  | *p15* | MT304683 |
|  |  |  |  |  |  |  |  |  |  | *p32* | MN955195 |
|  |  |  |  |  |  |  |  |  |  | *p24* | NS |
|  |  |  |  |  |  |  |  |  | BR_MG_Itb02 | *p29* | NS |
|  |  |  |  |  |  |  |  |  |  | *p15* | NS |
|  |  |  |  |  |  |  |  |  |  | *p32* | MN955196 |
|  |  |  |  |  |  |  |  |  |  | *p24* | NS |
| 318 | 191 | *C. sinensis* (Pera) | single | Fruit | Júlio Mesquita, SP, BR | commercial | 2018 | SJP | BR_SP_JlM01 | *p29* | NS |
|  |  |  |  |  |  |  |  |  |  | *p24* | NS |
| 319 | 192 | *C. reticulata* | single | Fruit | Júlio Mesquita, SP, BR | commercial | 2018 | CRD+SJP | BR_SP_JlM02 | *p29* | NS |
|  |  |  |  |  |  |  |  |  |  | *p24* | NS |
|  |  |  |  |  |  |  |  |  | BR_SP_JlM03 | *p29* | NS |
|  |  |  |  |  |  |  |  |  |  | *p24* | NS |
| 320 | 193 | *C. sinensis* | single | Leaf | Jumirin, SP, BR | non-commercial | 2018 | CRD | BR_SP_Jmr01 | *p29* | NS |
|  |  |  |  |  |  |  |  |  |  | *p24* | NS |
| 321 | 194 | *C. sinensis* (Pera) | single | Fruit | Limeira, SP, BR | commercial | 2018 | SJP | BR_SP_Lim03 | *p29* | NS |
|  |  |  |  |  |  |  |  |  |  | *p24* | NS |
| 322 | 195 | *C. sinensis* | single | Fruit | Limeira, SP, BR | commercial | 2018 | SJP | BR_SP_Lim04 | *p29* | NS |
|  |  |  |  |  |  |  |  |  |  | *p24* | NS |
| 323 | 196 | *C. sinensis* | single | Fruit | Limeira, SP, BR | commercial | 2018 | CRD+SJP | BR_SP_Lim05 | *p29* | NS |
|  |  |  |  |  |  |  |  |  |  | *p24* | NS |
|  |  |  |  |  |  |  |  |  | BR_SP_Lim06 | *p29* | NS |
|  |  |  |  |  |  |  |  |  |  | *p24* | NS |
| 324 | 197 | *C. sinensis* | single | Fruit | Limeira, SP, BR | commercial | 2018 | SJP | BR_SP_Lim07 | *p29* | NS |
|  |  |  |  |  |  |  |  |  |  | *p24* | NS |
| 325 | 198 | *C. sinensis* | single | Fruit | Limeira, SP, BR | commercial | 2018 | CRD | BR_SP_Lim08 | *p29* | NS |
|  |  |  |  |  |  |  |  |  |  | *p24* | NS |
| 326 | 199 | *C. sinensis* | single | Fruit | Lucianópolis, SP, BR | commercial | 2018 | CRD | BR_SP_Lcp01 | *p29* | NS |
|  |  |  |  |  |  |  |  |  |  | *p24* | NS |
| 327 | 200 | *C. sinensis* | single | Fruit | Lucianópolis, SP, BR | commercial | 2018 | SJP | BR_SP_Lcp02 | *p29* | NS |
|  |  |  |  |  |  |  |  |  |  | *p24* | NS |
| 328 | 201 | *C. sinensis* (Natal) | single | Fruit | Lucianópolis, SP, BR | commercial | 2018 | SJP | BR_SP_Lcp03 | *p29* | NS |
|  |  |  |  |  |  |  |  |  |  | *p24* | NS |
| 329 | 202 | *C. sinensis* (Pera) | single | Fruit | Mococa, SP, BR | commercial | 2018 | SJP | BR_SP_Mcc01 | *p29* | NS |
|  |  |  |  |  |  |  |  |  |  | *p24* | NS |
| 330 | 203 | *C. sinensis* | single | Fruit | Mococa, SP, BR | commercial | 2018 | CRD+SJP | BR_SP_Mcc02 | *p29* | NS |
|  |  |  |  |  |  |  |  |  |  | *p24* | NS |
|  |  |  |  |  |  |  |  |  | BR_SP_Mcc03 | *p29* | NS |
|  |  |  |  |  |  |  |  |  |  | *p24* | NS |
| 331 | 204 | *C. sinensis* | single | Fruit | Mococa, SP, BR | commercial | 2018 | CRD | BR_SP_Mcc04 | *p29* | NS |
|  |  |  |  |  |  |  |  |  |  | *p24* | NS |
| 332 | 205 | *C. sinensis* | single | Fruit | Mococa, SP, BR | commercial | 2018 | SJP | BR_SP_Mcc05 | *p29* | NS |
|  |  |  |  |  |  |  |  |  |  | *p24* | NS |
| 333 | 206 | *C. sinensis* (Natal) | single | Fruit | Mogi Guaçu, SP, BR | commercial | 2018 | SJP | BR_SP_MgG01 | *p29* | NS |
|  |  |  |  |  |  |  |  |  |  | *p24* | NS |
| 334 | 207 | *C. sinensis* (Natal) | single | Fruit | Mogi Guaçu, SP, BR | commercial | 2018 | CRD+SJP | BR_SP_MgG02 | *p29* | NS |
|  |  |  |  |  |  |  |  |  |  | *p24* | NS |
|  |  |  |  |  |  |  |  |  | BR_SP_MgG03 | *p29* | NS |
|  |  |  |  |  |  |  |  |  |  | *p24* | NS |
| 335 | 208 | *C. sinensis* (Pera) | single | Fruit | Mogi Guaçu, SP, BR | commercial | 2018 | SJP | BR_SP_MgG04 | *p29* | NS |
|  |  |  |  |  |  |  |  |  |  | *p24* | NS |
| 336 | 209 | *C. sinensis* | single | Fruit | Mogi Guaçu, SP, BR | commercial | 2018 | CRD | BR_SP_MgG05 | *p29* | NS |
|  |  |  |  |  |  |  |  |  |  | *p24* | NS |
| 337 | 210 | *C. sinensis* | single | Fruit | Mogi Mirim, SP, BR | commercial | 2018 | CRD+SJP | BR_SP_MgM41 | *p29* | NS |
|  |  |  |  |  |  |  |  |  |  | *p24* | NS |
|  |  |  |  |  |  |  |  |  | BR_SP_MgM42 | *p29* | NS |
|  |  |  |  |  |  |  |  |  |  | *p24* | NS |
| 338 | 211 | *C. sinensis* | single | Fruit | Monte Azul Paulista, SP, BR | commercial | 2018 | SJP | BR_SP_MAP01 | *p29* | NS |
|  |  |  |  |  |  |  |  |  |  | *p24* | NS |
| 339 | 212 | *C. sinensis* (Pera) | single | Fruit | Monte Azul Paulista, SP, BR | commercial | 2018 | SJP | BR_SP_MAP02 | *p29* | NS |
|  |  |  |  |  |  |  |  |  |  | *p24* | NS |
| 340 | 213 | *C. sinensis* | single | Fruit | Monte Azul Paulista, SP, BR | commercial | 2018 | SJP | BR_SP_MAP03 | *p29* | NS |
|  |  |  |  |  |  |  |  |  |  | *p24* | NS |
| 341 | 214 | *C. sinensis* (Pera) | single | Fruit | Monte Azul Paulista, SP, BR | commercial | 2018 | SJP | BR_SP_MAP04 | *p29* | NS |
|  |  |  |  |  |  |  |  |  |  | *p24* | NS |
| 342 | 215 | *C. sinensis* | single | Fruit | Nova Granada, SP, BR | commercial | 2018 | CRD+SJP | BR_SP_NvG01 | *p29* | NS |
|  |  |  |  |  |  |  |  |  |  | *p24* | NS |
|  |  |  |  |  |  |  |  |  | BR_SP_NvG02 | *p29* | NS |
|  |  |  |  |  |  |  |  |  |  | *p24* | NS |
| 343 | 216 | *C. sinensis* (Natal) | single | Fruit | Olímpia, SP, BR | commercial | 2018 | SJP | BR_SP_Olm02 | *p29* | NS |
|  |  |  |  |  |  |  |  |  |  | *p24* | NS |
| 344 | 217 | *C. sinensis* (Pera) | single | Fruit | Olímpia, SP, BR | commercial | 2018 | SJP | BR_SP_Olm03 | *p29* | NS |
|  |  |  |  |  |  |  |  |  |  | *p24* | NS |
| 345 | 218 | *C. sinensis* (Pera) | single | Fruit | Olímpia, SP, BR | commercial | 2018 | SJP | BR_SP_Olm04 | *p29* | NS |
|  |  |  |  |  |  |  |  |  |  | *p24* | NS |
| 346 | 219 | *C. sinensis* (Natal) | single | Fruit | Paulo de Faria, SP, BR | commercial | 2018 | SJP | BR_SP_PlF01 | *p29* | NS |
|  |  |  |  |  |  |  |  |  |  | *p24* | NS |
| 347 | 220 | *C. sinensis* | single | Fruit | Pedranópolis, SP, BR | commercial | 2018 | SJP | BR_SP_Pdp01 | *p29* | NS |
|  |  |  |  |  |  |  |  |  |  | *p24* | NS |
| 348 | 221 | *C. sinensis* (Natal) | single | Fruit | Pedregulho, SP, BR | commercial | 2018 | SJP | BR_SP_Pdg01 | *p29* | NS |
|  |  |  |  |  |  |  |  |  |  | *p24* | NS |
| 349 | 222 | *C. sinensis* (Natal) | single | Fruit | Pedregulho, SP, BR | commercial | 2018 | SJP | BR_SP_Pdg02 | *p29* | NS |
|  |  |  |  |  |  |  |  |  |  | *p24* | NS |
| 350 | 223 | *C. sinensis* | single | Fruit | Pedregulho, SP, BR | commercial | 2018 | SJP | BR_SP_Pdg03 | *p29* | NS |
|  |  |  |  |  |  |  |  |  |  | *p24* | NS |
| 351 | 224 | *C. sinensis* (Natal) | single | Fruit | Pedrinópolis, MG, BR | commercial | 2018 | SJP | BR_MG_Pdp01 | *p29* | NS |
|  |  |  |  |  |  |  |  |  |  | *p24* | NS |
| 352 | 225 | *C. reticulata* | pool | Leaf | Piracicaba, SP, BR | non-commercial | 2018 | CRD | BR_SP_Prb03 | RNA1 | MT554534 |
|  |  |  |  |  |  |  |  |  |  | RNA2 | MT554548 |
| 353 | 226 | *C. sinensis* | single | Fruit | Piracicaba, SP, BR | commercial | 2018 | SJP | BR_SP_Prb08 | *p29* | NS |
|  |  |  |  |  |  |  |  |  |  | *p24* | NS |
| 354 | 227 | *C. sinensis* | single | Fruit | Piracicaba, SP, BR | commercial | 2018 | CRD+SJP | BR_SP_Prb09 | *p29* | NS |
|  |  |  |  |  |  |  |  |  |  | *p24* | NS |
|  |  |  |  |  |  |  |  |  | BR_SP_Prb10 | *p29* | NS |
|  |  |  |  |  |  |  |  |  |  | *p24* | NS |
| 355 | 228 | *C. sinensis* | single | Fruit | Piracicaba, SP, BR | commercial | 2018 | SJP | BR_SP_Prb11 | *p29* | NS |
|  |  |  |  |  |  |  |  |  |  | *p24* | NS |
| 356 | 229 | *C. sinensis* | single | Fruit | Pirajuí, SP, BR | commercial | 2018 | SJP | BR_SP_ Prj03 | *p29* | NS |
|  |  |  |  |  |  |  |  |  |  | *p24* | NS |
| 357 | 230 | *C. sinensis* | single | Fruit | Pirangi, SP, BR | commercial | 2018 | SJP | BR_SP_Png01 | *p29* | NS |
|  |  |  |  |  |  |  |  |  |  | *p24* | NS |
| 358 | 231 | *C. sinensis* | single | Fruit | Pirassununga, SP, BR | commercial | 2018 | CRD | BR_SP_Prg07 | *p29* | NS |
|  |  |  |  |  |  |  |  |  |  | *p24* | NS |
| 359 | 232 | *C. sinensis* (Pera) | single | Fruit | Pirassununga, SP, BR | commercial | 2018 | CRD+SJP | BR_SP_Prg08 | *p29* | NS |
|  |  |  |  |  |  |  |  |  |  | *p24* | NS |
|  |  |  |  |  |  |  |  |  | BR_SP_Prg09 | *p29* | NS |
|  |  |  |  |  |  |  |  |  |  | *p24* | NS |
| 360 | 233 | *C. sinensis* (Pera) | single | Fruit | Piratininga, SP, BR | commercial | 2018 | SJP | BR_SP_Ptn01 | *p29* | NS |
|  |  |  |  |  |  |  |  |  |  | *p24* | NS |
| 361 | 234 | *C. sinensis* (Pera) | single | Fruit | Piratininga, SP, BR | commercial | 2018 | CRD | BR_SP_Ptn02 | *p29* | NS |
|  |  |  |  |  |  |  |  |  |  | *p24* | NS |
| 362 | 235 | *C. sinensis* | single | Fruit | Piratininga, SP, BR | commercial | 2018 | SJP | BR_SP_Ptn03 | *p29* | NS |
|  |  |  |  |  |  |  |  |  |  | *p24* | NS |
| 363 | 236 | *C. sinensis* | single | Fruit | Potirendaba, SP, BR | commercial | 2018 | SJP | BR_SP_Pob01 | *p29* | NS |
|  |  |  |  |  |  |  |  |  |  | *p24* | NS |
| 364 | 237 | *C. sinensis* (Pera) | single | Fruit | Potirendaba, SP, BR | commercial | 2018 | SJP | BR_SP_Pob02 | *p29* | NS |
|  |  |  |  |  |  |  |  |  |  | *p24* | NS |
| 365 | 238 | *C. sinensis* (Pera) | single | Fruit | Prata, MG, BR | commercial | 2018 | SJP | BR_MG_Prt01 | *p29* | NS |
|  |  |  |  |  |  |  |  |  |  | *p24* | NS |
| 366 | 239 | *C. sinensis* (Pera) | single | Fruit | Reginópolis, SP, BR | commercial | 2018 | CRD+SJP | BR_SP_Rgp01 | *p29* | NS |
|  |  |  |  |  |  |  |  |  |  | *p24* | NS |
|  |  |  |  |  |  |  |  |  | BR_SP_Rgp02 | *p29* | NS |
|  |  |  |  |  |  |  |  |  |  | *p24* | NS |
| 367 | 240 | *C. sinensis* | single | Fruit | Ribeirão Bonito, SP, BR | commercial | 2018 | SJP | BR_SP_RbB01 | *p29* | NS |
|  |  |  |  |  |  |  |  |  |  | *p24* | NS |
| 368 | 241 | *C. sinensis* | single | Fruit | Ribeirão Bonito, SP, BR | commercial | 2018 | SJP | BR_SP_RbB02 | *p29* | NS |
|  |  |  |  |  |  |  |  |  |  | *p24* | NS |
| 369 | 242 | *C. sinensis* | single | Fruit | Rincão, SP, BR | commercial | 2018 | SJP | BR_SP_Rnc01 | *p29* | NS |
|  |  |  |  |  |  |  |  |  |  | *p24* | NS |
| 370 | 243 | *C. sinensis* | single | Fruit | Rio Claro, SP, BR | commercial | 2018 | SJP | BR_SP_RiC01 | *p29* | NS |
|  |  |  |  |  |  |  |  |  |  | *p24* | NS |
| 371 | 244 | *C. sinensis* | single | Fruit | Riolândia, SP, BR | commercial | 2018 | CRD | BR_SP_Rld01 | *p29* | NS |
|  |  |  |  |  |  |  |  |  |  | *p24* | NS |
| 372 | 245 | *C. sinensis* | single | Fruit | Santa Cruz do Rio Pardo, SP, BR | commercial | 2018 | SJP | BR_SP_SCP02 | *p29* | NS |
|  |  |  |  |  |  |  |  |  |  | *p24* | NS |
| 373 | 246 | *C. sinensis* | single | Fruit | Santa Cruz do Rio Pardo, SP, BR | commercial | 2018 | SJP | BR_SP_SCP03 | *p29* | NS |
|  |  |  |  |  |  |  |  |  |  | *p24* | NS |
| 374 | 247 | *C. sinensis* (Pera) | single | Fruit | Santa Rosa de Viterbo, SP, BR | commercial | 2018 | CRD | BR_SP_SRV01 | *p29* | NS |
|  |  |  |  |  |  |  |  |  |  | *p24* | NS |
| 375 | 248 | *C. sinensis* (Pera) | single | Fruit | Santa Salete, SP, BR | commercial | 2018 | SJP | BR_SP_SnS01 | *p29* | NS |
|  |  |  |  |  |  |  |  |  |  | *p24* | NS |
| 376 | 249 | *C. sinensis* | single | Fruit | Santa Salete, SP, BR | commercial | 2018 | SJP | BR_SP_SnS02 | *p29* | NS |
|  |  |  |  |  |  |  |  |  |  | *p24* | NS |
| 377 | 250 | *C. sinensis* | single | Fruit | Santa Salete, SP, BR | commercial | 2018 | SJP | BR_SP_SnS03 | *p29* | NS |
|  |  |  |  |  |  |  |  |  |  | *p24* | NS |
| 378 | 251 | *C. sinensis* | single | Fruit | São Carlos, SP, BR | commercial | 2018 | CRD+SJP | BR_SP_SaC01 | *p29* | NS |
|  |  |  |  |  |  |  |  |  |  | *p24* | NS |
|  |  |  |  |  |  |  |  |  | BR_SP_SaC02 | *p29* | NS |
|  |  |  |  |  |  |  |  |  |  | *p24* | NS |
| 379 | 252 | *C. sinensis* | single | Fruit | São Carlos, SP, BR | commercial | 2018 | SJP | BR_SP_SaC02 | *p29* | NS |
|  |  |  |  |  |  |  |  |  |  | *p24* | NS |
| 380 | 253 | *C. sinensis* (Pera) | single | Fruit | São João da Boa Vista, SP, BR | commercial | 2018 | SJP | BR_SP_SJV01 | *p29* | NS |
|  |  |  |  |  |  |  |  |  |  | *p24* | NS |
| 381 | 254 | *C. sinensis* (Pera) | single | Fruit | São João da Boa Vista, SP, BR | commercial | 2018 | CRD | BR_SP_SJV02 | *p29* | NS |
|  |  |  |  |  |  |  |  |  |  | *p24* | NS |
| 382 | 255 | *C. sinensis* | single | Fruit | São João da Boa Vista, SP, BR | commercial | 2018 | CRD | BR_SP_SJV03 | *p29* | NS |
|  |  |  |  |  |  |  |  |  |  | *p24* | NS |
| 383 | 256 | *C. sinensis* | single | Fruit | São João da Boa Vista, SP, BR | commercial | 2018 | SJP | BR_SP_SJV04 | *p29* | NS |
|  |  |  |  |  |  |  |  |  |  | *p24* | NS |
| 384 | 257 | *C. sinensis* | single | Fruit | São Pedro da União, MG, BR | commercial | 2018 | SJP | BR_MG_SPU01 | *p29* | NS |
|  |  |  |  |  |  |  |  |  |  | *p24* | NS |
| 385 | 258 | *C. sinensis* (Natal) | single | Fruit | São Pedro da União, MG, BR | commercial | 2018 | SJP | BR_MG_SPU02 | *p29* | NS |
|  |  |  |  |  |  |  |  |  |  | *p24* | NS |
| 386 | 259 | *C. sinensis* (Natal) | single | Fruit | São Pedro, SP, BR | commercial | 2018 | CRD+SJP | BR_SP_SPe01 | *p29* | NS |
|  |  |  |  |  |  |  |  |  |  | *p24* | NS |
|  |  |  |  |  |  |  |  |  | BR_SP_SPe02 | *p29* | NS |
|  |  |  |  |  |  |  |  |  |  | *p24* | NS |
| 377 | 260 | *C. sinensis* (Natal) | single | Fruit | São Pedro, SP, BR | commercial | 2018 | SJP | BR_SP_SPe03 | *p29* | NS |
|  |  |  |  |  |  |  |  |  |  | *p24* | NS |
| 388 | 261 | *C. sinensis* | single | Fruit | São Pedro, SP, BR | commercial | 2018 | SJP | BR_SP_SPe04 | *p29* | NS |
|  |  |  |  |  |  |  |  |  |  | *p24* | NS |
| 389 | 262 | *C. sinensis* | single | Fruit | São Pedro, SP, BR | commercial | 2018 | SJP | BR_SP_SPe05 | *p29* | NS |
|  |  |  |  |  |  |  |  |  |  | *p24* | NS |
| 390 | 263 | *C. sinensis* (Pera) | single | Fruit | São Pedro, SP, BR | commercial | 2018 | CRD+SJP | BR_SP_SPe06 | *p29* | NS |
|  |  |  |  |  |  |  |  |  |  | *p24* | NS |
|  |  |  |  |  |  |  |  |  | BR_SP_SPe07 | *p29* | NS |
|  |  |  |  |  |  |  |  |  |  | *p24* | NS |
| 391 | 265 | *C. sinensis* (Natal) | single | Fruit | São Sebastião do Paraíso, MG, BR | commercial | 2018 | SJP | BR_MG_SSP01 | *p29* | NS |
|  |  |  |  |  |  |  |  |  |  | *p24* | NS |
| 392 | 266 | *C. sinensis* | single | Fruit | São Simão, SP, BR | commercial | 2018 | SJP | BR_SP_SaS01 | *p29* | NS |
|  |  |  |  |  |  |  |  |  |  | *p24* | NS |
| 393 | 267 | *C. sinensis* | single | Leaf | Sinop, MT, BR | commercial | 2019 | CRD | BR_MT_Snp01 | *p29* | NS |
|  |  |  |  |  |  |  |  |  |  | *p24* | NS |
| 394 | 268 | *C. sinensis* | single | Fruit | Sud Mennucci, SP, BR | commercial | 2018 | CRD+SJP | BR_SP_SdM13 | *p29* | NS |
|  |  |  |  |  |  |  |  |  |  | *p24* | NS |
|  |  |  |  |  |  |  |  |  | BR_SP_SdM14 | *p29* | NS |
|  |  |  |  |  |  |  |  |  |  | *p24* | NS |
| 395 | 269 | *C. sinensis* | single | Fruit | Sud Mennucci, SP, BR | commercial | 2018 | SJP | BR_SP_SdM15 | RNA1 | MW574403 |
|  |  |  |  |  |  |  |  |  |  | RNA2 | MW574408 |
| 396 | 270 | *C. sinensis* | single | Fruit | Tabapuã, SP, BR | commercial | 2018 | SJP | BR_SP_Tbp01 | *p29* | NS |
|  |  |  |  |  |  |  |  |  |  | *p24* | NS |
| 397 | 271 | *C. sinensis* | single | Fruit | Tabatinga, SP, BR | commercial | 2018 | SJP | BR_SP_Tbt02 | *p29* | NS |
|  |  |  |  |  |  |  |  |  |  | *p24* | NS |
| 398 | 272 | *C. sinensis* | single | Fruit | Tabatinga, SP, BR | commercial | 2018 | SJP | BR_SP_Tbt03 | *p29* | NS |
|  |  |  |  |  |  |  |  |  |  | *p24* | NS |
| 399 | 273 | *C. sinensis* | single | Fruit | Tabatinga, SP, BR | commercial | 2018 | SJP | BR_SP_Tbt04 | *p29* | NS |
|  |  |  |  |  |  |  |  |  |  | *p24* | NS |
| 400 | 274 | *C. sinensis* | single | Fruit | Tambaú, SP, BR | commercial | 2018 | SJP | BR_SP_Tmb34 | *p29* | NS |
|  |  |  |  |  |  |  |  |  |  | *p24* | NS |
| 401 | 275 | *C. sinensis* (Pera) | single | Fruit | Tanabi, SP, BR | commercial | 2018 | SJP | BR_SP_Tnb01 | *p29* | NS |
|  |  |  |  |  |  |  |  |  |  | *p24* | NS |
| 402 | 276 | *C. sinensis* (Pera) | single | Fruit | Tanabi, SP, BR | commercial | 2018 | CRD+SJP | BR_SP_Tnb02 | *p29* | NS |
|  |  |  |  |  |  |  |  |  |  | *p24* | NS |
|  |  |  |  |  |  |  |  |  | BR_SP_Tnb03 | *p29* | NS |
|  |  |  |  |  |  |  |  |  |  | *p24* | NS |
| 403 | 277 | *C. sinensis* (Natal) | single | Fruit | Taquaritinga, SP, BR | commercial | 2018 | SJP | BR_SP_Tqt05 | *p29* | NS |
|  |  |  |  |  |  |  |  |  |  | *p24* | NS |
| 404 | 278 | *C. sinensis* (Pera) | single | Fruit | Tatuí, SP, BR | commercial | 2018 | CRD | BR_SP_Tti03 | *p29* | NS |
|  |  |  |  |  |  |  |  |  |  | *p24* | NS |
| 405 | 279 | *C. sinensis* (Pera) | single | Fruit | Trabiju, SP, BR | commercial | 2018 | SJP | BR_SP_Trb01 | *p29* | NS |
|  |  |  |  |  |  |  |  |  |  | *p24* | NS |
| 406 | 280 | *C. sinensis* (Pera) | single | Fruit | Turmalina, SP, BR | commercial | 2018 | CRD+SJP | BR_SP_Trm01 | *p29* | NS |
|  |  |  |  |  |  |  |  |  |  | *p24* | NS |
|  |  |  |  |  |  |  |  |  | BR_SP_Trm02 | *p29* | NS |
|  |  |  |  |  |  |  |  |  |  | *p24* | NS |
| 407 | 281 | *C. sinensis* (Pera) | single | Fruit | Ubirajara, SP, BR | commercial | 2018 | CRD+SJP | BR_SP_Ubj01 | *p29* | NS |
|  |  |  |  |  |  |  |  |  |  | *p24* | NS |
|  |  |  |  |  |  |  |  |  | BR_SP_Ubj02 | *p29* | NS |
|  |  |  |  |  |  |  |  |  |  | *p24* | NS |
| 408 | 282 | *C. sinensis* (Natal) | single | Fruit | Ubirajara, SP, BR | commercial | 2018 | SJP | BR_SP_Ubj02 | *p29* | NS |
|  |  |  |  |  |  |  |  |  |  | *p24* | NS |
| 409 | 283 | *C. sinensis* (Natal) | single | Fruit | Uru, SP, BR | commercial | 2018 | SJP | BR_SP_Uru02 | *p29* | NS |
|  |  |  |  |  |  |  |  |  |  | *p24* | NS |
| 410 | 284 | *C. sinensis* (Natal) | single | Fruit | Uru, SP, BR | commercial | 2018 | SJP | BR_SP_Uru03 | *p29* | NS |
|  |  |  |  |  |  |  |  |  |  | *p24* | NS |
| 411 | 285 | *C. sinensis* (Pera) | single | Fruit | Uru, SP, BR | commercial | 2018 | SJP | BR_SP_Uru04 | *p29* | NS |
|  |  |  |  |  |  |  |  |  |  | *p24* | NS |
| 412 | 286 | *C. sinensis* (Pera) | single | Fruit | Uru, SP, BR | commercial | 2018 | SJP | BR_SP_Uru05 | *p29* | NS |
|  |  |  |  |  |  |  |  |  |  | *p24* | NS |
| 413 | 287 | *C. sinensis* | pool | Fruit | Vista Alegre do Alto, SP, BR | commercial | 2018 | SJP | BR_SP_VAA01 | *p29* | NS |
|  |  |  |  |  |  |  |  |  |  | *p24* | NS |
| 414 | 288 | *C. sinensis* | single | Leaf | Vitoria, ES, BR | non-commercial | 2018 | CRD | BR_ES_Vtr01 | RNA1 | MT554539 |
|  |  |  |  |  |  |  |  |  |  | RNA2 | MT554553 |
| 415 | 289 | *C. sinensis* | single | Leaf | Bebedouro, SP, BR | commercial | 2019 | SJP | BR_SP_Beb34 | *p29* | NS |
|  |  |  |  |  |  |  |  |  |  | *p24* | NS |
| 416 | 290 | *C. sinensis* | single | Leaf | Bebedouro, SP, BR | commercial | 2019 | SJP | BR_SP_Beb35 | *p29* | NS |
|  |  |  |  |  |  |  |  |  |  | *p24* | NS |
| 417 | 291 | *C. sinensis* | single | Leaf | Bebedouro, SP, BR | commercial | 2019 | SJP | BR_SP_Beb36 | *p29* | NS |
|  |  |  |  |  |  |  |  |  |  | *p24* | NS |
| 418 | 292 | *C. sinensis* | single | Fruit | Bebedouro, SP, BR | commercial | 2019 | SJP | BR_SP_Beb37 | *p29* | NS |
|  |  |  |  |  |  |  |  |  |  | *p24* | NS |
| 419 | 293 | *C. sinensis* (Valencia) | pool | Fruit, leaf and branch | Jaboticabal, SP, BR | commercial | 2019 | SJP | BR_SP_Jbt04 | *p29* | NS |
|  |  |  |  |  |  |  |  |  |  | *p24* | NS |
| 420 | 294 | *C. sinensis* | single | Leaf | Caazapa, PY | non-commercial | 2019 | CRD | PY_Czp01 | *p29* | NS |
|  |  |  |  |  |  |  |  |  |  | *p24* | NS |
| 421 | 295 | *C. sinensis* | single | Fruit | Braganca Paulista, SP, BR | non-commercial | 2019 | CRD | BR_SP_BgP01 | *p29* | NS |
|  |  |  |  |  |  |  |  |  |  | *p24* | NS |
| 422 | 296 | *C. sinensis* | single | Leaf | Braganca Paulista, SP, BR | non-commercial | 2019 | CRD | BR_SP_BgP02 | *p29* | NS |
|  |  |  |  |  |  |  |  |  |  | *p24* | NS |
| 423 | 297 | *C. sinensis* | single | Branch | Braganca Paulista, SP, BR | non-commercial | 2019 | CRD | BR_SP_BgP03 | *p29* | NS |
|  |  |  |  |  |  |  |  |  |  | *p24* | NS |
| 424 | 298 | *C. sinensis* | pool | Leaf | Bella Vista, Corrientes, AR | non-commercial | 2019 | CRD | AR13 | *p29* | NS |
|  |  |  |  |  |  |  |  |  |  | *p24* | NS |
| 425 | 299 | *C. sinensis* (Valencia) | pool | Leaf | Bella Vista, Corrientes, AR | non-commercial | 2019 | CRD | AR14 | *p29* | NS |
|  |  |  |  |  |  |  |  |  |  | *p24* | NS |
| 426 | 300 | *C. sinensis* (Hamlim) | pool | Leaf | Bella Vista, Corrientes, AR | non-commercial | 2019 | CRD | AR15 | *p29* | NS |
|  |  |  |  |  |  |  |  |  |  | *p24* | NS |
| 427 | 301 | *C. sinensis* | single | Fruit | Capitão do Poço, PA, BR | Organic commercial | 2020 | CRD | BR_PA_CaP01 | RNA1 | MW574401 |
|  |  |  |  |  |  |  |  |  |  | RNA2 | MW574406 |
| 428 | 302 | *C. reticulata* | pool | Leaf | Jumirim, SP, BR | non-commercial | 2020 | CRD | BR_SP_Jmr01 | RNA1 | MW574402 |
|  |  |  |  |  |  |  |  |  |  | RNA2 | MW574407 |
| 429 | 303 | *C. sinensis* | single | Fruit | Limeira, SP, BR | commercial | 2020 | SJP | BR_SP_Lim09 | RNA1 | MW574404 |
|  |  |  |  |  |  |  |  |  |  | RNA2 | MW574409 |
| 430 | 304 | *C. sinensis* | single | Fruit | Santo Antônio da Posse, SP, BR | commercial | 2020 | SJP | BR_SP_SAP03 | RNA1 | MW574405 |
|  |  |  |  |  |  |  |  |  |  | RNA2 | MW574410 |

^a^Viruses were detected from a pool of lesions, a unique lesion, or a single lesion; ^b^ Country abbreviations, BR: Brazil, AR: Argentina, CO: Colombia, and PY: Paraguay; Brazilian states or district abbreviations, AM: Amazonas, DF: Distrito Federal; MG: Minas Gerais, MS: Mato Grosso do Sul, PA: Pará; PR: Paraná, RJ: Rio de Janeiro; RS: Rio Grande do Sul; SE: Sergipe, SP: São Paulo and TO: Tocantins; ^c^The presence of CiLV-C was assessed by RT-PCR using primers for the detection of different regions of the genome (*p29*, *p15*, *p32* or *p24*) and/or high throughput sequencing (HTS). Isolates were identified according to the phylogenetic clade they belong to. ^d^The detected CiLV-C isolates were classified according to the collection site (Country / State / City) followed by numerical order. ^e^R­NA1 and RNA2 indicate that the complete or near-complete genome is determined, ORF names indicate the region detected by PCR. ^f^NS indicate that amplicons were not sequenced or the sequences are not available. In addition to the sequences obtained in this study, other sequences of CiLV-C isolates were retrieved from GenBank and incorporated into the analyzed datasets. They are: AR01 and PY01 (Caceres et al., 2013); CO01 (Leon et al., 2006); MX01 (Castillo et al., 2011); PA01 (unpublished); BR_SP_Brm01 (Nunes et al., 2012); BR_SP_Crd01 (Locali et al., 2006); BR_SP_Jbt01 (Pascon et al., 2006) and AR02, BR_MGAfe_01, BR_SP_Amp01, BR_SE_Aju01, BR_SP_Ara01, BR_PA_Bel01, BR_SP_Brm02, BR_DF_Bsb01, BR_SP_Cmp01, BR_SP_CsB01, BR_SP_Cln01, BR_MG_CGz01, BR_SP_Cch01, BR_SP_Crd02, BR_SP_Crd03, BR_SP_Csm01, BR_GO_Gyn01, BR_MG_Lav01, BR_SC_Lsp01, BR_PR_Ldb01, BR_AM_Mao01, BR_PR_Mgf01, BR_SP_Mrn01, BR_SC_NCh01, BR_SC_NTb01, BR_TO_Pmw01, BR_SP_Prb01, BR_GO_Pnt01, BR_SP_Prt01, BR_AC_RBr01, BR_SP_Itu01, BR_MG_Stc01, BR_SP_SAP01, BR_SP_SNg01, BR_SP_SJP01, BR_SP_SJP02, BR_SP_SJP03, BR_SP_SJP04, BR_SP_SJP05, BR_SP_SdM01, BR_SP_SdM02, BR_SP_SdM_03, BR_SP_SdM_04, BR_SP_SdM_05, BR_SP_SdM_06, BR_RJ_Tng01, BR_SP_Tti01, BR_MT_Trn01, BR_MG_Uba01 (Ramos-González et al., 2016).

**Supplementary Table S2.** Nucleotide and haplotypic diversities of ORFs *p29* and *p32* of CiLV-C. Nucleotide sequences were amplified and cloned from samples of infected *Citrus sinensis* trees collected in commercial orchards of the citrus belt São Paulo - Minas Gerais, Brazil, in 2017.

| **Sample collection location** | ***p29*** | | | ***p32*** | | | **Number of isolates selected for the concatenate analysis** |
| --- | --- | --- | --- | --- | --- | --- | --- |
|  | **Number of** | | **Nucleotide diversity (π)** | **Number of** | | **Nucleotide diversity (π)** |  |
|  | **Isolates** | **Haplotypes** |  | **Isolates** | **Haplotypes** |  |  |
| Aguaí, SP | 4 | 4 | 0.00440 | 9 | 4 | 0.00752 | **4** |
| Altinópolis, SP | 3 | 3 | 0.00252 | 6 | 3 | 0.00231 | **3** |
| Barretos, SP | 10 | 7 | 0.00579 | 16 | 4 | 0.00749 | **7** |
| Bebedouro, SP | 7 | 6 | 0.00252 | 31 | 6 | 0.00175 | **7** |
| Brotas, SP | 7 | 7 | 0.00276 | 11 | 5 | 0.00795 | **7** |
| Cerqueira Cesar, SP | 8 | 7 | 0.00557 | 11 | 6 | 0.00568 | **7** |
| Guaimbê, SP | 2 | 2 | 0.00503 | 8 | 3 | 0.00260 | **2** |
| Mogi Mirim, SP | 12 | 11 | 0.00496 | 27 | 7 | 0.00785 | **11** |
| Pirassununga, SP | 3 | 3 | 0.00252 | 4 | 2 | 0.00174 | **3** |
| Santa Maria da Serra, SP | 7 | 7 | 0.00288 | 2 | 2 | 0.00347 | **2** |
| Sud Mennucci, SP | 12 | 6 | 0.00739 | 6 | 3 | 0.00301 | **6** |
| Tambaú, SP | 8 | 6 | 0.00299 | 8 | 3 | 0.00260 | **6** |
| Taquaral, SP | 3 | 3 | 0.00168 | 7 | 4 | 0.00364 | **3** |
| Taquaritinga, SP | 2 | 1 | 0 | 4 | 1 | 0 | **1** |
| Uberaba, MG | 9 | 9 | 0.00538 | 15 | 7 | 0.00370 | **9** |

| **CiLV-C isolates** | **CiLV-C_BR_SP_Crd01** | | | | | | | | | | | | | | |
| --- | --- | --- | --- | --- | --- | --- | --- | --- | --- | --- | --- | --- | --- | --- | --- |
|  | **RNA1** | ***RdRp*** | | ***p29*** | | **RNA2** | ***p15*** | | ***IR*** | ***p61*** | | ***p32*** | | ***p24*** | |
|  | nt | nt | aa | nt | aa | nt | nt | aa | nt | nt | aa | nt | aa | nt | aa |
| AR04 | 99 | 99 | 99 | 99 | 100 | 98 | 99 | 98 | 98 | 98 | 98 | 99 | 99 | 99 | 100 |
| AR05 | 99 | 99 | 99 | 99 | 100 | 98 | 98 | 97 | 97 | 99 | 99 | 99 | 99 | 99 | 100 |
| AR06 | 99 | 99 | 99 | 99 | 100 | 99 | 99 | 100 | 99 | 99 | 99 | 99 | 100 | 99 | 100 |
| BR_ES_Vtr01 | 99 | 99 | 99 | 99 | 100 | 99 | 99 | 100 | 99 | 99 | 99 | 99 | 100 | 99 | 100 |
| BR_PA_CaP01 | 99 | 99 | 99 | 99 | 100 | 99 | 99 | 100 | 99 | 99 | 99 | 99 | 99 | 99 | 100 |
| BR_RS_Urg01 | 99 | 99 | 99 | 99 | 100 | 99 | 99 | 99 | 98 | 99 | 99 | 99 | 99 | 99 | 100 |
| BR_SP_Jac01 | 98 | 98 | 99 | 99 | 100 | 99 | 99 | 99 | 99 | 99 | 100 | 99 | 100 | 99 | 100 |
| BR_SP_Jbt02 | 99 | 99 | 99 | 99 | 100 | 99 | 100 | 100 | 99 | 99 | 100 | 99 | 99 | 99 | 100 |
| BR_SP_Jmr01 | 99 | 99 | 99 | 99 | 100 | 99 | 99 | 99 | 99 | 99 | 99 | 99 | 99 | 99 | 100 |
| BR_SP_Lim01 | 99 | 99 | 99 | 99 | 100 | 99 | 100 | 99 | 99 | 99 | 100 | 99 | 100 | 99 | 100 |
| BR_SP_Lim09 | 86 | 86 | 93 | 85 | 90 | 88 | 99 | 99 | 96 | 82 | 84 | 87 | 92 | 88 | 94 |
| BR_SP_Prb02 | 99 | 99 | 99 | 99 | 100 | 99 | 99 | 99 | 99 | 99 | 100 | 99 | 100 | 99 | 100 |
| BR_SP_Prb03 | 99 | 99 | 99 | 99 | 100 | 99 | 100 | 100 | 99 | 99 | 99 | 99 | 100 | 99 | 100 |
| BR_SP_Prb04 | 99 | 99 | 99 | 99 | 100 | 99 | 100 | 100 | 99 | 99 | 100 | 99 | 95 | 100 | 100 |
| BR_SP_SAP03 | 86 | 86 | 93 | 85 | 90 | 88 | 99 | 99 | 96 | 82 | 84 | 87 | 92 | 88 | 94 |
| BR_SP_SdM15 | 86 | 86 | 93 | 85 | 90 | 88 | 99 | 99 | 96 | 82 | 84 | 87 | 92 | 88 | 94 |
| BR_SP_SJP05 | 86 | 86 | 93 | 85 | 90 | 89 | 99 | 99 | 97 | 82 | 84 | 87 | 92 | 88 | 94 |
| BR_SP_SPa11 | 99 | 99 | 99 | 99 | 100 | 99 | 99 | 100 | 99 | 99 | 99 | 100 | 100 | 100 | 100 |
| PY_Asu02 | 86 | 86 | 93 | 86 | 88 | 86 | 98 | 100 | 85 | 82 | 81 | 89 | 95 | 89 | 94 |
|  | **CiLV-C_BR_SP_SJP01** | | | | | | | | | | | | | | |
|  | **RNA1** | ***RdRp*** | | ***p29*** | | **RNA2** | ***p15*** | | **IR** | ***p61*** | | ***p32*** | | ***p24*** | |
|  | nt | nt | aa | nt | aa | nt | nt | aa | nt | nt | aa | nt | aa | nt | aa |
| AR04 | 88 | 85 | 93 | 85 | 90 | 88 | 99 | 98 | 96 | 82 | 85 | 87 | 92 | 87 | 93 |
| AR05 | 88 | 85 | 93 | 85 | 90 | 88 | 98 | 97 | 95 | 82 | 84 | 87 | 92 | 88 | 93 |
| AR06 | 88 | 85 | 93 | 85 | 90 | 88 | 99 | 100 | 97 | 82 | 84 | 87 | 92 | 88 | 93 |
| BR_ES_Vtr01 | 88 | 85 | 93 | 85 | 90 | 88 | 99 | 100 | 97 | 82 | 84 | 87 | 92 | 87 | 93 |
| BR_RS_Urg01 | 88 | 85 | 93 | 84 | 90 | 88 | 99 | 99 | 97 | 82 | 84 | 86 | 92 | 88 | 93 |
| BR_PA_CaP01 | 88 | 85 | 93 | 85 | 90 | 88 | 99 | 100 | 97 | 82 | 84 | 99 | 92 | 88 | 93 |
| BR_SP_Jac01 | 88 | 85 | 92 | 85 | 90 | 88 | 99 | 99 | 97 | 82 | 84 | 87 | 92 | 87 | 93 |
| BR_SP_Jbt02 | 88 | 86 | 93 | 85 | 90 | 88 | 99 | 100 | 97 | 82 | 84 | 87 | 92 | 87 | 93 |
| BR_SP_Jmr01 | 87 | 85 | 93 | 85 | 90 | 88 | 99 | 99 | 97 | 82 | 84 | 99 | 92 | 88 | 93 |
| BR_SP_Lim01 | 88 | 86 | 93 | 85 | 90 | 88 | 99 | 99 | 97 | 82 | 84 | 87 | 92 | 87 | 93 |
| BR_SP_Lim09 | 99 | 99 | 99 | 99 | 99 | 99 | 99 | 99 | 99 | 99 | 99 | 87 | 99 | 99 | 100 |
| BR_SP_Prb02 | 88 | 85 | 93 | 85 | 90 | 88 | 99 | 99 | 97 | 82 | 84 | 87 | 92 | 88 | 93 |
| BR_SP_Prb03 | 88 | 85 | 93 | 85 | 90 | 88 | 99 | 100 | 97 | 81 | 83 | 87 | 92 | 88 | 93 |
| BR_SP_Prb04 | 88 | 86 | 93 | 85 | 90 | 88 | 99 | 100 | 97 | 82 | 84 | 87 | 92 | 87 | 93 |
| BR_SP_SAP03 | 99 | 99 | 99 | 99 | 99 | 99 | 99 | 100 | 99 | 99 | 99 | 87 | 99 | 99 | 100 |
| BR_SP_SdM15 | 99 | 99 | 99 | 99 | 99 | 99 | 99 | 99 | 99 | 99 | 99 | 87 | 99 | 99 | 100 |
| BR_SP_SJP05 | 99 | 99 | 99 | 99 | 99 | 99 | 99 | 100 | 100 | 99 | 99 | 99 | 99 | 99 | 100 |
| BR_SP_SPa11 | 88 | 85 | 93 | 85 | 90 | 88 | 99 | 100 | 97 | 82 | 84 | 87 | 92 | 87 | 93 |
| PY_Asu02 | 85 | 88 | 95 | 86 | 90 | 85 | 98 | 100 | 85 | 83 | 84 | 87 | 94 | 89 | 94 |

**Supplementary Table S3.** Nucleotide and deduced amino acid identities (%) among CiLV-C isolates described in this study and the type members of the clades CRD (CiLV-C_BR_SP_Crd01, GenBank accession numbers DQ352194 and DQ352195) and SJP (CiLV-C_BR_SP_SJP01, GB accession numbers KP336746 and KP336747).

**Supplementary Table S4**. Recombination events detected in CiLV-C sequences determined by RDP software version 5.5.

| **Event** | **Recombinant strain** | **Major parent** | **Minor parent** | **Detection method^a^** | | | | | | | **Highest**  ***p*-value** |
| --- | --- | --- | --- | --- | --- | --- | --- | --- | --- | --- | --- |
|  |  |  |  | R | G | B | M | C | S | T |  |
| **RNA2 (n=23)** | | | | | | | | | | | |
| 1 | BR_SP_SJP01 | Unknown | BR_SP_SPa11 | + | + | + | + | + | + | + | 1.387 x 10^-17^ |
|  | BR_SP_SJP05 |  |  |  |  |  |  |  |  |  |  |
|  | BR_SP_Lim09 |  |  |  |  |  |  |  |  |  |  |
|  | BR_SP_SAP03 |  |  |  |  |  |  |  |  |  |  |
|  | BR_SP_SdM15 |  |  |  |  |  |  |  |  |  |  |
| 2 | PY_Asu02 | Unknown | BR_RS_Urg01 | + | + | + | + | + | + | + | 1.607 x 10^-15^ |
| 3 | BR_SP_SJP01 | BR-SP_Lim09 | Unknown | + | + | + | + | + | + | + | 3.933 x 10^-18^ |
| **RNA2: *p15*-IR-*p32* (n=56)** | | | | | | | | | | | |
| 4 | BR_SP_Csm01 | Unknown | BR_PA_Bel01 | - | + | + | + | + | + | + | 9.656 x 10^-08^ |
|  | BR_SP_Lim09 |  |  |  |  |  |  |  |  |  |  |
|  | BR_SP_SJP01 |  |  |  |  |  |  |  |  |  |  |
|  | BR_SP_SJP02 |  |  |  |  |  |  |  |  |  |  |
|  | BR_SP_SJP05 |  |  |  |  |  |  |  |  |  |  |
|  | BR_SP_SdM01 |  |  |  |  |  |  |  |  |  |  |
|  | BR_SP_SdM02 |  |  |  |  |  |  |  |  |  |  |
|  | BR_SP_SdM03 |  |  |  |  |  |  |  |  |  |  |
|  | BR_SP_SdM04 |  |  |  |  |  |  |  |  |  |  |
|  | BR_SP_SdM05 |  |  |  |  |  |  |  |  |  |  |
|  | BR_SP_SdM06 |  |  |  |  |  |  |  |  |  |  |
|  | BR_SP_SdM15 |  |  |  |  |  |  |  |  |  |  |
| 5 | BR_SP_SAP03 | Unknown | BR_PA_Bel01 | - | - | - | + | + | + | + | 2.631 x 10-9 |
| 6 | BR_PR_Mgf01 | Unknown | AR05 | - | + | + | + | + | + | + | 1.039 x 10^-12^ |
| 7 | PY02 | Unknown | BR_PR_Ldb01 | - | - | - | + | + | + | + | 7.689 x 10^-7^ |

^a^Method abbreviation = R: RDP; G: GENECONV; B: Bootscan; M: Maxchi; C: Chimaera; S: SiScan; and T: Topal.

**Supplementary Table S5**. Summary of selection analysis of the *p29* and *p32* in CiLV-C.

| **Method** | **Dataset** | **Selection pressure** | ***p*-value** | **Number of sites** | **Amino acid under selection** |
| --- | --- | --- | --- | --- | --- |
| ***p29*** | | | | | |
| FUBAR | SJP+CRD+ASU (190) | purification | 0.9 | 47 | 5, 7, 29, 30, 32, 33, 47, 58, 61, 69, 76, 99, 100, 101, 106, 112, 113, 116, 136, 137, 138, 139, 154, 157, 161, 165, 171, 173, 175, 181, 195, 196, 198, 201, 207, 208, 209, 213, 217, 219, 220, 227, 240, 244, 245, 254 and 259 |
|  | SJP+CRD (189) | purification | 0.9 | 45 | 5,7, 12, 29, 30, 32, 33, 48, 58, 61, 76, 100, 101, 106, 112, 113, 116, 136, 137, 139, 151, 154, 157, 161, 165, 173, 181, 195, 196, 198, 201, 202, 207, 208, 209, 213, 217, 219, 227, 234, 240, 244, 245, 254 and 259 |
|  | SJP (106) | purification | 0.9 | 17 | 7, 29, 48, 61, 68, 69, 76, 101, 137, 139, 157, 173, 198, 208, 219, 240 and 244 |
|  |  | diversification | 0.9 | 1 | 16 |
|  | CRD (83) | purification | 0.9 | 13 | 7, 32, 58, 99, 105, 111, 134, 159, 169, 179, 209, 215 and 225 |
| FEL | SJP+CRD+ASU (190) | purification | 0.1 | 53 | 7, 15, 23, 29, 30, 32, 33, 47, 58, 61, 69, 76, 83, 99, 100, 101, 106, 113, 116, 117, 136, 137, 138, 139, 147, 154, 157, 159, 161, 165, 173, 175, 181, 186, 194, 195, 196, 198, 201, 207, 208, 209, 211, 213, 217, 219, 220, 227, 240, 245, 251, 254 and 259 |
|  | SJP+CRD (189) | purification | 0.1 | 48 | 5, 7, 12, 13, 15, 29, 30, 32, 33, 58, 61, 76, 100, 101, 106, 113, 116, 136, 137, 138, 139, 151, 154, 157, 159, 161, 165, 173, 181, 195, 196, 198, 201, 202, 205, 207, 209, 211, 213, 217, 219, 220, 227, 234, 240, 245, 254 and 259 |
|  |  | diversification | 0.1 | 1 | 93 |
|  | SJP (106) | purification | 0.1 | 18 | 5, 7, 12, 29, 33, 38, 61, 68, 69, 92, 139, 157, 198, 209, 213, 234, 240 and 255 |
|  | CRD (83) | diversification | 0.1 | 1 | 149 |
|  |  | purification | 0.1 | 18 | 7, 32, 47, 58, 72, 87, 99, 101, 106, 107, 113, 136, 161, 167, 181, 211, 217 and 227 |
| MEME | SJP+CRD+ASU (190) | diversification | 0.1 | 6 | 16, 26, 53, 104, 140 and 163 |
|  | SJP+CRD (189) | diversification | 0.1 | 7 | 16, 26, 53, 93, 104, 140 and 163 |
|  | SJP (106) | diversification | 0.1 | 2 | 16 and 53 |
|  | CRD (83) | diversification | 0.1 | 3 | 26, 102 and 138 |
| FUBAR_FEL | SJP+CRD+ASU (190) | purification | 0.1 | 42 | 7, 29, 30, 32, 33, 47, 58, 61, 69,76, 99, 100, 101, 106, 113, 116, 136, 137, 138, 139, 154, 157, 161, 165, 173, 175, 181, 195, 196, 198, 201, 207, 208, 209, 213, 217, 219, 220, 227, 240, 245, 254 and 259 |
|  | SJP+CRD (189) | purification | 0.1 | 42 | 5,7, 12, 29, 30, 32, 33, 58, 61, 76, 100, 101, 106, 113, 116, 136, 137, 139, 151, 154, 157, 161, 165, 173, 181, 195, 196, 198, 201, 202, 207, 208, 209, 213, 217, 219, 227, 234, 240, 245, 254 and 259 |
|  | SJP (106) | purification | 0.1 | 9 | 7, 29, 61, 68, 69, 139, 157, 198 and 240 |
|  | CRD (83) | purification | 0.1 | 4 | 7, 32, 58 and 99 |
| ***p32*^a^** | | | | | |
| FUBAR | SJP+CRD+ASU (270) | purification | 0.9 | 24 | 68, 71, 72, 73, 74, 75, 76, 77, 80, 84, 93, 95, 96, 97, 98, 99, 103, 111, 112, 115, 138, 146, 147 and 148 |
|  | SJP+CRD (269) | purification | 0.9 | 19 | 68, 71, 72, 75, 76, 80, 84, 93, 96, 97, 98, 99, 100, 101, 103, 115, 146, 147, and 148 |
|  | SJP (190) | purification | 0.9 | 1 | 75 |
|  | CRD (80) | purification | 0.9 | 5 | 75, 97, 103, 112 and 136 |
| FEL | SJP+CRD+ASU (270) | purification | 0.1 | 33 | 57, 68, 70, 71, 72, 73, 74, 75, 76, 77, 79, 80, 84, 93, 95, 96, 97, 98, 99, 102, 103, 111, 112, 115, 119, 121, 126, 138, 142, 145, 146, 147 and 148 |
|  | SJP+CRD (269) | purification | 0.1 | 27 | 57, 68, 71, 72, 74, 75, 76, 77, 80, 84, 85, 93, 96, 97, 98, 99, 102, 103, 111, 112, 115, 119, 121, 145, 146, 147 and 148 |
|  | SJP (190) | purification | 0.1 | 10 | 57, 64, 68, 72, 75, 84, 119, 125, 145 and 146 |
|  | CRD (80) | purification | 0.1 | 14 | 68, 69, 73, 75, 77, 85, 93, 96, 97, 98, 103, 136, 146 and 148 |
| MEME | SJP+CRD+ASU (270) | diversification | 0.1 | 2 | 64 |
|  | SJP+CRD (269) | diversification | 0.1 | 1 | 64 |
|  | SJP (190) | diversification | 0.1 | 0 | - |
|  | CRD (80) | diversification | 0.1 | 0 | - |
| FUBAR_FEL | SJP+CRD+ASU (270) | purification | 0.1 | 24 | 68, 71, 72, 73, 74, 75, 76, 77, 80, 84, 93, 95, 96, 97, 98, 99, 103, 111, 112, 115, 138, 146, 147 and 148 |
|  | SJP+CRD (269) | purification | 0.1 | 17 | 68, 71, 72, 75, 76, 80, 84, 93, 96, 97, 98, 99, 103, 115, 146, 147, and 148 |
|  | SJP (190) | purification | 0.1 | 1 | 75 |
|  | CRD (79) | purification | 0.1 | 4 | 75, 97, 103 and 136 |

^a^The analyzed sequence of the ORF *p32* corresponds to the amino acid stretch between positions 55 and 150 of the complete MP protein.
