# Supplementary material for: Molecular Epidemiology of Citrus Leprosis Virus C: A New Viral Lineage and Phylodynamic of the Main Viral Subpopulations in the Americas
Source: Front Microbiol. 2021 Apr 29;12:641252. doi: 10.3389/fmicb.2021.641252 (PMC8116597; doi:10.3389/fmicb.2021.641252)
Supplement: Supplementary Table 2 — Nucleotide and haplotypic diversities of ORFs p29 and p32 of CiLV-C. Nucleotide sequences were amplified and cloned from samples of infected Citrus sinensis trees collected in commercial orchards of the citrus belt São Paulo—Minas Gerais, Brazil, in 2017. [file Table_2.docx]

**Supplementary Table S2.** Nucleotide and haplotypic diversities of ORFs *p29* and *p32* of CiLV-C. Nucleotide sequences were amplified and cloned from samples of infected *Citrus sinensis* trees collected in commercial orchards of the citrus belt São Paulo - Minas Gerais, Brazil, in 2017.

| **Sample collection location** | ***p29*** | | | ***p32*** | | | **Number of isolates selected for the concatenate analysis** |
| --- | --- | --- | --- | --- | --- | --- | --- |
|  | **Number of** | | **Nucleotide diversity (π)** | **Number of** | | **Nucleotide diversity (π)** |  |
|  | **Isolates** | **Haplotypes** |  | **Isolates** | **Haplotypes** |  |  |
| Aguaí, SP | 4 | 4 | 0.00440 | 9 | 4 | 0.00752 | **4** |
| Altinópolis, SP | 3 | 3 | 0.00252 | 6 | 3 | 0.00231 | **3** |
| Barretos, SP | 10 | 7 | 0.00579 | 16 | 4 | 0.00749 | **7** |
| Bebedouro, SP | 7 | 6 | 0.00252 | 31 | 6 | 0.00175 | **7** |
| Brotas, SP | 7 | 7 | 0.00276 | 11 | 5 | 0.00795 | **7** |
| Cerqueira Cesar, SP | 8 | 7 | 0.00557 | 11 | 6 | 0.00568 | **7** |
| Guaimbê, SP | 2 | 2 | 0.00503 | 8 | 3 | 0.00260 | **2** |
| Mogi Mirim, SP | 12 | 11 | 0.00496 | 27 | 7 | 0.00785 | **11** |
| Pirassununga, SP | 3 | 3 | 0.00252 | 4 | 2 | 0.00174 | **3** |
| Santa Maria da Serra, SP | 7 | 7 | 0.00288 | 2 | 2 | 0.00347 | **2** |
| Sud Mennucci, SP | 12 | 6 | 0.00739 | 6 | 3 | 0.00301 | **6** |
| Tambaú, SP | 8 | 6 | 0.00299 | 8 | 3 | 0.00260 | **6** |
| Taquaral, SP | 3 | 3 | 0.00168 | 7 | 4 | 0.00364 | **3** |
| Taquaritinga, SP | 2 | 1 | 0 | 4 | 1 | 0 | **1** |
| Uberaba, MG | 9 | 9 | 0.00538 | 15 | 7 | 0.00370 | **9** |
