# Supplementary material for: Molecular Epidemiology of Citrus Leprosis Virus C: A New Viral Lineage and Phylodynamic of the Main Viral Subpopulations in the Americas
Source: Front Microbiol. 2021 Apr 29;12:641252. doi: 10.3389/fmicb.2021.641252 (PMC8116597; doi:10.3389/fmicb.2021.641252)
Supplement: Supplementary Table 3 — Nucleotide and deduced amino acid identities (%) among CiLV-C isolates described in this study and the type members of the clades CRD (CiLV-C_BR_SP_Crd01, GenBank accession numbers NC008169 and NC008170) and SJP (CiLV-C_BR_SP_SJP01, GB acc. numbers KP336746 and KP336747). [file Table_3.docx]

| **CiLV-C isolates** | **CiLV-C_BR_SP_Crd01** | | | | | | | | | | | | | | |
| --- | --- | --- | --- | --- | --- | --- | --- | --- | --- | --- | --- | --- | --- | --- | --- |
|  | **RNA1** | ***RdRp*** | | ***p29*** | | **RNA2** | ***p15*** | | ***IR*** | ***p61*** | | ***p32*** | | ***p24*** | |
|  | nt | nt | aa | nt | aa | nt | nt | aa | nt | nt | aa | nt | aa | nt | aa |
| AR04 | 99 | 99 | 99 | 99 | 100 | 98 | 99 | 98 | 98 | 98 | 98 | 99 | 99 | 99 | 100 |
| AR05 | 99 | 99 | 99 | 99 | 100 | 98 | 98 | 97 | 97 | 99 | 99 | 99 | 99 | 99 | 100 |
| AR06 | 99 | 99 | 99 | 99 | 100 | 99 | 99 | 100 | 99 | 99 | 99 | 99 | 100 | 99 | 100 |
| BR_ES_Vtr01 | 99 | 99 | 99 | 99 | 100 | 99 | 99 | 100 | 99 | 99 | 99 | 99 | 100 | 99 | 100 |
| BR_PA_CaP01 | 99 | 99 | 99 | 99 | 100 | 99 | 99 | 100 | 99 | 99 | 99 | 99 | 99 | 99 | 100 |
| BR_RS_Urg01 | 99 | 99 | 99 | 99 | 100 | 99 | 99 | 99 | 98 | 99 | 99 | 99 | 99 | 99 | 100 |
| BR_SP_Jac01 | 98 | 98 | 99 | 99 | 100 | 99 | 99 | 99 | 99 | 99 | 100 | 99 | 100 | 99 | 100 |
| BR_SP_Jbt02 | 99 | 99 | 99 | 99 | 100 | 99 | 100 | 100 | 99 | 99 | 100 | 99 | 99 | 99 | 100 |
| BR_SP_Jmr01 | 99 | 99 | 99 | 99 | 100 | 99 | 99 | 99 | 99 | 99 | 99 | 99 | 99 | 99 | 100 |
| BR_SP_Lim01 | 99 | 99 | 99 | 99 | 100 | 99 | 100 | 99 | 99 | 99 | 100 | 99 | 100 | 99 | 100 |
| BR_SP_Lim09 | 86 | 86 | 93 | 85 | 90 | 88 | 99 | 99 | 96 | 82 | 84 | 87 | 92 | 88 | 94 |
| BR_SP_Prb02 | 99 | 99 | 99 | 99 | 100 | 99 | 99 | 99 | 99 | 99 | 100 | 99 | 100 | 99 | 100 |
| BR_SP_Prb03 | 99 | 99 | 99 | 99 | 100 | 99 | 100 | 100 | 99 | 99 | 99 | 99 | 100 | 99 | 100 |
| BR_SP_Prb04 | 99 | 99 | 99 | 99 | 100 | 99 | 100 | 100 | 99 | 99 | 100 | 99 | 95 | 100 | 100 |
| BR_SP_SAP03 | 86 | 86 | 93 | 85 | 90 | 88 | 99 | 99 | 96 | 82 | 84 | 87 | 92 | 88 | 94 |
| BR_SP_SdM15 | 86 | 86 | 93 | 85 | 90 | 88 | 99 | 99 | 96 | 82 | 84 | 87 | 92 | 88 | 94 |
| BR_SP_SJP05 | 86 | 86 | 93 | 85 | 90 | 89 | 99 | 99 | 97 | 82 | 84 | 87 | 92 | 88 | 94 |
| BR_SP_SPa11 | 99 | 99 | 99 | 99 | 100 | 99 | 99 | 100 | 99 | 99 | 99 | 100 | 100 | 100 | 100 |
| PY_Asu02 | 86 | 86 | 93 | 86 | 88 | 86 | 98 | 100 | 85 | 82 | 81 | 89 | 95 | 89 | 94 |
|  | **CiLV-C_BR_SP_SJP01** | | | | | | | | | | | | | | |
|  | **RNA1** | ***RdRp*** | | ***p29*** | | **RNA2** | ***p15*** | | **IR** | ***p61*** | | ***p32*** | | ***p24*** | |
|  | nt | nt | aa | nt | aa | nt | nt | aa | nt | nt | aa | nt | aa | nt | aa |
| AR04 | 88 | 85 | 93 | 85 | 90 | 88 | 99 | 98 | 96 | 82 | 85 | 87 | 92 | 87 | 93 |
| AR05 | 88 | 85 | 93 | 85 | 90 | 88 | 98 | 97 | 95 | 82 | 84 | 87 | 92 | 88 | 93 |
| AR06 | 88 | 85 | 93 | 85 | 90 | 88 | 99 | 100 | 97 | 82 | 84 | 87 | 92 | 88 | 93 |
| BR_ES_Vtr01 | 88 | 85 | 93 | 85 | 90 | 88 | 99 | 100 | 97 | 82 | 84 | 87 | 92 | 87 | 93 |
| BR_RS_Urg01 | 88 | 85 | 93 | 84 | 90 | 88 | 99 | 99 | 97 | 82 | 84 | 86 | 92 | 88 | 93 |
| BR_PA_CaP01 | 88 | 85 | 93 | 85 | 90 | 88 | 99 | 100 | 97 | 82 | 84 | 99 | 92 | 88 | 93 |
| BR_SP_Jac01 | 88 | 85 | 92 | 85 | 90 | 88 | 99 | 99 | 97 | 82 | 84 | 87 | 92 | 87 | 93 |
| BR_SP_Jbt02 | 88 | 86 | 93 | 85 | 90 | 88 | 99 | 100 | 97 | 82 | 84 | 87 | 92 | 87 | 93 |
| BR_SP_Jmr01 | 87 | 85 | 93 | 85 | 90 | 88 | 99 | 99 | 97 | 82 | 84 | 99 | 92 | 88 | 93 |
| BR_SP_Lim01 | 88 | 86 | 93 | 85 | 90 | 88 | 99 | 99 | 97 | 82 | 84 | 87 | 92 | 87 | 93 |
| BR_SP_Lim09 | 99 | 99 | 99 | 99 | 99 | 99 | 99 | 99 | 99 | 99 | 99 | 87 | 99 | 99 | 100 |
| BR_SP_Prb02 | 88 | 85 | 93 | 85 | 90 | 88 | 99 | 99 | 97 | 82 | 84 | 87 | 92 | 88 | 93 |
| BR_SP_Prb03 | 88 | 85 | 93 | 85 | 90 | 88 | 99 | 100 | 97 | 81 | 83 | 87 | 92 | 88 | 93 |
| BR_SP_Prb04 | 88 | 86 | 93 | 85 | 90 | 88 | 99 | 100 | 97 | 82 | 84 | 87 | 92 | 87 | 93 |
| BR_SP_SAP03 | 99 | 99 | 99 | 99 | 99 | 99 | 99 | 100 | 99 | 99 | 99 | 87 | 99 | 99 | 100 |
| BR_SP_SdM15 | 99 | 99 | 99 | 99 | 99 | 99 | 99 | 99 | 99 | 99 | 99 | 87 | 99 | 99 | 100 |
| BR_SP_SJP05 | 99 | 99 | 99 | 99 | 99 | 99 | 99 | 100 | 100 | 99 | 99 | 99 | 99 | 99 | 100 |
| BR_SP_SPa11 | 88 | 85 | 93 | 85 | 90 | 88 | 99 | 100 | 97 | 82 | 84 | 87 | 92 | 87 | 93 |
| PY_Asu02 | 85 | 88 | 95 | 86 | 90 | 85 | 98 | 100 | 85 | 83 | 84 | 87 | 94 | 89 | 94 |

**Supplementary Table S3.** Nucleotide and deduced amino acid identities (%) among CiLV-C isolates described in this study and the type members of the clades CRD (CiLV-C_BR_SP_Crd01, GenBank accession numbers DQ352194 and DQ352195) and SJP (CiLV-C_BR_SP_SJP01, GB accession numbers KP336746 and KP336747).
