# Supplementary material for: Molecular Epidemiology of Citrus Leprosis Virus C: A New Viral Lineage and Phylodynamic of the Main Viral Subpopulations in the Americas
Source: Front Microbiol. 2021 Apr 29;12:641252. doi: 10.3389/fmicb.2021.641252 (PMC8116597; doi:10.3389/fmicb.2021.641252)
Supplement: Supplementary Table 4 — Recombination events detected in CiLV-C sequences determined by RDP software version 5.5. [file Table_4.docx]

**Supplementary Table S4**. Recombination events detected in CiLV-C sequences determined by RDP software version 5.5.

| **Event** | **Recombinant strain** | **Major parent** | **Minor parent** | **Detection method^a^** | | | | | | | **Highest**  ***p*-value** |
| --- | --- | --- | --- | --- | --- | --- | --- | --- | --- | --- | --- |
|  |  |  |  | R | G | B | M | C | S | T |  |
| **RNA2 (n=23)** | | | | | | | | | | | |
| 1 | BR_SP_SJP01 | Unknown | BR_SP_SPa11 | + | + | + | + | + | + | + | 1.387 x 10^-17^ |
|  | BR_SP_SJP05 |  |  |  |  |  |  |  |  |  |  |
|  | BR_SP_Lim09 |  |  |  |  |  |  |  |  |  |  |
|  | BR_SP_SAP03 |  |  |  |  |  |  |  |  |  |  |
|  | BR_SP_SdM15 |  |  |  |  |  |  |  |  |  |  |
| 2 | PY_Asu02 | Unknown | BR_RS_Urg01 | + | + | + | + | + | + | + | 1.607 x 10^-15^ |
| 3 | BR_SP_SJP01 | BR-SP_Lim09 | Unknown | + | + | + | + | + | + | + | 3.933 x 10^-18^ |
| **RNA2: *p15*-IR-*p32* (n=56)** | | | | | | | | | | | |
| 4 | BR_SP_Csm01 | Unknown | BR_PA_Bel01 | - | + | + | + | + | + | + | 9.656 x 10^-08^ |
|  | BR_SP_Lim09 |  |  |  |  |  |  |  |  |  |  |
|  | BR_SP_SJP01 |  |  |  |  |  |  |  |  |  |  |
|  | BR_SP_SJP02 |  |  |  |  |  |  |  |  |  |  |
|  | BR_SP_SJP05 |  |  |  |  |  |  |  |  |  |  |
|  | BR_SP_SdM01 |  |  |  |  |  |  |  |  |  |  |
|  | BR_SP_SdM02 |  |  |  |  |  |  |  |  |  |  |
|  | BR_SP_SdM03 |  |  |  |  |  |  |  |  |  |  |
|  | BR_SP_SdM04 |  |  |  |  |  |  |  |  |  |  |
|  | BR_SP_SdM05 |  |  |  |  |  |  |  |  |  |  |
|  | BR_SP_SdM06 |  |  |  |  |  |  |  |  |  |  |
|  | BR_SP_SdM15 |  |  |  |  |  |  |  |  |  |  |
| 5 | BR_SP_SAP03 | Unknown | BR_PA_Bel01 | - | - | - | + | + | + | + | 2.631 x 10-9 |
| 6 | BR_PR_Mgf01 | Unknown | AR05 | - | + | + | + | + | + | + | 1.039 x 10^-12^ |
| 7 | PY02 | Unknown | BR_PR_Ldb01 | - | - | - | + | + | + | + | 7.689 x 10^-7^ |

^a^Method abbreviation = R: RDP; G: GENECONV; B: Bootscan; M: Maxchi; C: Chimaera; S: SiScan; and T: Topal.
