# Supplementary material for: Molecular Epidemiology of Citrus Leprosis Virus C: A New Viral Lineage and Phylodynamic of the Main Viral Subpopulations in the Americas
Source: Front Microbiol. 2021 Apr 29;12:641252. doi: 10.3389/fmicb.2021.641252 (PMC8116597; doi:10.3389/fmicb.2021.641252)
Supplement: Supplementary Table 5 — Summary of selection analysis of the p29 and p32 in CiLV-C. [file Table_5.docx]

**Supplementary Table S5**. Summary of selection analysis of the *p29* and *p32* in CiLV-C.

| **Method** | **Dataset** | **Selection pressure** | ***p*-value** | **Number of sites** | **Amino acid under selection** |
| --- | --- | --- | --- | --- | --- |
| ***p29*** | | | | | |
| FUBAR | SJP+CRD+ASU (190) | purification | 0.9 | 47 | 5, 7, 29, 30, 32, 33, 47, 58, 61, 69, 76, 99, 100, 101, 106, 112, 113, 116, 136, 137, 138, 139, 154, 157, 161, 165, 171, 173, 175, 181, 195, 196, 198, 201, 207, 208, 209, 213, 217, 219, 220, 227, 240, 244, 245, 254 and 259 |
|  | SJP+CRD (189) | purification | 0.9 | 45 | 5,7, 12, 29, 30, 32, 33, 48, 58, 61, 76, 100, 101, 106, 112, 113, 116, 136, 137, 139, 151, 154, 157, 161, 165, 173, 181, 195, 196, 198, 201, 202, 207, 208, 209, 213, 217, 219, 227, 234, 240, 244, 245, 254 and 259 |
|  | SJP (106) | purification | 0.9 | 17 | 7, 29, 48, 61, 68, 69, 76, 101, 137, 139, 157, 173, 198, 208, 219, 240 and 244 |
|  |  | diversification | 0.9 | 1 | 16 |
|  | CRD (83) | purification | 0.9 | 13 | 7, 32, 58, 99, 105, 111, 134, 159, 169, 179, 209, 215 and 225 |
| FEL | SJP+CRD+ASU (190) | purification | 0.1 | 53 | 7, 15, 23, 29, 30, 32, 33, 47, 58, 61, 69, 76, 83, 99, 100, 101, 106, 113, 116, 117, 136, 137, 138, 139, 147, 154, 157, 159, 161, 165, 173, 175, 181, 186, 194, 195, 196, 198, 201, 207, 208, 209, 211, 213, 217, 219, 220, 227, 240, 245, 251, 254 and 259 |
|  | SJP+CRD (189) | purification | 0.1 | 48 | 5, 7, 12, 13, 15, 29, 30, 32, 33, 58, 61, 76, 100, 101, 106, 113, 116, 136, 137, 138, 139, 151, 154, 157, 159, 161, 165, 173, 181, 195, 196, 198, 201, 202, 205, 207, 209, 211, 213, 217, 219, 220, 227, 234, 240, 245, 254 and 259 |
|  |  | diversification | 0.1 | 1 | 93 |
|  | SJP (106) | purification | 0.1 | 18 | 5, 7, 12, 29, 33, 38, 61, 68, 69, 92, 139, 157, 198, 209, 213, 234, 240 and 255 |
|  | CRD (83) | diversification | 0.1 | 1 | 149 |
|  |  | purification | 0.1 | 18 | 7, 32, 47, 58, 72, 87, 99, 101, 106, 107, 113, 136, 161, 167, 181, 211, 217 and 227 |
| MEME | SJP+CRD+ASU (190) | diversification | 0.1 | 6 | 16, 26, 53, 104, 140 and 163 |
|  | SJP+CRD (189) | diversification | 0.1 | 7 | 16, 26, 53, 93, 104, 140 and 163 |
|  | SJP (106) | diversification | 0.1 | 2 | 16 and 53 |
|  | CRD (83) | diversification | 0.1 | 3 | 26, 102 and 138 |
| FUBAR_FEL | SJP+CRD+ASU (190) | purification | 0.1 | 42 | 7, 29, 30, 32, 33, 47, 58, 61, 69,76, 99, 100, 101, 106, 113, 116, 136, 137, 138, 139, 154, 157, 161, 165, 173, 175, 181, 195, 196, 198, 201, 207, 208, 209, 213, 217, 219, 220, 227, 240, 245, 254 and 259 |
|  | SJP+CRD (189) | purification | 0.1 | 42 | 5,7, 12, 29, 30, 32, 33, 58, 61, 76, 100, 101, 106, 113, 116, 136, 137, 139, 151, 154, 157, 161, 165, 173, 181, 195, 196, 198, 201, 202, 207, 208, 209, 213, 217, 219, 227, 234, 240, 245, 254 and 259 |
|  | SJP (106) | purification | 0.1 | 9 | 7, 29, 61, 68, 69, 139, 157, 198 and 240 |
|  | CRD (83) | purification | 0.1 | 4 | 7, 32, 58 and 99 |
| ***p32*^a^** | | | | | |
| FUBAR | SJP+CRD+ASU (270) | purification | 0.9 | 24 | 68, 71, 72, 73, 74, 75, 76, 77, 80, 84, 93, 95, 96, 97, 98, 99, 103, 111, 112, 115, 138, 146, 147 and 148 |
|  | SJP+CRD (269) | purification | 0.9 | 19 | 68, 71, 72, 75, 76, 80, 84, 93, 96, 97, 98, 99, 100, 101, 103, 115, 146, 147, and 148 |
|  | SJP (190) | purification | 0.9 | 1 | 75 |
|  | CRD (80) | purification | 0.9 | 5 | 75, 97, 103, 112 and 136 |
| FEL | SJP+CRD+ASU (270) | purification | 0.1 | 33 | 57, 68, 70, 71, 72, 73, 74, 75, 76, 77, 79, 80, 84, 93, 95, 96, 97, 98, 99, 102, 103, 111, 112, 115, 119, 121, 126, 138, 142, 145, 146, 147 and 148 |
|  | SJP+CRD (269) | purification | 0.1 | 27 | 57, 68, 71, 72, 74, 75, 76, 77, 80, 84, 85, 93, 96, 97, 98, 99, 102, 103, 111, 112, 115, 119, 121, 145, 146, 147 and 148 |
|  | SJP (190) | purification | 0.1 | 10 | 57, 64, 68, 72, 75, 84, 119, 125, 145 and 146 |
|  | CRD (80) | purification | 0.1 | 14 | 68, 69, 73, 75, 77, 85, 93, 96, 97, 98, 103, 136, 146 and 148 |
| MEME | SJP+CRD+ASU (270) | diversification | 0.1 | 2 | 64 |
|  | SJP+CRD (269) | diversification | 0.1 | 1 | 64 |
|  | SJP (190) | diversification | 0.1 | 0 | - |
|  | CRD (80) | diversification | 0.1 | 0 | - |
| FUBAR_FEL | SJP+CRD+ASU (270) | purification | 0.1 | 24 | 68, 71, 72, 73, 74, 75, 76, 77, 80, 84, 93, 95, 96, 97, 98, 99, 103, 111, 112, 115, 138, 146, 147 and 148 |
|  | SJP+CRD (269) | purification | 0.1 | 17 | 68, 71, 72, 75, 76, 80, 84, 93, 96, 97, 98, 99, 103, 115, 146, 147, and 148 |
|  | SJP (190) | purification | 0.1 | 1 | 75 |
|  | CRD (79) | purification | 0.1 | 4 | 75, 97, 103 and 136 |

^a^The analyzed sequence of the ORF *p32* corresponds to the amino acid stretch between positions 55 and 150 of the complete MP protein.
